# Membrane-Traversing Peptides: Mechanistic Landscapes, Assays, and Applications

**DOI:** 10.1021/acs.chemrev.6c00060

**Published:** 2026-08-04

**Authors:** Adrian J. Taveras, Erin A. Kuang, Ryan P. Ferrie, Ace Ellis, Alaina Tait, Rayhanus Salam, Kalina Hristova, William C. Wimley

**Affiliations:** † Department of Biochemistry and Molecular Biology, 12255 Tulane University School of Medicine, New Orleans, Louisiana 70112, United States; ‡ Department of Microbiology and Immunology, 12255 Tulane University School of Medicine, New Orleans, Louisiana 70112, United States; § Institute for NanoBioTechnology, 1466 Johns Hopkins University, Baltimore, Maryland 21218, United States; ∥ Department of Materials Science and Engineering, Whiting School of Engineering, 1466 Johns Hopkins University, Baltimore, Maryland 21218, United States

## Abstract

Lipid bilayer membranes are essential cellular permeability barriers that strictly limit the passage of polar molecules and macromolecules, thereby compartmentalizing cellular biochemistry and limiting the accessible chemical space for therapeutics. Membrane-traversing peptides (MTPs) are a diverse group of peptides defined by the ability to traverse synthetic or cellular membranes without causing permeabilization and without the assistance of specific transport proteins. These peptides fundamentally defy classical thermodynamic models of membrane permeability as they can cross lipid bilayers and deliver large polar cargoes, including peptides and proteins, into cells, despite having high net charge and low hydrophobicity. In this review, we challenge the idea of single, discrete mechanisms to describe how peptides traverse membranes and define a broad mechanistic landscape shaped by the conserved properties of MTPs and the unique physical chemistry and polymorphic phase behavior of lipid bilayers. We describe experimental assays and model systems that can be used to study MTP translocation and cargo delivery in synthetic systems and living cells. We use information obtained from comprehensive databases to consider the shared physical chemical space of MTPs. We also discuss key unanswered questions regarding mechanism, specificity, endosomal escape, and *in vivo* performance. Finally, we discuss illustrative examples and emerging translational applications of MTPs in intracellular drug delivery and molecular therapeutics. Together, these perspectives underscore the dual importance of MTPs as probes for fundamental biophysical studies of cell membranes and as promising tools to expand the universe of druggable intracellular targets.

## Graphical abstract

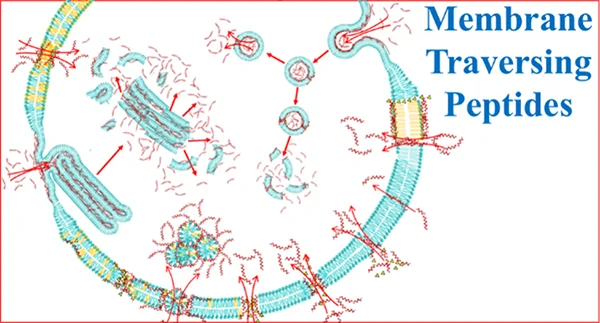

## 1 Introduction

### 1.1 What Is a Membrane-Traversing Peptide?

The lipid membrane, a ubiquitous feature of all known forms of life, serves as a selective permeability barrier to the many ions and polar compounds that comprise the aqueous biochemistry of living things.^14^ The barrier property of the membrane is derived from a core comprising a continuous nearly two-dimensional layer of almost pure hydrocarbon.^15,16^ Although this nonpolar layer is only two molecules, and a few nm, thick, it effectively prevents most polar molecules from spontaneously diffusing across the membrane. The normal movement of polar material (and information) across biological membranes is mostly accomplished by proteins, receptors, channels, pumps, and transporters,^17^ that have evolved for this function. Nonprotein-mediated passage across membranes is generally accomplished by small molecules which are partially soluble in both the bilayer hydrocarbon and the aqueous milieu.^18,19^ Exogenous small-molecule drugs are the small subset of biochemically active compounds that have this property, a requirement that severely limits the chemical space available for small-molecule drugs with intracellular targets.^18,19^

Yet, in the alternate chemical space of peptides, there are many thousands of examples that can cross membranes,^1,20–27^ and even deliver large, polar cargo across membranes without large-scale permeabilization. Delivery is readily accomplished by peptides that completely defy the normal rules that govern membrane crossing by small molecules.^18,19^ As per the literature, many of these peptides have been categorized as cell-penetrating peptides (CPPs). After years of discovery and characterization of peptide-mediated delivery, the term CPP has become too narrow to describe peptides with distinct differences from the classical polycationic CPPs. Here, we propose a new term to encompass the much larger library of these peptides. In this Review, we discuss the many classes of **membrane-traversing peptides (MTPs)**, which we define as ***any peptide that can move from one side of a synthetic or cellular membrane to the other side without gross permeabilization of the membrane and without requiring the action of specific proteins***. MTPs may greatly expand the druggable chemical space.^19–27^

### 1.2 Why Study Membrane-Traversing Peptides?

MTPs have potential utility in a wide variety of applications.^20–28^ Foremost, there has long been promise, but also disappointment, in the possible translational applications of MTPs for the delivery of useful polar compounds, including small molecules, peptides, proteins, and oligonucleotides and their analogues to cells. The ability to deliver polar and macromolecular compounds to cells would be highly useful in the laboratory and in the clinic, where it would significantly expand the universe of potential drug compounds. For example, many potentially important cancer therapy targets identified by the NCI and others^29–31^ are intracellular proteins. Most of these proteins are components of protein interaction networks (PINs), i.e., signaling networks that normally control cellular growth and differentiation.^32–34^ In cancer, PINs function abnormally due to the accumulation of mutations or altered expression that disrupts critical regulatory pathways. Thus, the protein–protein interactions that comprise PINs are critical therapeutic targets. Yet, PINs remain underutilized as targets because the molecular interfaces between proteins are nearly undruggable with small molecules,^35^ as they are generally unable to specifically bind to large, distributed protein interaction interfaces. Potentially ideal therapeutics for modulating PINs include macromolecules that can bind to one of the interacting partners, blocking or mimicking an interaction, or targeting the protein for degradation. Possible cargoes include interface-mimicking peptides, dominant-negative proteins, and antibodies and their smaller variants.^36–40^ Improvement in treatment outcomes may arise from MTPs that are engineered or evolved to deliver normally impermeant cargoes.

MTP research exists at the intersection of fundamental membrane biophysics and translational biomedical applications. The study of MTPs has changed our ideas about the impermeability of lipid bilayers and offers a framework for understanding how biological barriers can be bypassed without being disrupted. By examining the diverse mechanisms through which MTPs interact with, insert into, and traverse membranes, we deepen our understanding of the membrane structure and function. The study of MTPs also highlights possible transformative applications in drug delivery and intracellular targeting. The rational design and optimization of MTPs promise to greatly expand the universe of molecules that can access the intracellular space, potentially redefining the landscape of molecular medicine and therapeutic development.

## 2 The Physical Chemistry of Peptides and Membranes

Compartmentalization of soluble proteins and the myriad small polar molecules needed for a cell to function is one of the shared characteristics of all forms of life and is likely to have been an essential step in the *origin* of life.^34,41,42^ Archaea and prokaryotes have one or just a few compartments, while eukaryotes have an array of compartments and subcompartments, organelles, that are all simultaneously and separately maintained. Whatever the branch of life or specific partition, compartmentalization is accomplished by a lamellar membrane, a noncovalently self-assembled, nm-scale film with an aliphatic core that is very hydrophobic, bounded by polar interfacial regions that bridge the aqueous phase and the hydrocarbon core. The hydrophobicity of the hydrocarbon core and the cohesive interactions between the lipids are the physical−chemical bases for the inherent permeability barrier against small and large polar molecules.^14,16,19^

While the permeability barrier of membranes is essential, it cannot be absolute. Cell function requires that material, nutrients, and waste products, as well as information be able to cross the membrane, usually with the assistance of proteins evolved for this function.^17^ It also requires that proteins be able to function dynamically in the membrane and that proteins and lipids be able to diffuse in two dimensions to interact with needed partners.^43^ Thus, the structures and properties of all biological membranes are a compromise between their essential function as permeability barriers and the requirement that cells maintain active communication both across and within the membrane.

### 2.1 Properties of the Lipid Bilayer Membrane

The hydrocarbon core of a lipid bilayer membrane, the basis for its permeability barrier property, is one of the most hydrophobic environments found in nature. In a synthetic bilayer, the center of the hydrocarbon core is almost pure hydrocarbon with a near absence of water and other polar moieties.^7,44,45^ Yet the bilayer cannot be described as a simple slab of low dielectric hydrocarbon. It is a liquid crystal, with free diffusion in the plane of the bilayer, at least above the gel to liquid phase transition temperature, which is the normal state of the membranes of living cells.^43,46,47^ Along the direction of the bilayer normal (defined as the axis perpendicular to the surface), the bilayer is also dynamic, with significant excursions of polar groups, including water, deep into the bilayer interface and excursions of the hydrocarbon chains up toward the surface, dynamics that have been described as “tumultuous chemical heterogeneity”.^7,44^ These considerations lead to the realistic lipid bilayer depicted in Figure 1, a hydrocarbon layer in the center of the bilayer that is bounded on each side by broad interfacial zones. In these interfacial zones, the chemical composition describes a continuously changing gradient of polarity, connecting the highly polar bulk water phase with the nonpolar hydrocarbon core of the bilayer. The continuously changing interfacial zones are not simple boundaries between phases, but are broad regions of rich chemical diversity that enable a variety of structures and interactions to occur, including those critical to the actions of MTPs.

**Figure 1:**
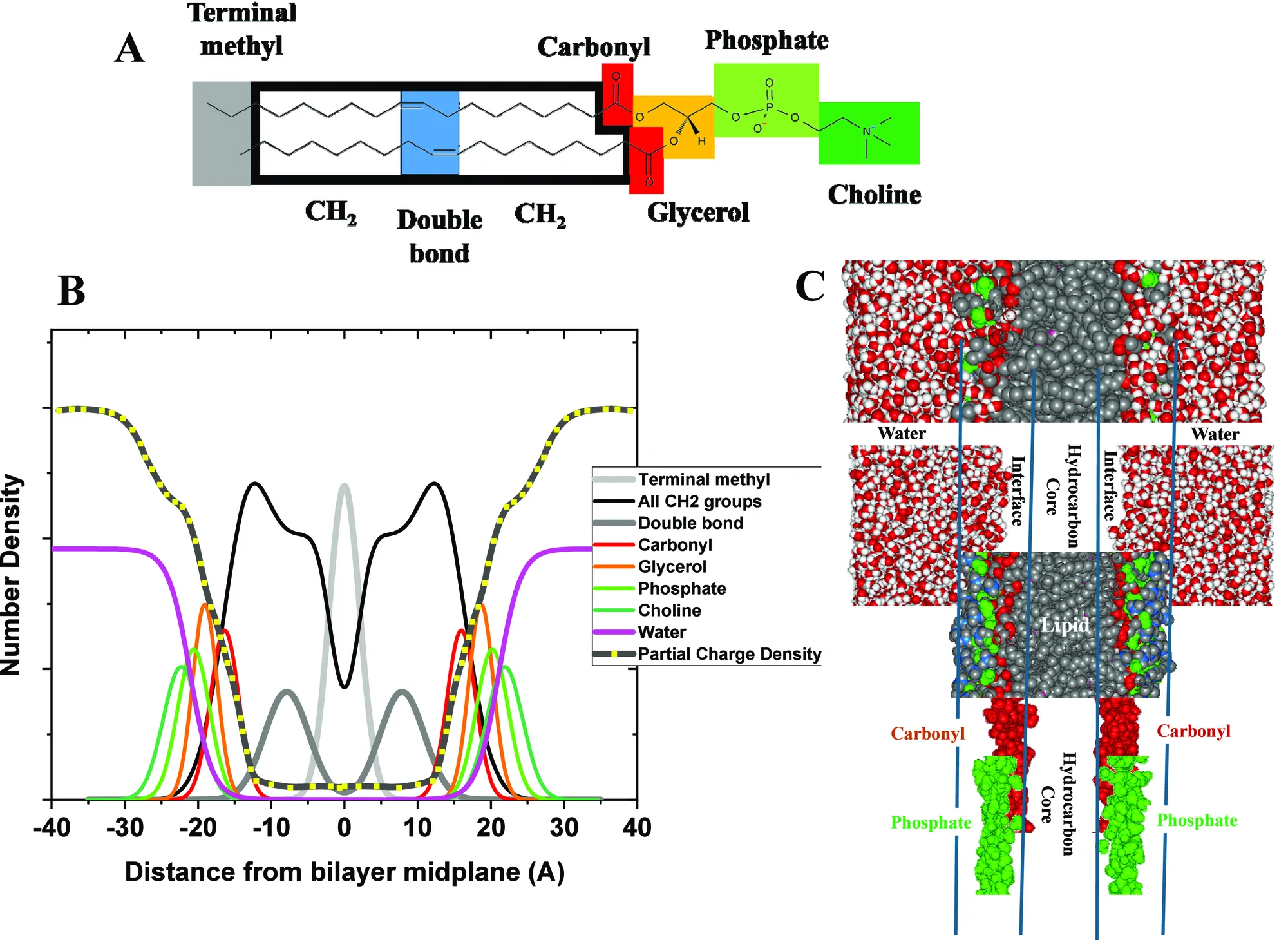
Physical chemistry of lipid bilayer membranes. (A) A phosphatidylcholine molecule with quasimolecular groups labeled. (B) Number densities of lipid quasimolecular groups across the membrane.^7–9^ The dotted line is the calculated partial charge density, a measure of the polarity distribution. (C) Structure of a fluid phase lipid bilayer membrane from a molecular dynamics simulation,^11^ deconstructed to show the zones occupied by the quasimolecular components. The lipid bilayer membrane is comprised of a nearly pure hydrocarbon core that is separated from bulk water by a broad and chemically complex interface.^7^ The interface is best described by a gradient of polarity bridging the extremes of bulk water and the hydrocarbon core.

### 2.2 The Polymorphic Phase Behavior of Lipids

The properties of biological membranes are dominated by lipids that self-assemble into lamellar bilayer phases. However, lipids have polymorphic phase behavior. They can adopt multiple physical states including nonlamellar phases that are micellar, tubular, hexagonal, inverted cubic, and more,^48,49^ depending on the lipid properties and the conditions. Biological membranes are comprised of mixtures of lipids that spontaneously form lamellar phases as well as lipids that can form nonlamellar phases.^50–53^ Such nonbilayer forming lipids enable disruption of the normally strict segregation between the hydrocarbon core and polar interfacial regions, the primary energetic barrier to peptide translocation. In biological membranes, this diversity contributes to the physical properties, such as spontaneous curvature, and permeability of the bilayer, influencing many essential processes, including the interactions of the bilayer with MTPs. Thus, the polymorphic phase behavior of lipids is an important aspect of the structural properties of biological membranes.

Lipid bilayers can also form stacked, parallel multibilayer phases^54,55^ or other three-dimensional structures^56–60^ that differ in important ways from free-standing, two-dimensional lamellar bilayers. As we discuss in detail below, membrane transitions from two-dimensional to three-dimensional states, and back again, may be the most obvious way^55,61–63^ to explain the mechanism by which MTPs can carry very large polar macromolecule cargoes from one side of a membrane to the other without causing membrane permeabilization.

### 2.3 Fundamental Principles of Peptide–Membrane Interactions

Peptide binding to membranes has been described using many different models, including models built on simple binding site concepts as well as cooperative models.^64–67^ Perhaps the most realistic mathematical description of membrane binding is a partitioning model in which the bilayer and the aqueous phase are treated as fluid phases. Peptides partition between these phases according to the total free energies of interaction.^68^ Such models have an infinite dilution realm, in which the partition coefficient is independent of the peptide-to-lipid ratio, but can be modified to include the effects of peptide concentration, such as when binding is cooperative^64^ or when charged peptides accumulate on a bilayer.^67,69–75^ Peptide–membrane interactions are mainly noncovalent and are not additive.^65,76^ They also include entropic contributions. For MTPs, the most critical of the interactions are hydrophobic and electrostatic ones, because without an obvious source of one or both, many MTPs will not interact with membranes.^20,22^

#### 2.3.1 The Hydrophobic Effect

The hydrophobic effect drives membrane binding, as hydrophobic side chains favor partitioning into the bilayer, to reduce the costly exposure of nonpolar surface area to bulk water.^77^ Deeper insertion into the interfacial gradient is preferred by hydrophobic groups, while charged and polar groups, including the peptide backbone, disfavor deeper insertion^78,79^ in a combined effect that is nonadditive.^65^ Various hydrophobicity scales have been developed to measure the contribution of hydrophobic groups to membrane partitioning.^68,79–85^ Two experimentally determined whole-residue hydrophobicity scales have been especially useful for modeling peptide–membrane interactions.^68,83,84^ They include a membrane interfacial scale for peptides partitioning into the bilayer interface^68,84^ and an octanol-partitioning scale^68,83^ that mimics partitioning into the hydrophobic bilayer core. These scales highlight the special role that aromatic residues have in the interfacial partitioning of MTPs as well as the coupling that occurs in some classes of MTPs between membrane binding and secondary structure.

#### 2.3.2 Electrostatic Interactions and the Effect of Lipid Composition

Electrostatics dominate the specificity and strength of peptide–membrane interactions of MTPs. Most biological membranes are anionic. Probably for this reason, most MTPs are cationic.^1^ Eukaryotic cell membranes have anionic phospholipids, mainly phosphatidylserine (PS) and phosphatidylinositol (PI), with small amounts of phosphatidylglycerol (PG) and phosphatidic acid. Importantly for understanding MTPs, these anionic phospholipids are normally sequestered on the inner monolayer of living cells and not surface-exposed,^86^ although they can become more exposed in some disease states.^87,88^ This has led to a disconnect between model system studies and cell-based studies, the former nearly always use anionic phospholipids, usually PS, to drive MTP binding to membranes, whereas initial MTP binding to cells likely does not rely on anionic phospholipids at all. More likely, MTPs initially bind to (i) anionic gangliosides, which are surface-exposed, (ii) the anionic carbohydrates on the proteoglycans of the extracellular matrix, and (iii) anionic cell surface glycoproteins. Accumulation of MTPs on anionic cell surface carbohydrates may be a prerequisite to their accumulation on the membrane itself. At the membrane, MTPs may initially interact with sialic acid-containing ganglioside lipids,^89,90^ which are surface-exposed and comprise 0.5–10% of plasma membrane lipids.^91,92^ Alternatively, some research has supported the idea that oxidized lipids drive the initial binding of MTPs to membranes.^93–96^ This effect may explain the light-induced uptake of some MTPs that occurs when imaging cells with laser scanning confocal microscopy.^96^ At the same time, the presence of cholesterol has strong inhibitory effects on membrane penetration by peptides.^97^

In any case, MTP binding to the plasma membrane, perhaps by interactions with gangliosides or oxidized lipids, may be sufficient to scramble the transbilayer asymmetry and allow the abundant inner membrane PS to equilibrate onto the outer leaflet,^98^ where it would enable additional MTPs to bind. Whatever the pathway, these strong electrostatic interactions with anionic lipids can drive changes in bilayer ultrastructure by inducing curvature,^99–102^ by causing phase separation, or by driving the formation of stacked multibilayer structures or peptide–lipid particles.^55^

#### 2.3.3 Secondary Structure Formation

In many families of membrane-active peptides, structure formation is closely coupled to membrane binding^68,103^ because the free energy cost of open backbone hydrogen bonds is high when peptides transition from bulk water to the bilayer-bound state,^104,105^ especially when the partitioning is driven by the hydrophobic effect. The membrane interface can readily accommodate folded α-helical structures^106^ and the energy cost of backbone polar groups is substantially lower if they are H-bonded.^68,84^ This binding-folding coupling is relevant to some MTPs, such as penetratin,^107^ but many other MTPs, such as polyarginine peptides, are active as unstructured peptides.

#### 2.3.4 Comparison of Membrane Interactions of Drugs and Peptides

The membrane translocation of small-molecule drugs is fundamentally a thermodynamic process that can be described by the solubility of the drug in the hydrophobic core of the lipid bilayer.^18,19,108^ Accordingly, drug log *P* values, which measure hydrophobicity through octanol–water partitioning, are fairly predictive of membrane translocation rate by passive diffusion. These principles are the basis for the effectiveness of Lipinski’s rules of five^18^ to explain the drug-likeliness of small molecules.^19^

The Wimley–White octanol-based hydrophobicity scale for peptides is based on the same measurement as log P values used to characterize potential small-molecule drugs.^18^ On this basis, many MTPs defy the simple thermodynamic argument that works for drugs since they cross membranes despite being highly polar and charged and not hydrophobic.^1,20–28^ Perhaps the best example of this is the ability of polyarginine peptides to rapidly cross cell membranes and deliver cargo,^25,109–113^ despite the fact that they have many charged cationic guanidino groups and no hydrophobic side chains. According to measurements, simulations, and thermodynamic arguments, the movement of single arginine across a membrane should be highly unfavorable.^81,114,115^ Yet, in MTPs, arginine is the most common amino acid,^1^ and increasing the number of arginines generally increases the translocation rate. This fact is inconsistent with a simple thermodynamic argument for MTPs. Instead, they likely cross membranes by a fascinating broad array of mechanisms by which a highly polar peptide can move from one side of a bilayer to the other. These mechanisms are unified by their dependence on the unique physical chemistry and membrane-scale dimensions of MTPs, combined with the polymorphic phase behavior of the membrane lipids.

## 3 The Mechanistic Landscape of Membrane-Traversing Peptides

Peptides that interact with membranes display a broad range of behaviors, including transient surface association, deep insertion, folding, self-assembly, and membrane remodeling.^20–27,68,116,117^ These behaviors are coupled to the many ways that lipid bilayers can respond to peptide binding, insertion, and conformational change. As discussed previously, the conformations and functions of membrane-active peptides are often not well described by single discrete structures or narrowly defined mechanisms. Instead, peptide–membrane interactions are more accurately represented as a multidimensional continuum of states and pathways, which we have termed a **mechanistic landscape.** ^22,116^

The mechanistic landscape framework captures the intrinsic plasticity and heterogeneity of peptide–membrane interactions. A single peptide sequence can populate multiple regions of this landscape simultaneously, and the distribution of states depends strongly on experimental conditions, including membrane lipid composition, peptide concentration, temperature, pH, ionic strength, and many other environmental variables. We have hypothesized^22,116,118^ that apparent inconsistencies in the literature on membrane-active peptides arise in part because experimental observations have often been interpreted in terms of singular pathways. In contrast, the mechanistic landscape perspective provides a unifying conceptual framework in which membrane-active peptides access a continuum of overlapping interaction modes and membrane perturbation pathways. We note that there is substantial mechanistic overlap between MTPs and other classes of membrane-active peptides, including antimicrobial peptides (AMPs), fusion peptides, and toxins, because these systems share many physicochemical properties and perturb membranes in related ways.^119,120^

Figure 2 schematically illustrates some proposed mechanisms of membrane translocation by MTPs, spanning processes mediated by individual peptides to the formation of large three-dimensional peptide–lipid assemblies.

**Figure 2:**
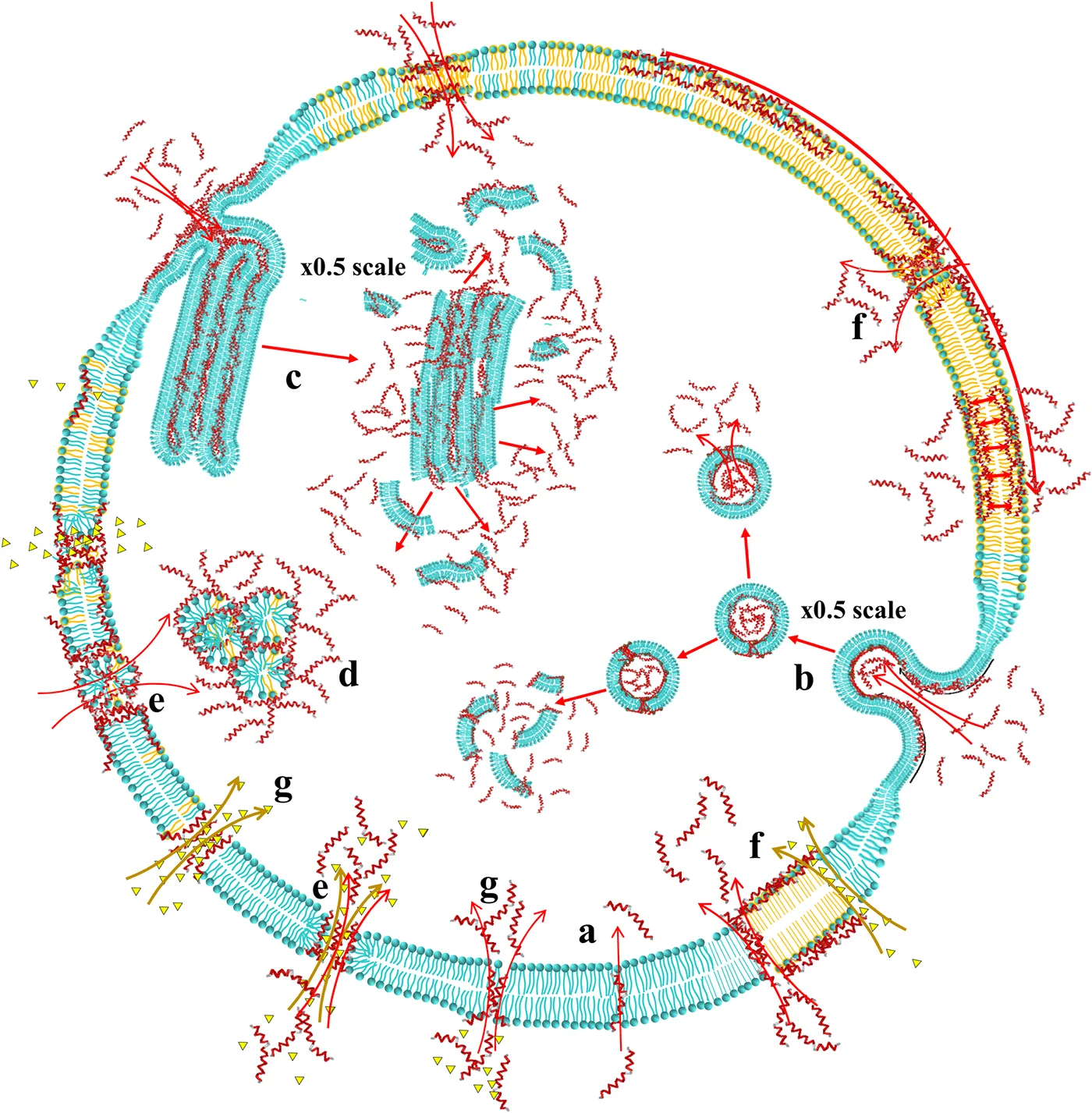
Membrane traversing peptides (MTPs) have been proposed to cross membranes by a broad variety of overlapping and nonexclusive mechanisms. Here, we schematically represent some classes of mechanisms that have been proposed by researchers in the field. These images are cartoon representations, not meant to be realistic molecular images. Some represent transient nonequilibrium structures. These mechanisms range from (a) simple monomeric peptide–membrane interactions to (b) those depending on endocytosis or (c) vesiculation, or (d) complex three-dimensional structural intermediates comprised of peptide and lipid in lamellar and nonlamellar form. Some mechanisms (e) rely on nonbilayer lipids at the translocation site. (f) The effects of lipid composition and lipid phase are represented by having blue and yellow lipids. (g) Membrane permeabilization is represented by yellow triangles, representing solutes, found on both sides of the membrane. Some larger structures, (b) and (c) are shown at 0.5 scale.

### 3.1 The Axes of a Mechanistic Landscape for MTPs

For the purpose of discussing the mechanisms of MTPs in this Review, we envision the mechanistic landscape that is shown in Figure 3. This landscape is dominated by two independently variable factors, the axes in Figure 3, that can be used to describe the rate-limiting, high-energy intermediate membrane structures in the process: dimensionality and anisotropy. **Dimensionality** is used to describe whether the rate-limiting intermediate membrane state in membrane translocation is approximately two-dimensional, as a peptide or peptides acting on a single bilayer, or whether the behavior is approximately three-dimensional, involving lipid-rich multibilayer vesicles, particles, or aggregates. The second axis, **anisotropy**, describes the degree of bilayer-like order in the critical lipid molecules of the rate-limiting intermediate state. Lipids can be anisotropic, as in a lipid bilayer, with unperturbed structure, or isotropic, as in a phase with highly disordered lipids in micellar or other nonlamellar states. Most importantly, this mechanistic landscape for MTPs includes many ways in which the lipids actively participate in the process of translocation, including the formation of three-dimensional and nonbilayer structures.

**Figure 3:**
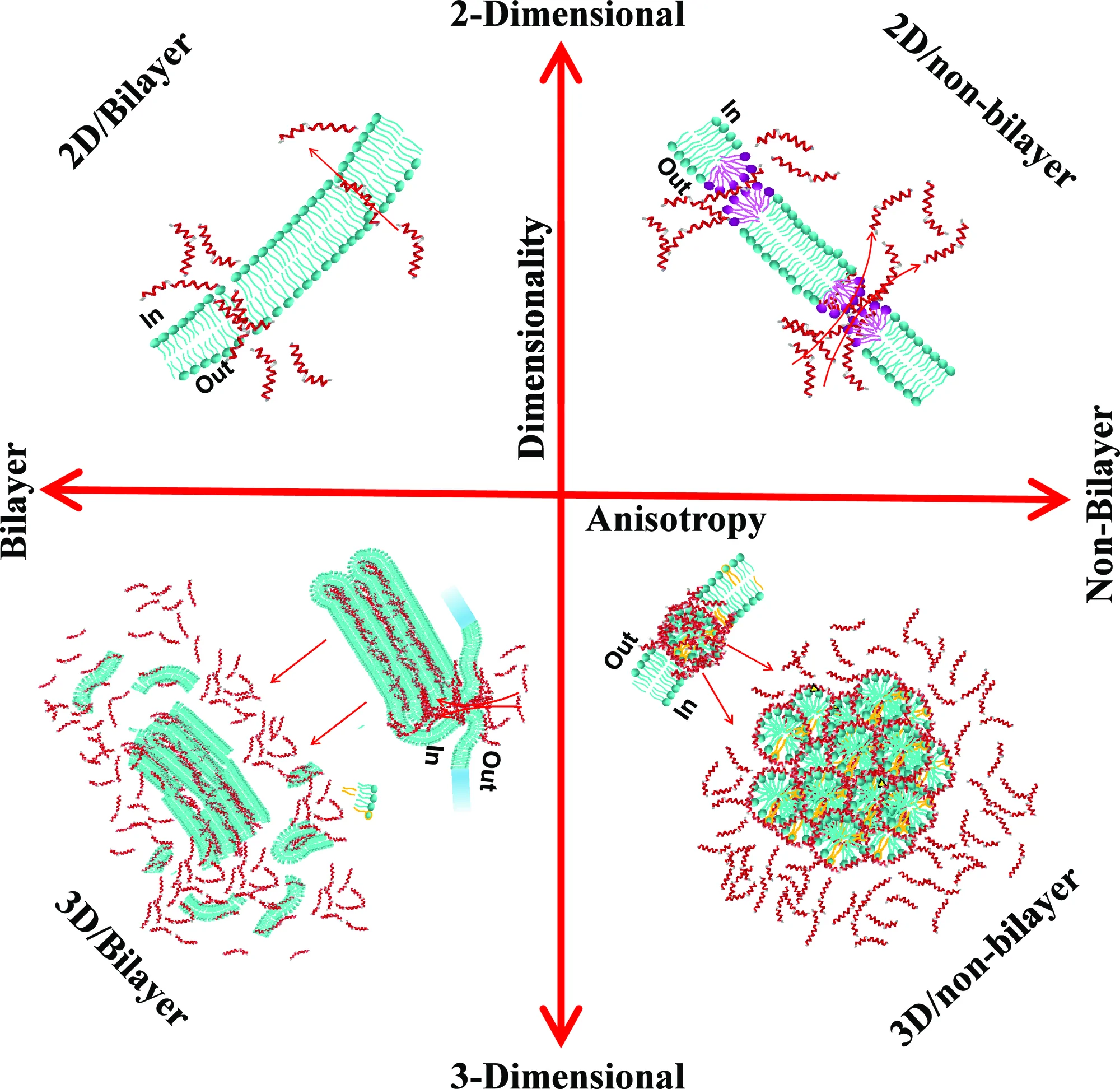
Mechanistic landscape of membrane traversing peptides (MTPs) can be described schematically in two dimensions using the orthogonal axes of **anisotropy** and **dimensionality** of the rate-limiting state of the system. Both the lower left and lower right quadrants can include processes that give rise to membrane connected puncta that are visible in fluorescence microscopy.

For MTPs, formation of the various states in Figure 3 occurs because MTPs have molecular dimensions that are similar to the total thickness of the bilayer and because they can have multivalent electrostatic interactions between cationic peptide groups and anionic lipid groups. In contrast, small-molecule drugs, which are neither membrane-sized nor polycationic, cross membranes by passive diffusion, which is controlled by classical hydrocarbon solubility.^19^

### 3.2 How do Peptides Cross Membranes?

In the subsections below, we discuss the mechanistic landscape of MTPs in terms of the four quadrants of Figure 3 defined by the **dimensionality** and **anisotropy** axes introduced above.

#### 3.2.1 Two-Dimensional and Lamellar Bilayer-Upper Left

In the **upper-left quadrant of the mechanistic landscape**, Figure 3, peptides interact with membranes in ways that preserve the lamellar architecture and lipid anisotropy to maintain a planar, two-dimensional geometry locally where the peptides interact. In this regime, peptides may bind to the membrane surface through the hydrophobic effect and/or electrostatic interactions and may undergo translocation, without triggering major alterations to the local organization of the bilayer. Although transient water defects or small perturbations may form as peptides progress toward translocation, in this quadrant, lipids remain lamellar in character and do not require restructuring of the lipid matrix. Because only a limited region of membrane needs to be affected by peptides, mechanisms in this quadrant may be cooperative, requiring the actions of multiple peptides, or noncooperative, with one or a few peptides sufficient to generate the necessary perturbations.

Some cationic peptides described in the literature cross membranes without causing major perturbation of the bilayer structure, so they likely belong to the upper left quadrant.^10,121–124^ For example, we selected members of one such family in a high throughput screen, calling them spontaneous membrane translocating peptides (SMTPs).^10,121,125,126^ The well-studied SMTPs TP1 (translocating peptide 1), TP2, and TP3 ^10,121,125–127^ translocate across both synthetic bilayers and cell plasma membranes at low concentration without bilayer disruption.^10,125,126^ They do not require aggregation or self-assembly to translocate across bilayers, as they readily translocate at very low concentrations (peptide:lipid ≤ 1:1000)^10,125^ across anionic and zwitterionic bilayers. Despite their simplicity, TP2 and related family members can deliver bioactive peptide cargoes to the cytosol of living cells.^127^ Similarly, cyclic analogs of the spider peptide Gomesin have been shown to translocate into cells at low concentrations.^124^ This behavior is very different from many other well-known MTPs that are active only above a specific threshold.^5,6,122,123,128–131^

Members of the SMTP family that we discovered preferentially have arginine residues over other basic amino acids. These arginines are found in conserved cationic/hydrophobic motifs exemplified by LRLLR.^10,126^ Such arginine-containing motifs may enable membrane crossing because the geometry and spacing of their guanidinium groups match the width of the bilayer interface, allowing these arginine guanidinium groups to remain coordinated to lipid headgroup phosphates throughout translocation.^3,132–135^ This is shown schematically in Figure 4. In this model, each arginine forms bidentate hydrogen bonds with lipid phosphate oxygens, a relatively stable, directional interaction that minimizes direct contact between guanidinium groups and the hydrophobic core. Instead, arginine residues may “hop” between adjacent headgroups by maintaining persistent interfacial binding and structured hydrogen-bonding networks.^8,10,12,125,136,137^ These interactions enable membrane crossing without the desolvation energetic penalties normally associated with charged residues in the hydrocarbon layer.^114^ A chaperoned, phosphate-guided mechanism may help explain why arginine-rich peptides consistently outperform lysine-rich analogs in membrane translocation: the guanidinium group possesses unique planarity, delocalized positive charge, and phosphate binding capability, enabling it to exploit the bilayer architecture as a productive pathway for transport. In support of this idea, it has been shown that fatty acids in combination with a transmembrane pH gradient, as well as pyrene butyrate, can chaperone arginine-rich peptides across the bilayer.^138,139^

**Figure 4:**
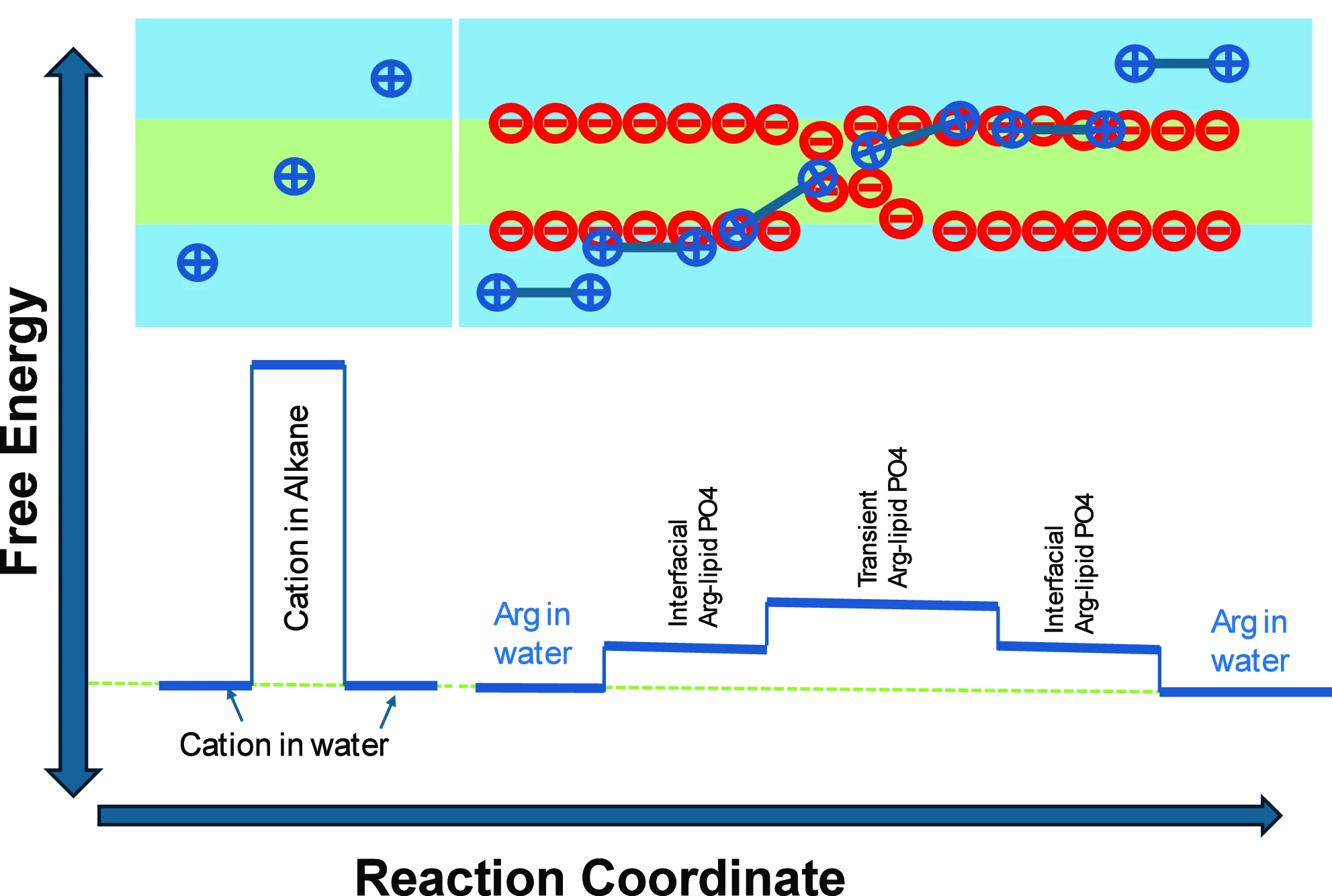
Energy barriers to crossing membranes. **Left**: Classical transport of a cation across a membrane is limited by the very low solubility of the cation in the bilayer hydrocarbon core. Energies are qualitative. **Right**: Transport of an arginine-containing peptide across the bilayer may depend on the fact that MTPs are similar in size to the dimensions of the membrane. The consequence of this is that the peptide cationic groups may be chaperoned across the membrane by lipid phosphates,^12^ which can dramatically lower the cost of arginine, or other charged amino acids, in the membrane by never requiring them to interact directly with the lipid hydrocarbon.

#### 3.2.2 Two-Dimensional with Local Nonbilayer Structure-Upper Right

The **upper-right quadrant of the mechanistic landscape**, Figure 3, is where peptides act on a single bilayer surface, but the rate-limiting state involves lipids that locally adopt nonbilayer structures such as locally disordered regions of isotropic, nonbilayer phase or inverted micellar lipids. In this quadrant, the membrane loses its lamellar order in a localized, two-dimensional context, enabling peptides to cross by stabilizing structures such as toroidal pores^140–142^ or inverted micelles that would otherwise be energetically unfavorable. Strongly amphipathic peptides and peptides with high cationicity can induce this behavior by enabling lipid headgroups to attain nonbilayer geometries.^143^ Nonbilayer structures lower the energetic barrier to membrane crossing, allowing peptides to traverse the hydrophobic core through disordered, curvature-rich zones^102^ without contacting the hydrocarbon core directly and without requiring desolvation of peptide polar groups. Cooperativity in this quadrant can range from minimal to moderate: some peptides may be able to nucleate nonbilayer intermediates alone, while others could require clustering to generate sufficient curvature stress, phase separation, and other effects.

Many translocation pathways previously interpreted as being dependent on pore formation may belong to the upper right quadrant. There is evidence that MTPs affect the bilayer physical properties and phase behavior. For example, bilayer curvature can be affected,^52,110,128,144^ domain structure can change,^52,145,146^ domains of clustered lipids and peptides can form,^52,145^ nonbilayer phases can form,^55,143,145,147–149^ bilayer disorder can increase^144^ or decrease,^150^ bilayers can undergo aggregation and fusion,^151^ and trans-leaflet flip-flop of lipids can occur.^143^

#### 3.2.3 Three-Dimensional and Multilamellar Bilayers-Lower Left

The **lower-left quadrant of the mechanistic landscape**, Figure 3, describes mechanisms in which dimensionality increases, as peptides interact with multiple lamellar structures simultaneously. Structures may include stacked bilayers, vesicles, or multilamellar vesicles. In this quadrant, the lipids maintain mostly bilayer-like anisotropy but deviate from a planar bilayer architecture as the three-dimensional architecture of peptide-rich areas of membrane becomes central to the translocation process.

Recruitment of anionic lipids via diffusion is likely required, as well as recruitment of additional peptides.^52,145^ Some configurations of stacked bilayers, Figure 2, require regions of high curvature at the edges of the stacks, which can be stabilized by cationic peptides.^52,110,128,144^

Experimental observations strongly support these mechanisms. For example, high local concentrations of cationic peptides can bring anionic bilayers together,^55,151^ generating multilamellar stacks. Peptide-driven unilamellar-to-multilamellar transitions and regions of high curvature are observed in model membranes^151^ and cell membranes.^61–63^ These ideas are reinforced by the architecture of eukaryotic membranes, which contain more than twice the lipid surface area required to enclose the cell, enabling abundant folded, stacked, and curved membrane structures to exist.^152,153^ Such structures are essential for secretion, endocytosis, and membrane maturation.^152,153^

A key observation supporting multilamellar mechanisms in the peptide-mediated delivery of cargoes to the cell is the delivery of large macromolecular cargoes. Dye-labeled peptides and peptide–macromolecule conjugates often enter cells with comparable efficiencies despite vastly different cargo properties, an outcome that rules out single-bilayer translocation. This behavior is consistent with intermediate structures that internalize aqueous lumen that can contain cargo, including macromolecules.^61–63,151^ Additional evidence comes from studies showing that peptides can sometimes drive the internalization of unconjugated proteins,^154^ implying that entry proceeds via engulfment of an aqueous compartment rather than direct molecular transport across a bilayer. In this interpretation, peptides drive the formation of transient multilamellar buds or collapsing membrane structures that capture dissolved cargo and subsequently release them into the cytosol. This mechanism can operate in the plasma membrane as well as in endosomal membranes. Pei and colleagues have observed peptide entry into cells through both plasma and endosomal membranes that have the characteristics of this quadrant, i.e. entry of peptides and cargo to cells via large particles comprised of peptides and bilayers. They have shown that these particles have vesicle-like properties such as encapsulation of aqueous media and the ability to bud off from cell membranes. These authors have called this process “vesicle budding and collapse”.^55,61–63,155^

#### 3.2.4 Three-Dimensional and Local Nonbilayer Structure-Lower Right

The **lower-right quadrant of the mechanistic landscape**, Figure 3, represents the most extreme region of the mechanistic landscape, where peptides self-assemble and drive lipids into three-dimensional, isotropic, nonbilayer structures. This regime is dominated by large peptide-lipid aggregates that provide alternative routes for translocation. In support of this possibility, peptides with strong amphipathicity, high cationic charge, or pH-responsive conformational changes can destabilize membranes to the point where bilayer packing changes as the lipids reorganize into highly curved, disordered structures.^55,143,145,147–149^ These reorganized lipid structures may behave as quasi-particulate intermediates that allow peptides and cargo to move across or between membranes through pathways that do not resemble traditional pore formation or insertion.^61–63^ Because these transitions require extensive lipid rearrangement, they exhibit very high cooperativity, typically arising only when peptide concentrations, local environmental conditions, or membrane stresses reach threshold values. Thus, lipid and peptide recruitment into concentrated areas is a prerequisite. Indirect support for this mechanism can be found in the literature. For example, MTPs can induce nonbilayer phases to form^55,143,145,147–149^ and can cause bilayer disorder to increase.^144^ If driven far enough, these effects can create nonbilayer phases of peptide–lipid particles. However, direct evidence for three-dimensional nonbilayer phases in cell membranes is scarce. In cellular systems, this quadrant may be especially relevant for endosomal escape, where acidification, extreme curvature, elevated peptide concentration, and altered lipid composition may enable membranes to form nonlamellar structures.

### 3.3 Two Parallel Pathways into Cells

The most common experimental classification of MTP entry in the literature is to assess the degree to which they use two parallel, nonexclusive pathways: endocytosis (energy)-dependent and endocytosis (energy)-independent. We note that both of these pathways are described by the mechanistic landscape of MTPs shown in Figure 3 because both require that a membrane be traversed and both are subject to the physical chemistry of peptide–membrane interactions. The distinguishing factor is whether membrane crossing is triggered by changes that take place in the endosomal compartment or endosomal membrane after energy-dependent peptide/cargo uptake, or whether the plasma membrane can be crossed directly without cellular energy.

**Energy-dependent delivery** occurs when an actively internalized (endocytosed) peptide or peptide–cargo chimera is triggered by changing conditions in the endosome to cross the endosomal membrane or to rupture the endosome and enter the cytosol. This path operates only when the cell is energized and functioning normally. Thus, energy-dependent delivery is blocked at room temperature or below, or when the cell is depleted of ATP by toxins such as rotenone. Energy-dependent delivery operates on the time scales of endocytosis, tens of minutes to hours. Because endosomes normally remain separate from the cytosol, delivery should be defined by the fraction of internalized peptide and/or cargo that ultimately escapes into the cytosol, a value that is often very small, especially at low peptide concentration.^61–63,156–161^ Of note, some potential cargoes, especially linear, unstructured peptides, are highly susceptible to endosomal proteolysis and may not survive energy-dependent uptake long enough to be delivered intact to the cytosol.

**Energy-independent delivery** occurs when a peptide and cargo directly cross the plasma membrane into the cell, independent of endocytosis. Direct entry can be accomplished either by peptides that translocate directly across the plasma membrane as monomers or by peptides that act cooperatively on the membrane, including, in extreme cases, peptides that trigger the formation of large μm-sized membrane-connected particles.^21,61–63^ MTPs that act as monomers, which are uncommon, are expected to cross membranes at all concentrations. MTPs that act cooperatively, which are common, will function only above a threshold concentration.^5,6,122,123,128–130^ Energy-independent delivery can be very fast, occurring on time scales of tens of seconds to minutes.^5,61^ This pathway is active even when endocytosis is strongly suppressed,^5,125^ such as when the cells are at 21 or 4 °C. Molecules that are susceptible to endosomal proteolysis, such as linear peptides, can be delivered successfully by this mechanism.

Energy-dependent and -independent delivery are not always distinct and separable mechanisms. Many peptides utilize both pathways in parallel, with a preference that can shift with peptide properties, membrane properties, and experimental conditions such as peptide concentration.

## 4 Techniques for Studying Membrane-Traversing Peptides

We discuss next the many experimental assays that have been developed to investigate membrane translocation, uptake, localization, and mechanisms of action of MTPs. These approaches include structural and functional studies in simplified synthetic membrane systems, as well as studies in living eukaryotic and prokaryotic cells. These many approaches offer distinct and complementary insights into MTP-membrane interactions. While synthetic membranes enable controlled dissection of fundamental biophysical processes and structure, cellular assays enable the researcher to probe the true complexity of biological membranes and intracellular trafficking, as well as the response of the cell to peptides. Importantly, results from these systems do not always align, reflecting critical differences in factors such as membrane composition, dynamics, and cellular response that must be considered when interpreting MTP function.

### 4.1 Synthetic Membrane Assays

Synthetic lipid bilayers are widely used model systems for investigating the mechanisms by which MTPs interact with and traverse membranes. Unlike cellular membranes, which are compositionally complex, asymmetric, and dynamically coupled to proteins and active cellular processes, synthetic membranes provide simple and well-defined properties. This allows individual parameters, such as lipid charge, fluidity, and phase behavior, to be systematically varied. As a result, synthetic bilayers provide mechanistic insights that are often difficult to obtain in living cells, serving as a complementary platform for dissecting fundamental principles of MTP activity. Next, we detail many assays that have been developed to study MTPs in synthetic membranes.

#### 4.1.1 Large Unilamellar Vesicle Assays

Large unilamellar vesicles (LUVs) of approximately 100–200 nm, prepared by extrusion through polycarbonate membranes,^162,163^ are widely used as simplified model membranes to study interactions between MTPs and lipid bilayers.^164^ In most peptide-focused studies, vesicles are composed of zwitterionic phosphatidylcholine (PC) mixed with anionic lipids such as phosphatidylserine (PS), phosphatidylglycerol (PG), phosphatidylinositol (PI), gangliosides, and other lipids such as cholesterol. Many lipid compositions have been studied. Because most MTPs are cationic, anionic lipids are generally required for membrane binding and translocation. Peptide-to-lipid ratios and/or the relevant concentration metric can be varied over many orders of magnitude using synthetic systems.

When a cationic peptide binds to an anionic vesicle, multiple outcomes are possible, including permeabilization, vesicle aggregation, membrane fusion, formation of nonbilayer phases, and translocation. Many of these effects describe the disruption of the normally strict segregation between the hydrophobic and hydrophilic regions of the bilayer. Thus, peptide translocation across a membrane will necessarily accompany activities, such as permeabilization and fusion. Below, we focus on assays that have been used to measure translocation without permeabilization.

Matsuzaki and colleagues^165^ developed a method to measure translocation by using lipid-attached quenchers and two populations of LUVs. This method has also been used by Elmore.^166^ Briefly, peptides are incubated with one population of vesicles that contains a lipid quencher of tryptophan fluorescence. This is followed by the addition of a large excess of a second population of vesicles lacking the quencher. Equilibration of the peptides among the vesicles changes their fluorescence. By comparing quenching after sequential vesicle addition to that observed with the simultaneous addition of both types of vesicles, it is possible to measure the amount of peptide that is not available for exchange. This is presumably the population of peptides that have translocated inside the vesicles during the initial incubation.

Marks et al.^10^ developed an orthogonal high-throughput assay that enabled simultaneous measurement of peptide translocation and membrane permeabilization, Figure 5. For translocation, they synthesized a peptide library in which each member contained an aminomethylcoumarin (AMC) group attached to a C-terminal phenylalanine. This created a reporter that was sensitive to the protease chymotrypsin, which would cleave the AMC group and change its fluorescence properties. To take advantage of this reporter, Marks et al. created LUVs with entrapped chymotrypsin. To block the activity of any released chymotrypsin, they added α-1 antitrypsin, a protein inhibitor, to the external solution. The AMC reporter would thus report only on translocation of peptides into the vesicles without gross membrane permeabilization. To measure permeabilization in the same vesicles, they entrapped the lanthanide metal terbium, complexed with citrate. To the external solution, they added pipecolinic acid (DPA), which complexes with Tb^3+^ to generate a bright fluorescence. Neither terbium nor DPA cross intact membranes, so complex formation only occurs if the membrane is permeabilized. AMC fluorescence and Tb/DPA fluorescence can be measured independently using a fluorescence plate reader. Using this assay, Marks et al. screened 24,000 peptides for translocation and leakage and identified a conserved family of SMTPs that silently translocate across lipid bilayers, including zwitterionic phosphatidylcholine bilayers, without permeabilization or disruption of membrane structure.^121^ These peptides have also been shown to spontaneously translocate across the plasma membranes of cells.^125^ Two later publications^3,126^ described a variant of this assay in which the entrapped chymotrypsin cleaved a C-terminal fluorophore from translocating peptides, which was subsequently detected by HPLC.

**Figure 5:**
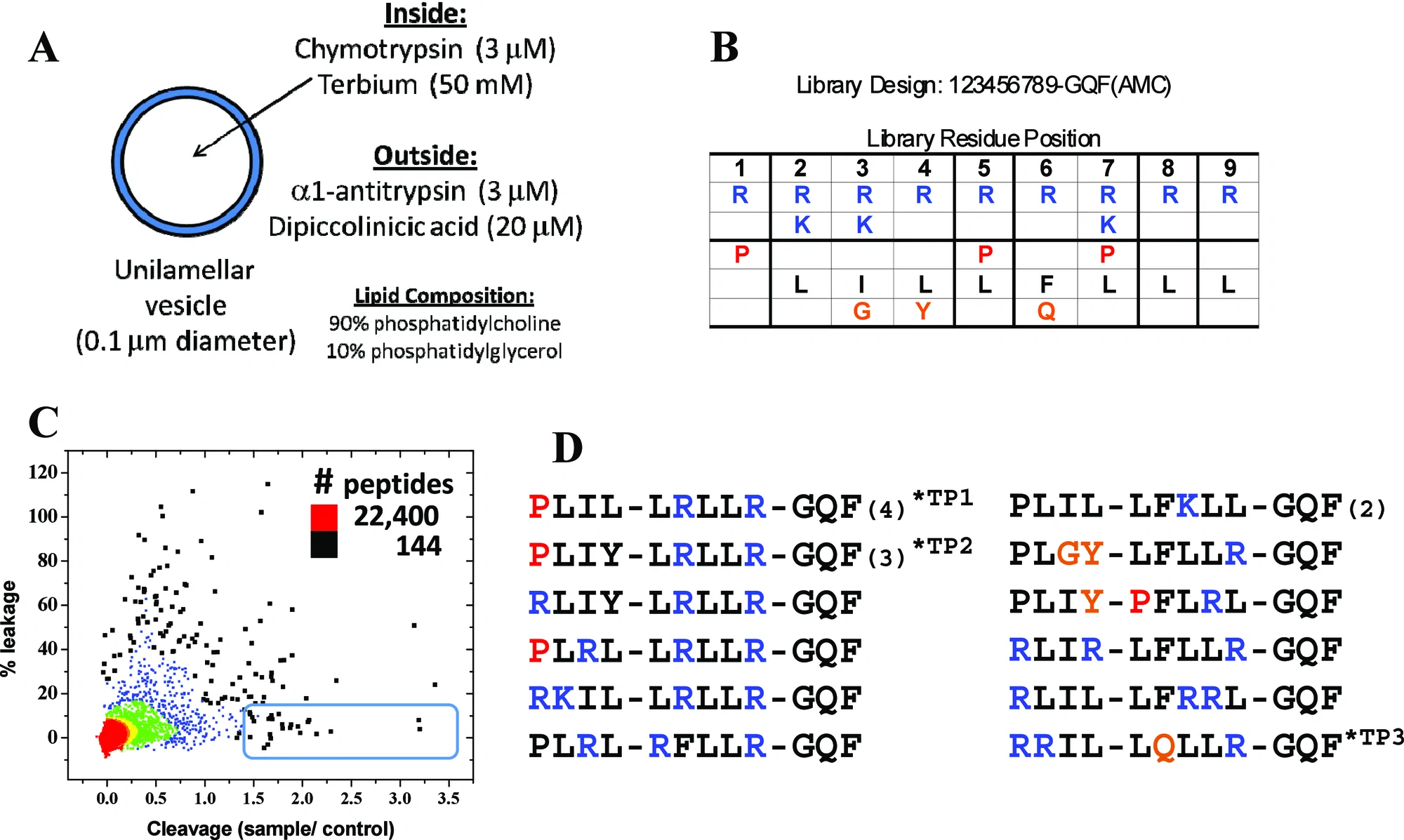
Synthetic molecular evolution of spontaneous membrane translocating peptides.^10^ (A) The orthogonal translocation and permeabilization assay developed to screen for MTPs using synthetic vesicles. (B) The combinatorial library that was screened in the referenced work. (C) Results of screening more than 24,000 library members. Positive hits with high translocation and low permeabilization are in the box. (D) The sequences of the spontaneous translocating peptides identified in this screen. Reproduced with permission from ref 10. Copyright 2011 American Chemical Society.

Alves and colleagues^167^ developed a method that uses protease protection and mass spectrometry to measure the translocation of peptides into LUVs. Briefly, they incubated biotinylated peptides with LUVs for varying amounts of time, after which the solutions were incubated with trypsin to digest peptides in solution or superficially bound to the surface of the liposomes. After just a few minutes, the trypsin was inhibited to reduce the cleavage of internalized peptides that translocated back out into solution. The liposomes were then disrupted, and the biotinylated peptides and fragments were captured and analyzed with mass spectrometry to measure the amount of full-length peptide. Only peptides that translocated into the liposomes before the trypsin was added were protected from proteolysis.

Silvius and colleagues,^168^ and later Swiecicki et al.,^128,129^ adapted an assay for the study of membrane translocation of peptides. The authors conjugated a 7-nitrobenzo-2oxa-1,3-diazole (NBD) fluorophore to peptides as a probe. Because NBD is small, uncharged, and not highly polar, it is less likely to alter peptide behavior than large, anionic visible dyes, such as fluorescein. NBD fluorescence is abolished when NBD is reduced by the membrane-impermeant reducing agent dithionite. Silvius showed that peptide translocation can be measured after incubation of NBD-labeled peptides with liposomes by measuring the fraction of peptide that is protected from quenching by dithionite. Importantly, membrane disruption can allow the dithionite to enter the vesicle interior; thus, this method only reports on translocation that is not accompanied by significant membrane permeabilization.

Hennig and colleagues^4^ developed an innovative fluorescence-based methodology that uses supramolecular host–dye reporter pairs to monitor peptide transport across lipid membranes, Figure 6. Macrocyclic hosts encapsulated within vesicles form noncovalent complexes with a fluorescent dye, resulting in a characteristic emission. Peptide entry into the vesicle is detected indirectly through competitive binding, which displaces the dye and induces a measurable fluorescence change. Membrane permeabilization is measured separately with entrapped dyes. Importantly, this label-free approach avoids chemical modification of peptides and preserves their native transport behavior. Peptide transport was quantified using steady-state and time-resolved fluorescence spectroscopy in LUV suspensions. Changes in fluorescence intensity provided kinetic information on peptide translocation as a function of concentration and peptide structure. Control experiments confirmed that fluorescence modulation arose from host–guest displacement rather than membrane disruption or direct dye–peptide interactions. Overall, the combined use of supramolecular sensing, ensemble spectroscopy, and single-vesicle imaging establishes a very useful method for studying membrane translocation. These authors used their method to study TP2, one of the peptides discovered by Marks et al.^10^ (see above), and verified its potent, “silent” membrane translocating property.

**Figure 6:**
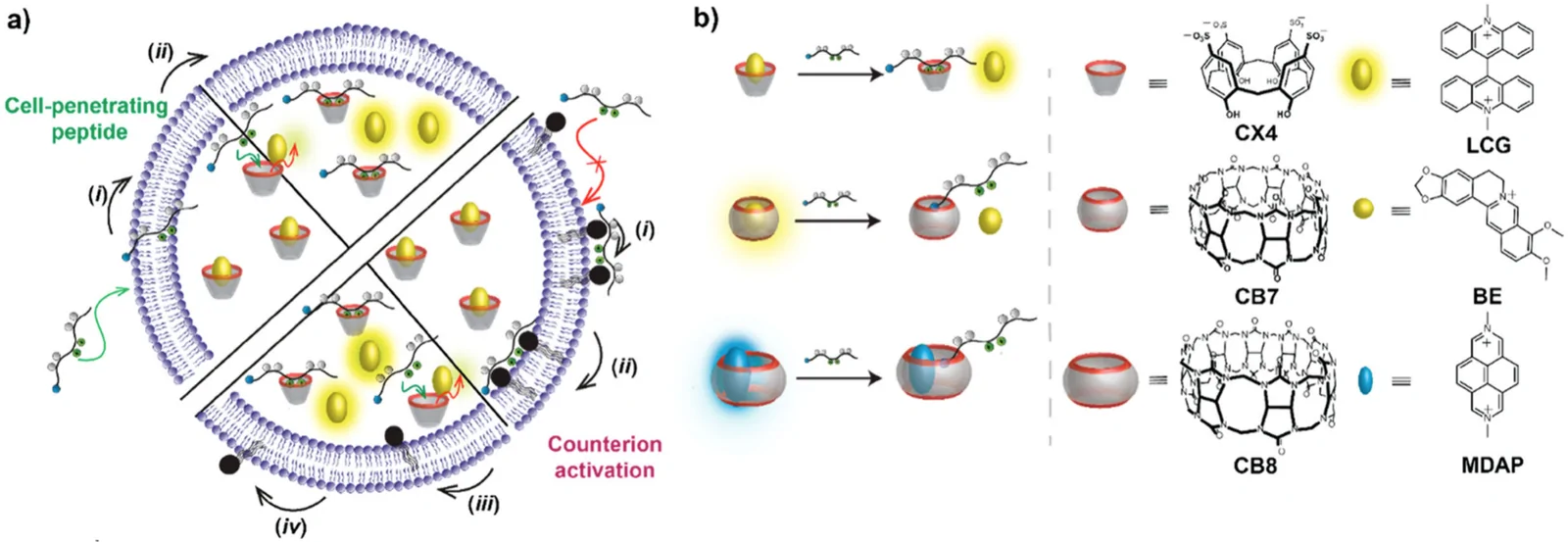
Liposome-based assay for translocation of unlabeled peptides. This assay uses supramolecular host–dye pairs to measure translocation of unmodified, dye-free peptides.^4^ (a) Several mechanisms of membrane translocation that result in exchange-dependent increase in fluorescence. (b) Schemes for fluorescence detection in supramolecular host–dye pairs, including relief of quenching or quenching, with displacement or no displacement. Some of the supramolecular hosts studied in this work are shown at the right. Adapted with permission from^4^. Copyright 2019 American Chemical Society.

#### 4.1.2 Giant Unilamellar Vesicle and Multilamellar Vesicle Assays

Giant unilamellar vesicles (GUVs) are μm-sized liposomes with a single outer membrane that can be made by several methods, including gentle hydration^169^ and electroformation.^170^ GUVs are large enough for individual vesicles to be imaged using confocal microscopy, enabling direct visualization of translocation. GUV experiments can be performed with soluble dye markers inside or outside the vesicle to simultaneously monitor permeabilization, and with lipid markers to monitor vesicle architecture and stability.^171^

For example, Almeida, Pokorny, and colleagues^13^ as well as Yamazaki and colleagues^172,173^ used confocal microscopy to study the translocation of transportan 10 and other MTPs into GUVs. Transportan 10 was shown to readily translocate into GUVs made from PC without anionic lipids,^13^ accompanied by a measurable influx of a charged dye molecule from the solution. The lack of changes in the overall architecture of the GUVs suggests that transportan 10 only moderately disrupts the membranes under these conditions. Almeida and colleagues noted that many GUVs contain small inner vesicles, Figure 7, and they used these inner vesicles to measure translocation of some peptides. This is a very useful approach when the peptide binds strongly to bilayers because, in that case, the free peptide in the vesicle lumen is not measurable. However, translocation followed by binding to the inner vesicles can be easily measured. Hennig and colleagues also used the macrocycle competition assay,^4^ described above, in GUVs, showing that similar results are obtained in GUVs and LUVs.

**Figure 7:**
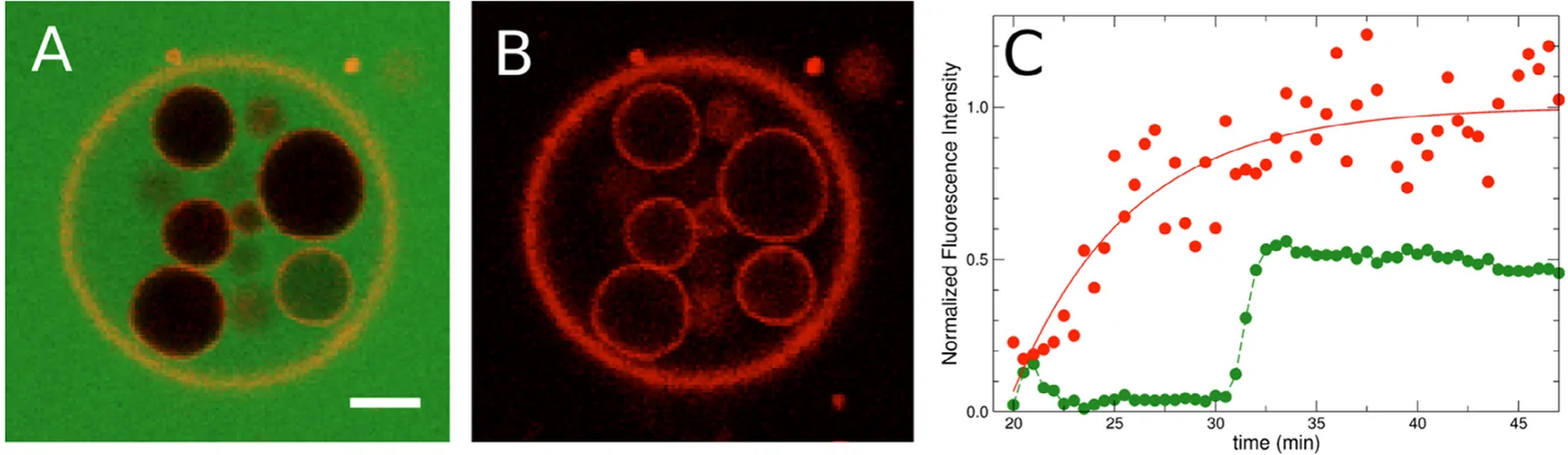
Peptide translocation into giant unilamellar vesicles, measured by confocal microscopy.^13^ (A,B) Image of a GUV with internal vesicles after peptide addition. The peptide is labeled with a red dye, and the green dye is a soluble marker. Note that the internal vesicles are labeled with peptide that has translocated. The aqueous dye has entered through the outer membrane, but not through most of the internal vesicles indicating moderate disruption of membrane integrity. (C) Time courses of translocation (red) and dye entry (green). Reproduced, in part, with permission from ref 13. Copyright 2013 American Chemical Society.

Instead of GUVs, it is also possible to use multilamellar vesicles (MLVs) in confocal microscopy assays of peptide translocation.^3,10,125^ MLVs are spontaneously produced when lipids are dispersed in aqueous solution.^174^ While they range widely in size, it is easy to isolate the large μm-sized vesicles that can be imaged with confocal microscopy as shown in Figure 8. We have used this approach to study membrane translocation of several families of peptides, including the SMTP family,^10,125^ which spontaneously translocate across pure phosphatidylcholine MLVs.^10,125^ An advantage of using MLVs instead of GUVs is that many more lipid compositions can be tested, and a greater range of peptide concentrations can be evaluated, because stable GUVs are not possible with all lipid compositions. MLVs also enable measurement of translocation of peptides that bind strongly to bilayers, because the internal bilayers provide a sink for peptides that have translocated.

**Figure 8:**
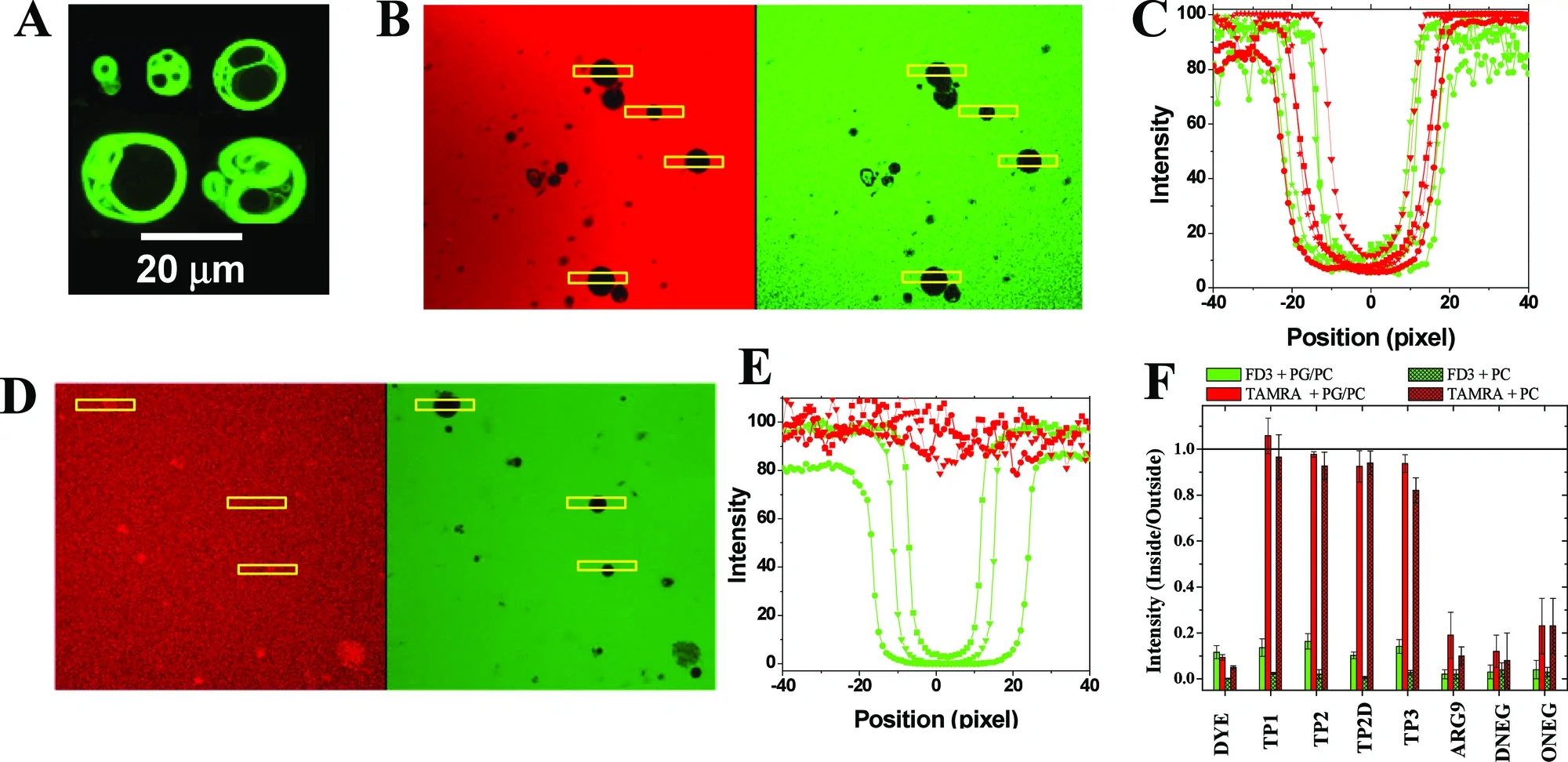
Peptide translocation into multilamellar vesicles measured by confocal microscopy. (A) Confocal images showing the architecture of multilamellar vesicles (MLVs) made from phosphatidylcholine lipids. In this panel only, lipids are labeled with green dye. (B) MLVs after incubation with free TAMRA dye (red) and dextran (green), which do not cross bilayers. (C) Intensity scans across MLVs incubated with control dyes demonstrates quantitation of external and internal intensities. (D) Confocal images of MLVs incubated with TAMRA labeled TP2, a spontaneous membrane translocating peptide (SMTP) that readily crosses into the vesicles, along with dextran (green) which marks the aqueous phase. (E) Intensity scans across MLVs incubated with TAMRA-TP2 (red) and dextran (green) shows that the peptide has equilibrated across the bilayers. (F) Confocal-based quantitation of translocation of SMTPs, nontranslocating control peptides, free TAMRA, and dextran in PC and PC/PG MLVs. Reproduced with permission from ref 10. Copyright 2011 American Chemical Society.

#### 4.1.3 Parallel Artificial Membrane Permeability Assay (PAMPA)

PAMPA is a fast *in vitro* membrane permeability assay that mimics the biophysics of the membrane.^175^ The PAMPA assay enables measurement of passive transport of molecules across multiple artificial membranes which can be made from many different lipid compositions.^176^ Although it was initially designed to measure the permeability of small drug molecules, it has also been used to study peptides. For example, Ano et al. assayed small uncharged peptides and compared PAMPA results to translocation in the Caco-2 cell line.^177^ Malakoutikhah et al. studied multiple peptides with modified amino acids^178^ and compared the membrane transport and retention of peptides using HPLC-MS for quantification of the transport. Different cyclic peptides were tested as well. Cis-cyclic peptides are a key feature for the intestinal artificial PAMPA membrane.^179^ Additionally, enantioselectivity was observed for some cyclic peptides.^180^ Fluorescent labeled peptide can be used to observe the transport,^181,182^ which simplifies detection. Ultimately, because PAMPA measures passive diffusion of peptides, classical cationic MTPs will not show transport using this technique, and so PAMPA can help distinguish mechanistic classes of peptides.

### 4.2 Eukaryotic Membrane Assays

Eukaryotic-cell-based assays are essential and relevant methods used to study MTPs. In contrast to synthetic membrane systems, eukaryotic cells present a highly complex and dynamic environment in which plasma membrane compositional complexity,^86,183^ membrane potential, endocytic pathways, cytoskeletal dynamics, and intracellular compartmentalization all influence MTP behavior. Many of these factors cannot be reproduced in synthetic membranes. Eukaryotic cells enable us to study the many ways in which MTPs engage native membranes and deliver functional cargoes to intracellular compartments. Described below are various techniques that are used to quantify MTP activity in cells to guide their optimization for therapeutic and translational applications and to interrogate cellular systems for information on the mechanistic landscape.^184,185^

#### 4.2.1 Visualization of MTP Activity by Fluorescence Microscopy

Microscopy techniques are frequently used to study peptides that cross cellular membranes. Laser scanning confocal microscopy is especially useful for tracking peptide and cargo localization within cells.^5,10,22,97,125,155,186–198^ A wide range of fluorescent dyes, proteins, and other probes have been employed to visualize MTP activity in both live and fixed cells using confocal microscopy. Synthetic fluorescent dyes are particularly useful due to their favorable optical properties.^195–197^ Commonly used fluorophores in eukaryotic cell assays include TAMRA, fluorescein, AlexaFluor, and cyanine dyes,^97,155,190–198^ which are readily conjugated to peptides, proteins, or other molecular targets. For example, TAMRA-labeled MTPs have been extensively used to study both the efficiency and mechanisms of peptide entry into cells.^5,10,125,199^ Fluorescent cargoes can also be employed to monitor MTP activity, including dye-labeled cargoes,^154^ fluorescent proteins,^200^ or chimeric cargoes that contain fluorescent proteins.^201,202^ These approaches enable direct visualization of both peptide uptake and cargo delivery. However, synthetic fluorescent dyes also present several limitations.^196,203^ Conjugation of dyes may alter peptide structure and activity, particularly due to electrostatic interactions since most MTPs are polycationic, while most visible fluorescent dyes are anionic. For instance, Hirose et al. demonstrated that conjugation of AlexaFluor488 to the arginine-rich peptide R12 induced accelerated aggregation or particle formation, a phenomenon not observed in unlabeled R12.^155^ Fluorophores may also fail to accurately report peptide localization if their signal is altered by cellular conditions, self-quenching, or interactions with intracellular molecules.^22,204^ Fluorescein, although commonly used, exhibits near-complete fluorescence loss at acidic pH, making it an unreliable probe for visualizing endocytosed peptides.^195,205^ In addition, synthetic dyes remain fluorescent even after peptide degradation,^199^ which can obscure interpretation of intracellular signals unless supported by chromatography or mass spectrometry.^199^

#### 4.2.2 Confocal Microscopy Experimental Design

To perform confocal microscopy experiments, adherent eukaryotic cells are typically grown to the desired confluency on treated coverslips. Cells are then incubated with fluorescently labeled peptides and/or cargoes prior to imaging. Although fixation has been used previously to study MTPs, observations made in fixed cells lack information about dynamic processes occurring at cellular and subcellular levels.^206^ Fixation with paraformaldehyde has the potential to cause experimental artifacts when imaging MTPs.^188,207^ For example, Richard et al.^189^ found that formaldehyde fixation dramatically influenced the distribution of the TAT peptide in HeLa cells, resulting in localization of peptide to the nucleus that is not consistent with live cell images. Similarly, Arg9, a polyarginine peptide, was also found to localize to the nucleus following fixation with formaldehyde; live cell images indicated cytoplasmic distribution prior to fixation.^189^ Arginine-rich peptides, especially, do not fix reliably.^207^ Consequently, live-cell imaging is generally preferred, as it is simpler, introduces fewer artifacts, and provides dynamic information on peptide behavior and cellular processes.^206^

Numerous dyes and probes are available to simultaneously monitor additional cellular processes without interference, facilitating investigation of the mechanistic and physical context of MTP activity *in vitro*.^5,61,63,161,188,189,207,208^ Automated image acquisition enables capture of events in live cells over time, yielding kinetic information and, depending on the probes used, insight into intracellular processes.^61,63,96,186^ For example, Macchi et al. monitored the uptake rate of the SMTP TM9 labeled with the ATTO 425 green fluorophore to determine diffusion kinetics and cellular internalization rates.^192^ Raw images can be analyzed using software such as ImageJ,^191,209,210^ converting parameters such as fluorescence intensity and diffusion into quantifiable metrics and significantly enhancing the analytical power of confocal microscopy experiments.

#### 4.2.3 Mechanistic Insights from Live-Cell Confocal Imaging

Live-cell confocal fluorescence microscopy provides extensive mechanistic and functional insight into MTP–cell interactions.^5,61,63,113,154,188,189,207,208,211^ Real-time visualization allows quantification of uptake rate, efficiency, and dose dependence at single-cell resolution, Figure 9. Subcellular localization analysis reveals whether MTPs accumulate at the plasma membrane, localize within endocytic vesicles, or distribute to intracellular compartments such as the cytosol, nucleus, mitochondria, or lysosomes, thereby informing uptake pathways and intracellular trafficking. Colocalization with organelle markers or endocytic tracers enables discrimination between direct translocation and endocytosis-mediated entry and facilitates identification of endosomal escape events.^160^ Time-lapse imaging further allows assessment of cytosolic delivery, intracellular retention, and redistribution over time, while simultaneous use of viability or membrane integrity probes reveals potential cytotoxic or membrane-perturbing effects.

**Figure 9:**
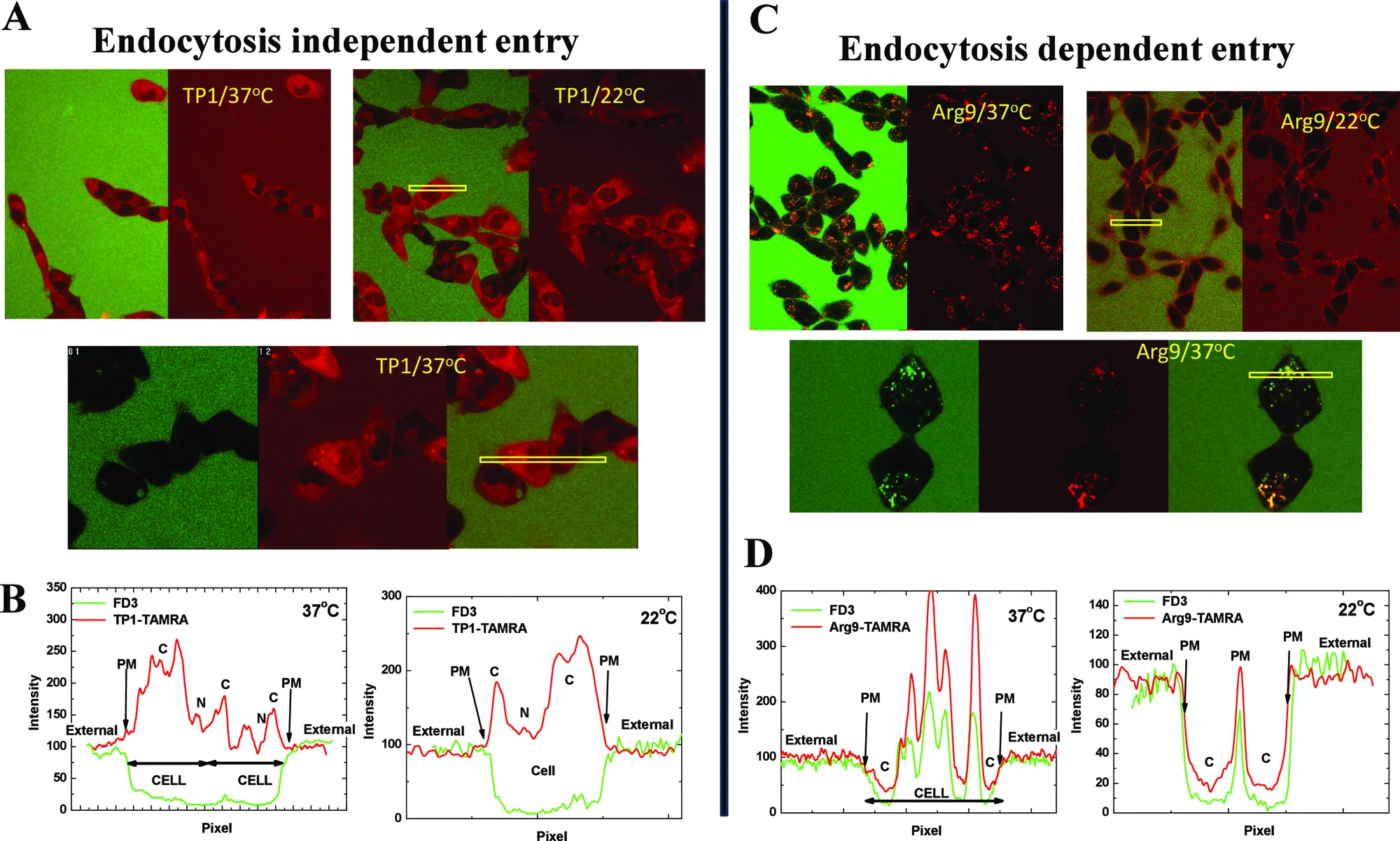
Laser scanning confocal fluorescence microscopy of two membrane-traversing peptides. The SMTP TP1 (left) and the CPP Arg9 (right) labeled with TAMRA (red) were incubated with Chinese hamster ovary (CHO) cells at low concentration, ≤1 μM. Dextran (green) is added to mark the external spaces. Measurements were made at 37 °C where endocytosis occurs normally and at 22 °C, where it is inhibited. (A) The spontaneous membrane translocating peptide TP1 enters the cell cytosol readily at both temperatures. (B) Quantitation of external and internal concentrations of TP1 and dextran in the regions of the yellow rectangles in (A). (C) The cationic membrane traversing peptide Arg9 is endocytosed at 37 °C but remains entrapped in the endosomes. At 22 °C, Arg9 remains bound to the cell surface but is not internalized and does not translocate. At higher concentrations, Arg9 enters cells by direct plasma membrane translocation (not shown). (D) Quantitation of external and internal concentrations of Arg9 and dextran in the regions of the yellow rectangles in (C). Reproduced with permission from ref 10. Copyright 2011 American Chemical Society.

A key feature often examined in MTP studies is the ability of peptides to bind to and self-assemble into clusters at cellular membranes,^61,63,113,155,212^ which sometimes contain lipids, cargo, and media.^61^ Confocal microscopy enables direct visualization of these interactions. When MTPs are labeled with spectrally distinct fluorophores, peptide–membrane association and surface aggregation can be identified through spatial overlap of fluorescence signals.^113,154,211^ Fluorescently labeled cargo molecules can be tracked concurrently, providing insight into whether membrane association correlates with productive intracellular delivery.^213–216^

Application of membrane-impermeant dyes to the extracellular medium further enhances contrast between intracellular and extracellular spaces, enabling discrimination of true internalization events and real-time assessment of membrane integrity.^217^ Propidium iodide is commonly used for this purpose as an indicator of membrane permeabilization and cell viability.^217^ In addition, specific lipid components can be selectively probed to investigate peptide–lipid interactions during translocation. For example, phosphatidylserine can be labeled using annexin-based probes to assess whether MTPs preferentially interact with or perturb this lipid during cellular entry.^218^ Laurdan (6-dodecanoyl-2-dimethylaminonaphthalene), a green fluorescent lipophilic probe sensitive to membrane polarity and hydration, is frequently used to monitor lipid order and peptide-induced membrane perturbations.^219,220^

Confocal microscopy also allows independent tracking of multiple experimental variables including both the MTP and its delivered cargo. Fluorescently labeled peptides can be followed in the cell, while delivery efficiency can be assessed using fluorescent cargoes such as GFP or GFP-fusion proteins, which provide a stringent readout of cytosolic delivery and endosomal escape.^21,61–63,96,156,158,159^ In addition, enzymatic protein cargoes,^221^ dye-labeled protein cargoes, and fluorescent protein chimeras^161,222–224^ have been successfully tracked to evaluate functional delivery and intracellular activity.

#### 4.2.4 Cell-Survival and Apoptosis Assays

Biochemical assays^2,225–228^ for cargo delivery can be especially useful in assessing MTP activity because they can probe for the biological activity of cargoes such as bioactive proteins or peptides. Multiple assays have been developed to test the cytosolic access of various peptides or to quantify uptake efficiency. These assays can be categorized by the reporters used.^2,21,160^

Peptide-enabled delivery of cytotoxic or proapoptotic cargoes can easily be measured using one of many cell survival or apoptosis assays, which report on factors such as mitochondrial reductase activity, membrane integrity, cell count, caspase activity, and others. For example, the 14-residue amphipathic peptide “KLA” ^229–231^ damages mitochondrial membranes and subsequently triggers apoptosis via caspase triggered cascades,^232,233^ but only if delivered to the cytosol. Thus, delivery of KLA can be measured by apoptosis assays.^6,229–231,234^ Similarly, delivery of BH3-only peptides, which are α-helical fragments of pro-apoptotic proteins designed to bind and inhibit antiapoptotic BCL-2 family members, can be measured using these assays. Much research has focused on stapled variants of these peptides that have higher stability, higher affinity, and better resistance to degradation than linear peptides.^235,236^ The cyclic heptapeptide mushroom toxins α-amanitin and phalloidin have also been used to assess peptide-mediated delivery by measuring the response of cells with apoptosis, viability, or cell mobility assays.^127,237–240^ Protein cytotoxins can also be used to assess delivery; Wu et al. assessed productive endosomal escape^191^ of a catalytically active protein cargo PE-III, the truncated third domain of the *Pseudomonas* exotoxin A.^241,242^ PE-III is an ADP-ribosyltransferase that inactivates elongation factor-2 (eEF-2) and triggers apoptosis by blocking protein synthesis. It is highly toxic; just one or a few molecules of cytosolic PE-III may be lethal to cells.^242^ However, PE-III lacks the native cell binding and transduction domains and is inactive unless delivered exogenously to the cytosol. Wu et al. incubated 40 nM PE-III with cells along with serially diluted pH-responsive, acylated pHD peptide nanopore formers and measured cell viability using Alamar Blue. The loss of viability caused by PE-III or similar enzymes (e.g., cholera toxin A subunit) is an extremely sensitive functional indicator that protein cargo has been delivered, intact, to the cytosol, but it cannot give a quantitative measure of how many molecules were delivered because one enzyme inactivates many elongation factor molecules. Instead, Wu et al. quantitatively compared relative delivery efficiencies for different peptides by fixing PE-III concentration at 40 nM and measuring the midpoint concentration of peptide required for 50% activity.

#### 4.2.5 SLEEQ and SEE/Split-GFP Assays

Luminescence is a sensitive, useful measure for delivery efficiency. For example, Teo et al. created the SLEEQ assay (Figure 10), a split-NanoLuc complementation assay developed specifically to quantify the fraction of protein that escapes endosomes and reaches the cytosol.^156^ The assay uses cargo proteins which are tagged with the small HiBiT peptide (reporter from Dixon et al.^243–246^ ). Cells are modified to express the complementary LgBiT protein actin to restrict the reporter to the cytosol. Only the HiBiT-GFP complexes that escape endosomes and reach the cytosol can attach to LgBiT and then produce a bright luminescent signal once a substrate, fumarizine, is added. To calculate the escape efficiency, SLEEQ measures luminescence before and after adding digitonin to permeabilize the membranes and allow HiBiT, trapped in compartments, to access LgBiT. The pre:post-digitonin luminescence ratio is used to obtain an “endosomal-escape efficiency”. Teo et al. showed a linear detection down to picomolar HiBiT (5 pM). This ultrasensitivity is relative to a similar assay upon which it was based, a split-GFP assay (Figure 10a) developed by Milech et al.^224^ The split-GFP assay and split-complementation endosomal-escape (SEE) assays use fragments of GFP11/GFP1–10, bound to an MTP–cargo complex, then use complementation to detect cytosolic access.^5,161,224,247–250^ The two assays are conceptually similar, as SLEEQ was developed as a continuation of SEE. In both assays, complementation only occurs in the cytosol, but SEE has a lower sensitivity and requires micromolar cargo concentrations to produce measurable signal. SEE/split-GFP approaches are useful for visual confirmation and for cargoes that can bind to the GFP11 tags, but they can miss detection of low-level escape that SLEEQ detects.

**Figure 10:**
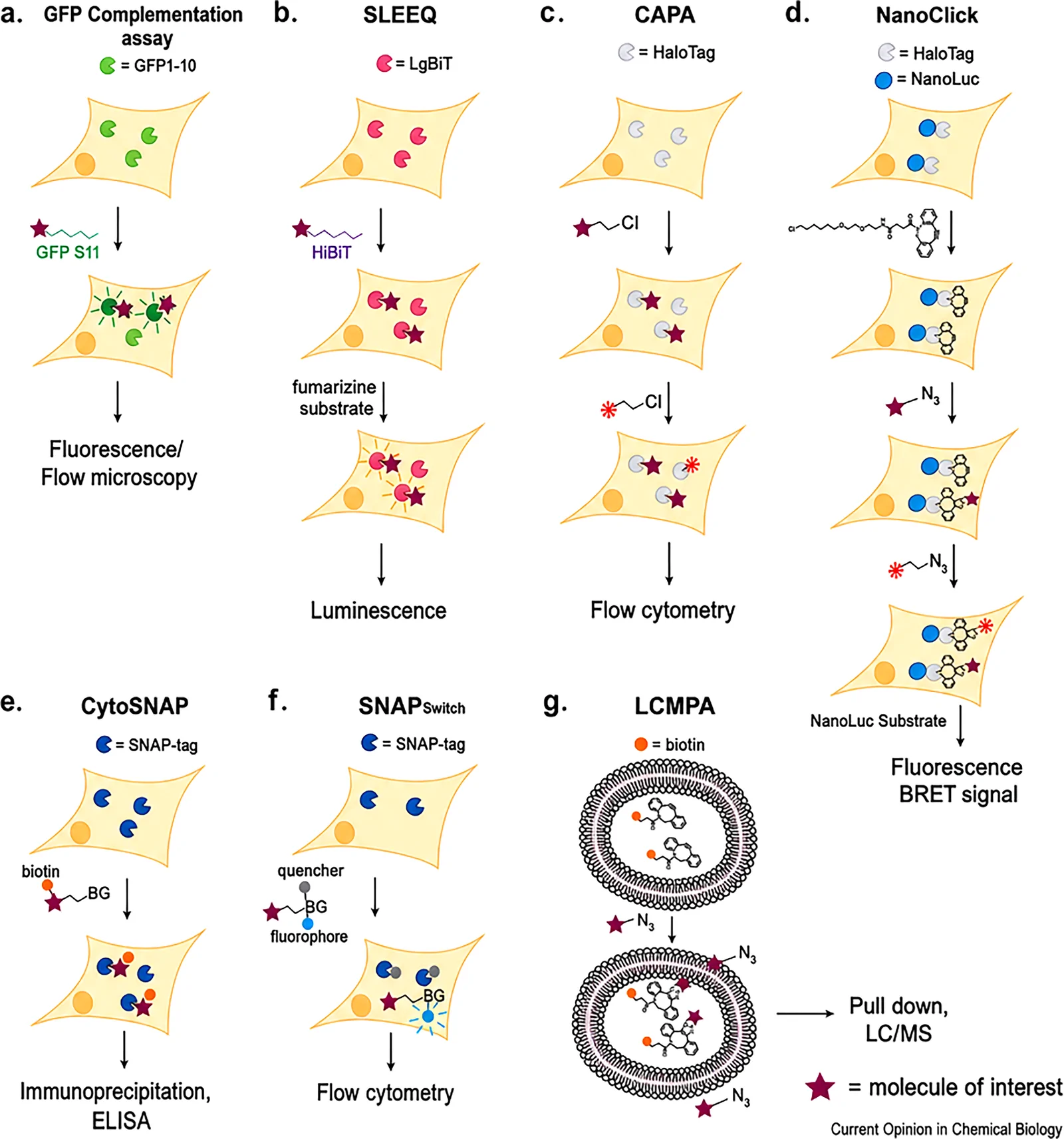
A simplified step-by-step illustration of tag-based cytosolic delivery assays as reviewed by Batistatou and Kritzer.^2,3^ Each panel details the assay title, the reporter and its localization, the intracellular reporter complex containing the molecule of interest, any additional substrates, and the method of readout. Reproduced with permission from ref 2. Copyright 2024 Elsevier.

#### 4.2.6 Luciferase Splice-Correction Assay

Kang et al. developed a reporter assay for oligonucleotide delivery^251^ by stably transfecting HeLa cells with a recombinant plasmid (pLuc/705) carrying the luciferase gene interrupted by a mutated human β-globin intron 2 (IVS2-705). The β-globin mutation causes aberrant splicing of luciferase pre-mRNA, preventing translation of functional protein. When a complementary molecule binds to the pre-mRNA at the mutated splice site, it blocks that default splice site, and restores a cryptic splice site, leading to the production of many luciferase molecules. This system has been used to study peptide-mediated delivery of RNA,^252^ peptide-nucleic acids (PNA),^5,253,254^ and phosphorodiamidate morpholino oligomer (PMOs).^255^ For example, Kauffman et al.^5^ used this system to screen a hybrid library of peptide-PNA705 chimeras. They incubated cells briefly with peptide-PNA conjugates, allowed them to recover for 24 h, and then measured luciferase activity. In this screen, they discovered that PNA delivery peptides (PDEPs) delivered PNA705 more efficiently and at lower concentrations than the classical CPPs, TAT and penetratin.^5^ While this assay is very sensitive, its major drawback is that a single corrected mRNA can translate into many luciferase enzymes, meaning that the assay is amplified and cannot report the absolute number or fraction of molecules that escape endosomes. The assay reports a binary end result (splice correction) and potency (dose response/EC_5_0). Aside from that result, approaches such as comparison of L- and D-peptides, dye-labeling and confocal microscopy, or chloroquine cotreatment can be used to probe the extent to which delivery is limited by endosomal trapping.

#### 4.2.7 GIGI and GIGT Assays

Holub et al.^256^ adapted and improved a glucocorticoid-based reporter assay first developed by the Kodadek lab to create two complementary assays.^257^ In GIGI (Glucocorticoid-Induced eGFP Induction), cells express a Gal4-GR★-VP16 artificial transcription factor plus a Gal4-driven eGFP reporter. A dexamethasone (Dex)-tagged cargo that reaches the cytosol binds GR★, releasing its nuclear localization signal and driving eGFP transcription.^258^ After an incubation period, eGFP is measured in lysates, or by microscopy or flow cytometry. Transcription and translation amplify the signal, making GIGI very sensitive to low levels of cytosolic delivery, but it is relatively slow (6–24 h) and reports a relative amount of cytosolic ligand rather than an absolute molecule count. GIGT (glucocorticoid-induced eGFP translocation) instead uses a GR★-GFP fusion, which when exposed to cytosolic Dex, translocates from the cytosol to the nucleus and can be measured by live-cell imaging and image analysis (nuclear/cytoplasmic ratio). GIGT is fast relative to GIGI (minutes), but the signal is not amplified, which limits sensitivity. Originally, Kodadek and colleagues used a wild-type GR and a luciferase reporter gene. Holub et al. instead used a higher-affinity GR★ variant to increase assay sensitivity and reported the possibility for screening.^259^ Both assays require the cargo to be Dex-conjugated or in some way able to bind GR.

#### 4.2.8 Cre Recombinase Assays

Cre-based cytosolic delivery assays use genetically encoded loxP-STOP-loxP reporter cassettes to produce an amplified readout; this assay provides a binary result of whether Cre recombinase reaches the cytosol and moves into the nucleus. Wadia et al. treated cells encoding loxP reporters (loxP-STOP-loxP-EGFP and -LacZ) with a CPP TAT-Cre fusion in an assay to determine cytosolic and nuclear delivery.^260^ The reporter activates only when Cre can escape endosomes, enter the cytosol, and reach the nucleus without degradation to excise the STOP codon. Readout is by flow cytometry or β-gal staining, 18–24 h after treatment. In this process, one recombination event results in a stable, amplified, and irreversible reporter activation; because of this the assays are extremely sensitive to successful delivery, but, like other assays discussed here, they do not provide an absolute molecule count or a percent-escaped measurement. From this work came a variety of Cre-loxP stoplight reporters that have adopted this method to measure Cre-CPP delivery; for example, Schneider et al.^131,261–263^

#### 4.2.9 ChloroAlkane 9 CAPA/HaloTag—Chloroalkane Penetration Assay

The penetration assay or CAPA (Figure 10c), as coined by Peraro et al.,^264,265^ uses the HaloTag enzyme^266,267^ as a binding site to report cytosolic localization of chloroalkane (ct)-tagged molecules. In this assay, cells are modified to stably express HaloTag in the cytosol. The cells are then pulsed with chloroalkane-tagged peptides, washed, then chased with a fluorescent ct-dye. The ct-cargo that escapes endosomes will bind to the HaloTag and block the enzyme. Any HaloTag molecules that were irreversibly bound to ct-cargo during the pulse are unavailable to be labeled by the chase dye. To quantify this procedure, they used flow cytometry to measure the fluorescent-dye signal per cell, and the inverse signal corresponds to the fraction of HaloTag blocked by ct-cargo. CAPA is compartment-specific (by changing HaloTag localization) and reports productive access to that compartment; it reports only the cargo that can reach and react with the HaloTag, meaning it can be used to quantify endosomal escape. CAPA succeeds in giving quantitative nonamplified dose–response readouts and the ability to compare structure–penetration relationships. There are limitations, however; CAPA requires a chemically conjugated chloroalkane tag on cargos, it only measures overall cytosolic delivery and not real-time kinetics of escape, and it does not provide an absolute measurement of cargo per endosome.^268^

#### 4.2.10 NanoClick Assay

The NanoClick assay (Figure 10d) developed by Peier et al.^212^ uses in-cell copper-free “click” chemistry with a NanoBRET readout.^269,270^ NanoClick builds upon Promega’s NanoLuc-HaloTag (NanoBRET) platform.^271^ This system pairs a genetically modified NanoLuciferase with the HaloTag; the fusion is then covalently labeled with chloroalkane ligands. When a fluorophore is bound to this ligand, it produces the BRET signal. NanoClick repurposes this system by expressing the fusion protein in the cytosol of HeLa cells. The fusion is then loaded with dibenzoazacyclooctyne–chloroalkane (DIBAC–CA). The peptide of interest is modified with a small azide and only those peptides that reach the cytosol proceed to strain-promoted alkyne–azide cycloadditions (SPAAC) which irreversibly block the DIBAC. Unreacted DIBAC is labeled with an azide-NanoBRET acceptor dye (NB618-Az). Once the complex is complete, the NanoLuc substrate is added and the complexes bearing dye produce the BRET signal, which is inversely proportional to the amount of cytosolic azide-peptide. The NanoClick assay can target different compartments and can be adapted for high throughput screening. As the signal is based on the covalent capture of the complexes, this assay can be used to quantify cytosolic exposure, but cannot provide an absolute count of the peptide molecules.

**Figure 11:**
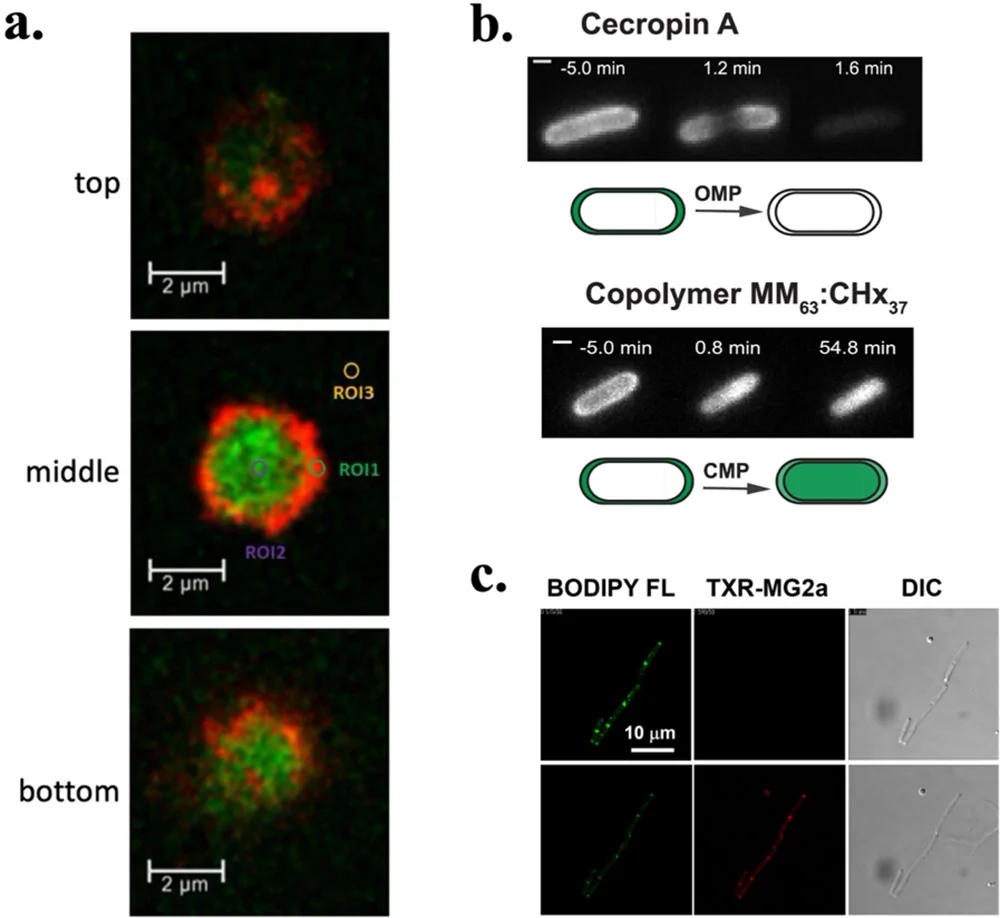
Fluorescence microscopy visualizes peptide translocation into live bacterial cells. (a) Confocal microscopy images of a peptide-treated *E. coli* spheroplast. Each image is a slice through the *z*-axis of the cell. Peptide was labeled with FITC (green), and the cell membrane was labeled with di-8-ANEPPS (red). (b) Single-cell, time-lapse confocal microscopy images of an *E. coli* cell expressing periplasmic GFP. Following treatment with either Cecropin A or Copolymer MM_63_:CHx_37_, changes in distribution of GFP signal outer membrane permeabilization (OMP) or inner membrane permeabilization (CMP). (c) In-cell FRET images of BODIPY FL DLPE-labeled *B. megaterium* (green) before (top row) and after (bottom row) treatment with TXR-labeled magainin peptide (red). Adapted with permission from ref 450. Copyright 2024 American Chemical Society. Adapted with permission from ref 370. Copyright 2018 American Chemical Society. Adapted with permission from ref 316. Copyright 2021 American Chemical Society.

#### 4.2.11 SNAP-tag Based Assays

Self-labeling protein tags, similar to the HaloTag, are being used in other *in vivo* assays. An example of this is the SNAP-tag, which is an hAGT variant that binds to O^6^ benzylguanine.^272–274^ FitzGerald et al. used this to introduce the SNAP-switch assay (Figure 10f).^275^ The assay uses a benzylguanine (BG) derivative bound to a quencher and a fluorophore. The substrate is designed to be attached to the molecule of interest through click-chemistry and will fluoresce only when it enters the cell and encounters a SNAP-tag localized to a cellular compartment. The quencher will transfer to the SNAP-tag when encountered, and the fluorophore will remain attached to the molecule of interest, irreversibly fluorescing after the reaction. The now permanent fluorophore can be read by flow cytometry or fluorescence imaging. The SNAP-switch assay reports localization and is pH-insensitive, although it is not an absolute count; this is due to the signal accumulating from the irreversible reaction with the SNAP-tag. Lucchino et al. used this same principle to develop the Cyto-SNAP assay (Figure 10e), which measures the absolute number of molecules that reach the cytosol.^157^ Their method modifies the molecule of interest with a BG group and a biotin (BG–biotin or maleimide-BG-biotin). The cells are once again engineered to express the SNAP-tag; this assay expresses the tag in the cytosol, bound to a fluorescent protein, mNeonGreen.^276^ This method was designed so that only the cargo that reaches the cytosol, while bearing the BG, can react covalently with the mNeonGreen-SNAP complex. After incubation for up to 4 h, the reaction takes place, and the fusion can be immunoprecipitated by anti-mNeonGreen nanobody beads and detected by ELISA. Lucchino et al. also show that the nonreacted SNAP complexes can be blocked and then released from cells, permitting production of an absolute count of the cytosolic molecules. The Cyto-SNAP assay is linear and sensitive to the picomolar range. Practical limitations to this method are due to the required chemical labeling of the cargo with BG and biotin, as well as the expression of the large, hydrophobic SNAP-tag in the cell line.

#### 4.2.12 Transwell Assays

MTPs have been explored as delivery vehicles that facilitate transport across endothelial and epithelial layers.^199,277–280^ In such applications, it has been of particular interest to deliver therapeutics across the blood–brain barrier (BBB), which is created by brain microvascular endothelial cells.^281,282^ These cells express active efflux pumps and interact via tight junctions to form defect-free monolayers that impose tight restrictions on the passage of molecules, especially macromolecules.

Since many MTPs can be taken up effectively and rapidly into cells, it has been hypothesized that MTPs may also bridge endothelial and epithelial layers by readily crossing both the apical and basolateral membranes of cells. Imaging-based approaches have been used to measure the transcellular movement of peptides.^283^ However, the experimental tool of choice for probing such activities is the transwell assay.^199,277,278,280^ The transwell experimental setup consists of two aqueous compartments separated by a monolayer of cells grown on a porous solid support. Transwell inserts are commercially available and can be used in conjunction with ultralow attachment 24-well plates. Often, MDCK cells are used as the cells of choice in these experiments, as they are considered a good model of the BBB.^284^ Caco-2 cells are frequently used as a model of the intestinal barrier.^285^ Molecules of interest, such as MTPs, are added to the apical chamber, and their concentration on the basolateral side is assessed after hours to days. Peptides or cargoes on the trans side of the cell monolayer can be assayed by fluorescence, HPLC, or mass spectrometry.^199^ Such permeability experiments should be accompanied by measurements of trans-endothelial electrical resistance (TEER), which is high for defect-free monolayers but decreases as the monolayer integrity is compromised.^286^ Along with TEER measurements, researchers measure the permeability of Lucifer yellow across the barrier cell layer, a known negative permeability control. High TEER values and low Lucifer yellow permeabilities indicate defect-free cell monolayers, such that any transport of tested MTPs across the barrier must occur through the barrier cells.^286^

As an example, this approach has been used to study an MTP called “CL”, which contains an amphipathic, helix-like motif sequence (RLLRLLR) and a polyarginine tail. Komin et al.^199^ explored many variations in the helix-like motif and the polyarginine tail. They also compared CL variants to other well-known MTPs. The CL peptide comprised of the RLLRLLR motif and an Arg_7_ tail efficiently delivers fluorescent dyes across MDCK cell monolayers^199^ at a high rate. These authors show that several well-known MTPs do not translocate across cell monolayers, consistent with other reports that cell penetrating peptides exhibit low permeabilities across endothelial layers.^287,288^ The CL peptide has a complex mechanism where the intact peptide rapidly crosses into the MDCK cells from the apical side, whereupon the dye cargo is proteolytically released intracellularly, and then subsequently crosses the basolateral membrane. Addition of the cleavage product to the apical side does not result in significant transport across the monolayer.

Results from transwell experiments with MDCK cells can be compared to results from animal brain–perfusion studies.^289–291^ Some assays of peptide penetration into brains of mice involve homogenizing extracted brain tissue, wherein the brain capillaries are separated from the parenchyma by centrifugation. The SynB family of membrane traversing peptides, especially SynB-1 and its truncated variant SynB-3, have shown promising potential for enhancing drug delivery across the BBB.^289–291^ *In situ* brain perfusion studies demonstrate that conjugating drugs such as doxorubicin and paclitaxel to SynB peptides increased brain uptake, likely because the peptide–drug conjugates avoid P-glycoprotein efflux mechanisms that normally limit the brain permeability of free drugs.^289^ However, while SynB-3 increased total brain-associated paclitaxel uptake and significantly improved its solubility, its actual delivery into the brain parenchyma is uncertain. One study showed that the neuropeptide dalargin had significantly increased analgesic activity when conjugated to the SynB MTPs, demonstrating effective crossing of the BBB.^292^ Mechanistic studies indicate that SynB peptides likely cross the BBB through adsorption and transcytosis, active processes driven by interactions between positively charged peptides and negatively charged luminal surface molecules on brain capillaries. Supporting evidence includes saturable uptake, competition with other cationic molecules, and a lack of stereoselectivity in transport. Similarly, Jackman et al. showed that an amphipathic antiviral peptide called “AH” has antiviral activity in the brain of mice infected with enveloped viruses when the peptide is injected systemically, indicating that it crosses the BBB.^293^

To overcome the limitations of measuring uptake in whole brain tissue, there is interest in developing more physiologically relevant alternatives to the transwell assay, such as tissue-engineered microvessel models.^284,294^ In one example, a cylindrical channel is created inside a collagen gel and seeded with MDCK cells to mimic a patent and perfusable lumen of a blood vessel.^284^ This system is biologically relevant because of the cylindrical geometry and continuous fluid flow in the lumen, which mimic the hydrodynamic and shear stresses acting on the cells. MTP-labeled fluorophores can be introduced into the flow, and their permeability through the cell layer can be quantified by measuring the increase in the fluorescence intensity in the collagen matrix surrounding the channel over time. The quality of each engineered microvessel can be determined by first perfusing Lucifer yellow as a control for barrier integrity.^284^ With this technology, the permeability of the CL peptide was measured as 3 × 10^–6^ cm s^–1^ in both transwell and microvessel assays.^199^

### 4.3 Prokaryotic Membrane Assays

The goal of most antimicrobial peptide (AMP) therapies developed against bacteria is to kill the cell, which is generally dependent on membrane permeabilization or cytosolic entry of cationic peptides.^295,296^ Since AMPs must either act on membranes or pass through membranes to kill bacterial cells, growth, inhibition, and sterilization assays are common indirect measures of possible membrane translocation.^297^ Permeabilization of bacterial membranes can be assessed by measuring changes in membrane potential,^298–305^ leakage of intracellular ions,^305^ efflux or influx of reporters,^300,306–312^ or binding of dyes that indicate cell viability.^304,313–316^ Release of endogenous proteins can also be measured by SDS-PAGE or biochemical assays.^140^ Additionally, bacteria can be engineered to express cytosolic fluorescent proteins that can be released and detected upon peptide addition.^140,317^

However, some classes of AMPs can translocate across bacterial membranes without permeabilization.^318^ In the case of Gram-negative bacteria, these peptides can cross the outer membrane (OM), travel across the periplasm, and then cross the inner membrane (IM) to enter the cytosol, all without causing membrane disruption or cell lysis. Instead, these AMPs act intracellularly to inhibit cell growth or induce cell death.^319–321^ Proline-rich antimicrobial peptides, such as Bac7 and apidaecin, are especially notable for having intracellular targets,^322–325^ including DnaK, a critical prokaryotic chaperone.^321,326–329^ Proline-rich AMPs have also been shown to inhibit bacterial protein synthesis by interacting with ribosomes.^330–333^ Other AMPs target bacterial nucleic acids to halt cell growth. For example, indolicidin, a tryptophan/proline-rich AMP, translocates into cells and binds DNA, inhibiting DNA synthesis.^334^ Buforin II, a histone-derived AMP, induces cell death by binding to bacterial DNA and RNA.^335^ Furthermore, some researchers have used MTPs to deliver antisense PNAs to the cytosol of the intracellular bacterium, *Listeria monocytogenes*.^336,337^ These antisense PNAs are synthetic nucleic acid mimics that form complementary complexes with bacterial DNA or RNA, blocking transcription or translation of essential genes. In the sections below, we describe assays that have been developed to probe the translocation of peptides and cargo across bacterial membranes.^155^

#### 4.3.1 Flow Cytometry

The most straightforward way of tracking peptide translocation into bacterial cells is by directly labeling the peptide with a fluorescent probe. Internalized peptide can then be detected in a flow cytometer, where cells pass through a laser one by one, allowing fluorescent signal from each cell to be captured.^338^ The advantage of flow cytometry is its ability to evaluate single cells in a high throughput manner. However, flow cytometry cannot resolve the subcellular location of the peptide, and it cannot distinguish between internalized peptide and membrane-bound peptide. Because of the latter, it is imperative to thoroughly wash the bacterial cells after peptide uptake to remove any peptides bound to the cell surface. Researchers have also utilized the addition of trypan blue to quench any extracellular fluorescent signal originating from surface-bound peptide.^339,340^

#### 4.3.2 Fluorescence Microscopy

The most widely used technique to visualize the translocation of a peptide into a bacterial cell is fluorescence microscopy. This method involves incubating bacteria with a fluorescently labeled peptide and then imaging the cells on a fluorescent microscope (Figure 11). This allows for the single-cell visualization of peptide–membrane interactions, peptide entry, and subcellular distribution of the peptide.^341^ However, the limited optical resolution of light microscopy makes fluorescent imaging of bacteria, which are much smaller than eukaryotic cells, significantly more challenging. With such limited resolution, one widefield image could contain both the cytoplasm and parts of the surrounding bacterial membrane, making it difficult to distinguish between peptide bound on the surface of the cell and peptide internalized in the periplasm or cytosol.^341^ Confocal fluorescence microscopy is commonly used to increase resolution and reduce ambiguity by imaging narrow cross sections of the cell to enable pseudo three-dimensional “z-stacking”, Figure 11. Confocal fluorescence microscopy has demonstrated the translocation of peptides, such as buforin II, across bacterial membranes in high resolution.^342^

An alternative solution for the limited resolution of fluorescence microscopy is to utilize lipid vesicles or modified bacterial cells that are larger and more spherical than native bacteria, making the membrane and cytosol easier to resolve. Researchers have modified both Gram-negative and Gram-positive bacteria into spheroplasts and protoplasts for peptide translocation studies using phase contrast and fluorescence microscopy, Figure 11.^343–347^ It is important to note that these modified bacterial cells have an altered physiology that may affect how peptides interact with them; however, they are closer representations of native bacteria than synthetic lipid vesicles.

In addition to labeling the peptide itself, the use of other fluorescent dyes and probes can provide further context for the activity and location of MTPs. For example, fluorescently labeling the cell membrane with membrane-staining dyes, such as di-8-ANEPPS or FM4-64, allows for the observation of peptide-induced changes in membrane morphology, the visualization of membrane-peptide colocalization, and the validation of true peptide translocation into the cell, Figure 11.^344,345,347,348^ Other fluorescent dyes, such as laurdan, can report on membrane fluidity, which can help elucidate the nature of peptide-membrane interactions.^349^ Addition of a fluorescent DNA-binding dye, such as propidium iodide or DAPI, can provide further locational context and reveal the intracellular substrates of nucleic acid-targeting peptides through colocalization observations.^350^ Probes that can cross intact membranes and exclusively fluoresce in the intracellular space, such as calcein-AM, can also be used to demonstrate peptide translocation across the membrane and localization in the cytosol without gross membrane permeabilization.^351^ Fluorescent fusion proteins (e.g., GFP) can be used to detect other components of the bacterial cell (such as the cell division machinery),^352^ enabling the examination of many different cellular processes affected by the internalization of peptide.

To increase the time resolution of fluorescence imaging, researchers have immobilized bacteria in agarose first and then added peptide, allowing for time-lapse imaging of peptide penetration. The Weisshaar lab^307,353–367^ utilizes custom microfluidic chambers, wherein media containing labeled peptide is applied to immobilized live bacteria such that fluorescent images can be taken every few seconds, elucidating all parts of the peptide penetration process. Using bacteria that express periplasmic GFP, they have been able to observe the sequence of individual events, including OM permeabilization, IM permeabilization, peptide entry into the cytosol, and peptide subcellular localization, Figure 11b. With the addition of other probes, they have measured other readouts, such as peptide-induced oxidative stress.^357,360^ By combining these methods with super-resolution microscopy and single-particle tracking, the Weisshaar lab has even been able to deduce peptide interactions in the cytosol after IM penetration.^363^

#### 4.3.3 FRET

Fluorescence resonance energy transfer (FRET) is an assay used to study molecular interactions and binding events in real-time. In FRET, two molecules (a “donor” and an “acceptor”) are labeled with different fluorophores. The donor fluorophore is excited, and if the donor molecule and the acceptor molecule are close enough in proximity, then the donor fluorophore can transfer energy to the acceptor fluorophore, which will then emit a signal that can be detected under a fluorescent microscope. Kaji et al. designed an in-cell FRET assay using BODIPY-labeled bacteria as the donor, and Texas Red-labeled magainin peptide as the acceptor.^316^ Notably, they used the bacterium *Bacillus megaterium* as the model organism, because it lacks an OM and is large in size, making measurements simpler and microscopy more practical. They found that following the addition of acceptor-labeled peptide, the donor fluorophore was partially quenched due to the energy transfer, indicating a close interaction between the peptide and the bacterial cell membrane (Figure 11c). It is notable that while FRET studies confirm interaction of the peptide with the cell membrane, they do not directly prove translocation of the peptide into the cell. However, Kaji et al. were able to observe the presence of labeled magainin peptide on both the bacterial membrane and in the cytosol while performing fluorescent imaging of the FRET experiments.

#### 4.3.4 Transmission Electron Microscopy

Because of the small size of prokaryotes compared to eukaryotes, electron microscopy is a particularly powerful tool for imaging bacteria because of its increased resolution compared to light microscopy. In transmission electron microscopy (TEM), a beam of electrons is passed through a thin specimen, allowing for high-resolution imaging of internal structures. To evaluate peptide uptake, TEM can be used to observe changes in the morphology of the bacterial cells following peptide treatment.^298,304,310^ Such observations may suggest peptide-mediated membrane permeabilization or cell death, but they are not indicators of peptide entry into cells. To directly track peptide translocation into cells using electron microscopy, the peptide can be conjugated to nanogold particles, prior to incubation with bacteria and TEM imaging.^317^ Nanogold particles appear as electron-dense dark spots on TEM images, revealing the subcellular location of the peptide (e.g., OM, IM, periplasm, or cytosol). Alternatively, ruthenocene-substituted derivatives of the peptide can be synthesized and used for TEM imaging since the ruthenium label appears electron-dense, allowing for the visualization of peptide accumulation in the cell.^304^

It is important to note that in all of the assays discussed thus far for measuring peptide translocation into bacterial cells, the peptide must be modified, usually by the addition of a fluorescent probe. These modifications may alter the physical and chemical properties of the peptide, which may affect how the peptide interacts with or crosses membranes.^155,368^ Additional assays should be performed to compare the dye-labeled and unlabeled peptides, such as membrane permeabilization assays, structural and biophysical characterization,^369^ or the assays described in the following sections.

#### 4.3.5 HaloTag Assay

As described previously, the Weisshaar lab developed single-cell, time-resolved fluorescence microscopy assays that could reveal membrane permeabilization events in a sequential manner. However, these assays depended on the translocation of periplasmic GFP to report on the permeability of the IM and OM of the peptide-treated Gram-negative bacteria. Because GFP is a globular protein (27 kDa), these assays could not explain how a small peptide could reach the IM and permeabilize it to GFP without permeabilizing the OM to GFP. The Weisshaar lab developed another single-cell, time-resolved assay, using the HaloTag concept and fluorescence microscopy, to assess OM permeabilization by peptides (Figure 12a).^370^ This assay utilizes the profluorophore JF_646_ ligand (702 Da) and a strain of *E. coli* that exports the HaloTag protein (34 kDa) to the periplasm. The JF_646_ ligand cannot cross the intact OM of Gram-negative bacteria. Following the addition of an MTP, the JF_646_ ligand enters the periplasm, binds to the HaloTag protein, and then fluoresces red, signaling the permeabilization of the OM to small molecules. Then, the entry of the HaloTag-JF_646_ conjugate into the cytoplasm indicates permeabilization of the IM to globular proteins, while dissipation of the conjugate indicates permeabilization of the OM to globular proteins. This assay can be modified with the addition of a DNA-binding dye to also detect permeabilization of the IM to a small molecule. Taken together, this assay can reveal, in sequence, the permeabilization of either the IM or the OM to either small molecules or globular proteins. However, it is important to consider that while this assay is a measure of translocation of small molecules across the bacterial membrane, it is not a direct measure of peptide translocation. This assay relies on the presumption that if the membrane is permeable to dyes, then it is also permeable to other small molecules such as peptides.

**Figure 12:**
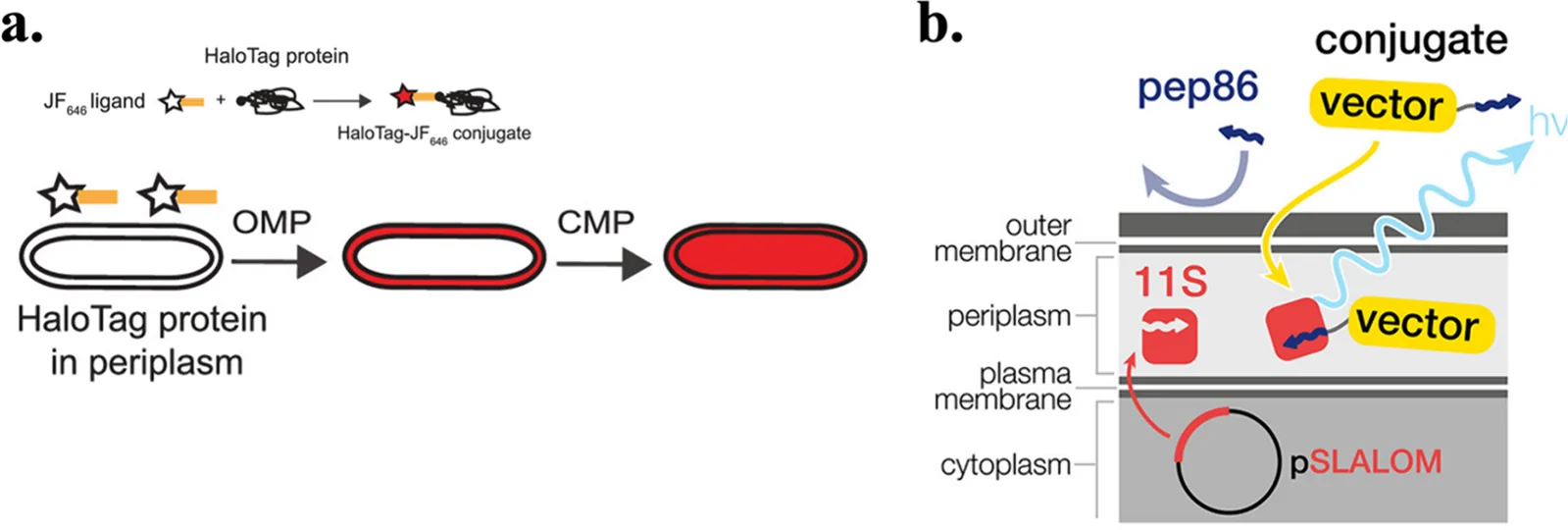
HaloTag and SLALOM for the measurement of peptide translocation into live bacterial cells. (a) In the HaloTag assay, *E. coli* expressing periplasmic HaloTag are treated with an MTP and JF_646_ ligand, which is impermeable to intact cells. Following MTP-mediated outer membrane permeabilization (OMP), JF_646_ ligand enters the periplasm, binds to HaloTag, and fluoresces red. Inner membrane permeabilization (CMP) results in the entry of the HaloTag-JF_646_ conjugate into the cytoplasm. (b) In SLALOM, Gram-negative bacteria expressing periplasmic 11S are treated with an MTP (vector) conjugated to pep86. Following entry of MTP-pep86 into the periplasm, pep86 binds to 11S to form a functional luciferase enzyme, allowing for the detection of peptide translocation across the outer membrane. Adapted with permission from ref 370. Copyright 2018 American Chemical Society. Adapted with permission from ref 371. Copyright 2020 American Chemical Society.

#### 4.3.6 SLALOM

The split luciferase assay for live monitoring of outer membrane transit (SLALOM) provides a quantitative, compartment-specific measurement of OM permeability (Figure 12b).^371^ This assay utilizes a split luciferase enzyme that becomes functional through the association of its two components: 11S and pep86 (a decapeptide). Gram-negative bacteria are genetically engineered to export 11S into the periplasm, and pep86 is applied to the bacteria exogenously. Because unmodified pep86 cannot cross the OM, the luciferase enzyme remains nonfunctional and there is no luminescent signal. However, once pep86 is conjugated to a vector (such as a peptide) capable of translocating across the OM, pep86 can enter the periplasm and associate with 11S to complete a functional luciferase enzyme. The luminescent signal is proportional to the amount of pep86 that has crossed the OM. SLALOM can be used to evaluate the ability of peptides to traverse the OM and carry cargo (pep86) into the periplasm. Thus, this assay provides a direct measure of peptide–cargo translocation across the OM of Gram-negative bacteria without the need for fluorescent labeling of the peptide. However, this assay does not measure translocation across the IM and does not provide information regarding the pathway of translocation across the OM.

#### 4.3.7 Functional Assays

The most direct way to demonstrate peptide translocation across a membrane, without labeling the peptide with a confounding fluorophore, is through functional assays that measure the intracellular effect of the cargo that the peptide delivers. One well-studied class of cargo is antibacterial antisense PNAs, which inhibit cell growth by binding to bacterial DNA or RNA. Antisense PNAs can be conjugated to an MTP to increase their ability to cross into bacterial cells. To directly evaluate the ability of peptides to translocate across bacterial membranes and deliver their PNA cargoes, researchers can measure the PNA-mediated inhibition of specific genes using biochemical assays that report on the activity of the gene product. For example, to measure the translocation of peptide-PNAs that target the β-galactosidase gene (*lacZ*) in *E. coli*, Good et al. assayed the inhibition of β-galactosidase activity using the chromogenic substrate o-nitrophenyl-β-galactoside as a reporter.^372–374^ Alternatively, the expression level of the genes targeted by the PNAs can be assayed using RT-qPCR to quantify mRNA levels, or Western blotting to measure protein expression.^375,376^ MTP-PNA chimeras have also been developed against the intracellular bacterial pathogen *Listeria.* ^337^

## 5 Properties of Membrane-Traversing Peptides

### 5.1 Physical Properties of MTPs: MTP Databases

Membrane-traversing peptides are a diverse group of peptides unified by the ability to cross membranes. They include linear, cyclic, bicyclic, branched, and dendrimeric architectures, and have a wide variety of secondary and tertiary structures: α-helical, β-structured, polyproline-like, or random coil.^1,20–27,113,211^ MTPs can function by a wide variety of mechanisms, regions of the mechanistic landscape of membrane translocation, ranging from transient interfacial association and monomeric translocation to membrane remodeling and massively cooperative membrane crossing. This structural plasticity distinguishes MTPs from most protein domains, suggesting that their function results from a balance of electrostatics, amphiphilicity, conformational dynamics, and interaction with the membrane.

#### 5.1.1 Sequence Space and General Properties

Large databases of MTPs have revealed characteristics that are shared across mechanistic classes. The CPPsite3 database,^1^ currently the most comprehensive resource for MTPs, contains more than 7000 peptides that have been reported to enter cells. Inclusion is based solely on evidence of cell entry rather than mechanistic detail. Some sequence redundancy is present—canonical examples such as the HIV TAT peptide, penetratin, and polyarginine sequences, especially Arg_9,_ are represented multiple times—but the breadth of the data set, containing >4100 unique sequences, provides meaningful statistical insights.^1^

The majority of MTPs in the database are linear (91%), Figure 13A, and composed exclusively of l-amino acids (77%), Figure 13A,B, although the proportion of cyclic, d-amino acid, and mixed-chirality peptides has increased steadily as design efforts have emphasized protease resistance and structural preorganization.^1^ Sequence lengths span from 5 to 37 residues (95% confidence interval), with a median of approximately 15 residues, Figure 13C. This size range is small enough to allow rapid conformational exchange and membrane insertion yet large enough to permit multivalent electrostatic interactions with membrane components such as anionic lipids, glycosaminoglycans, or lipid headgroups.^20–27^

**Figure 13:**
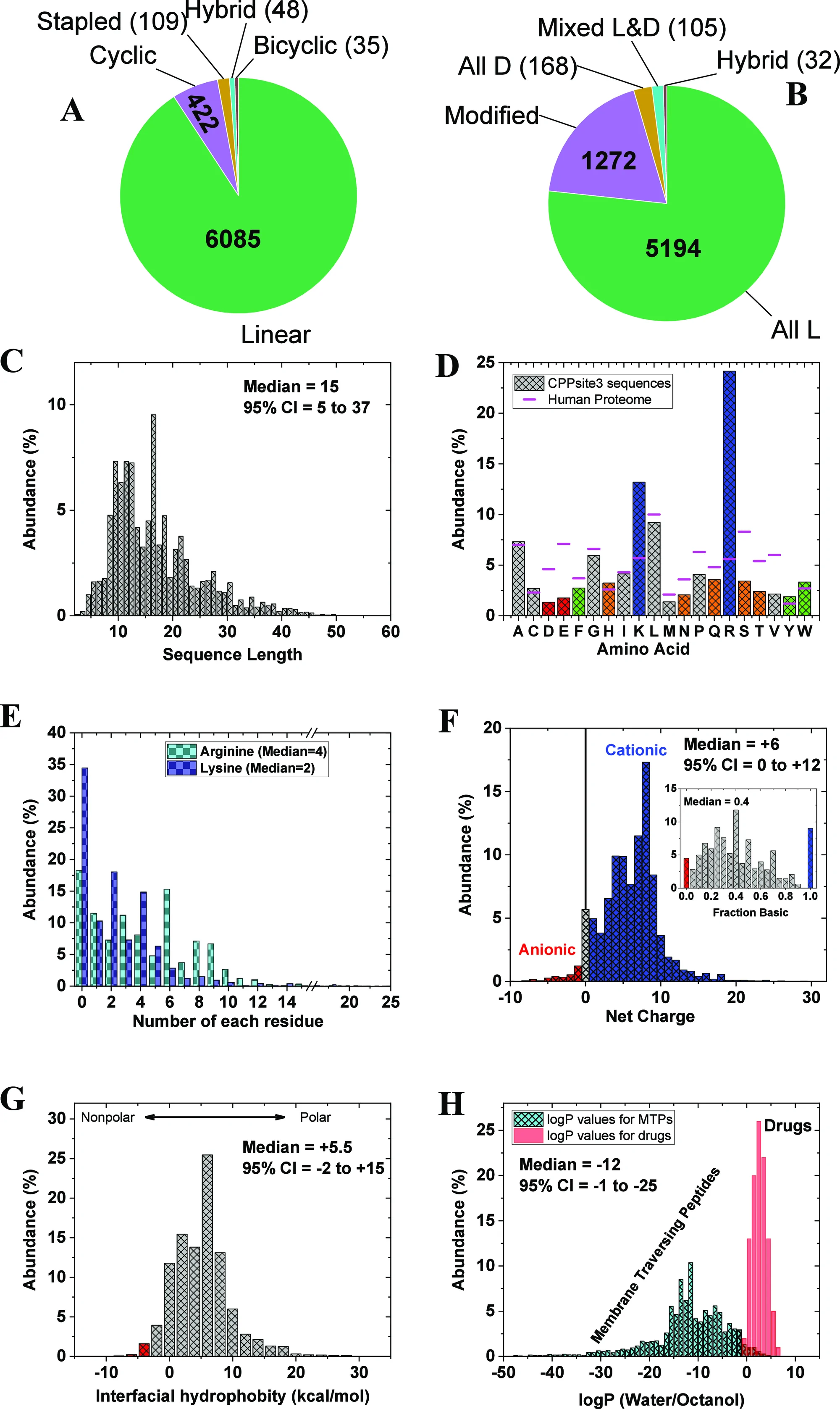
Characteristics and statistics of the CPPsite3 database of >8000 MTP sequences^1^ (A) Distribution of peptide architectures. (B) Distribution of peptide chirality. (C) Distribution of peptide length. (D) Abundance of the natural amino acids in the database (bars) compared to their abundance in the human proteome (horizontal lines). (E) Distribution of the number of arginine and lysine residues. (F) Distribution of the net charge. (G) Distribution of the interfacial hydrophobicity in the database. The red bars indicate peptides that are predicted to bind weakly, but measurably to phosphatidylcholine bilayers. (H) Distributions of log *P* values for peptides in the CPPsite3 database and small molecules drugs, for comparison.

#### 5.1.2 Amino Acid Composition and Charge Distribution

The amino acid composition of MTPs is vastly different from that of the human proteome overall, reflecting selective enrichment for membrane crossing, Figure 13D. Basic residues, especially arginine, are highly overrepresented. Arginine is four times more abundant in MTPs compared to the human proteome, while lysine is twice as abundant. In the CPPsite3 data set, 82% of peptides contain at least one arginine, and 65% contain at least one lysine, with some sequences carrying more than 15 basic residues. This is almost certainly because most biological membranes, including organelle membranes, are anionic. The conclusion that MTP activity is dominated by electrostatic interactions with anionic membranes is supported by the observation that acidic and other polar residues are severely underrepresented, with Asp and Glu having only one-fifth of the abundance in the CPPsite3 database that they have in the human proteome. Aromatic residues, which make significant contributions to hydrophobic partitioning into membrane interfaces,^377^ are not particularly abundant in MTP databases.

The majority of known MTPs are strongly cationic. The median net charge at physiological pH is approximately +6, with the most probable value around +9, Figure 13E. Nearly 10% of known sequences are 100% cationic; these are primarily polyarginine sequences, Figure 13F. The median fraction of positively charged residues across all sequences is 40%, an extraordinarily high value compared with the typical value of about 11% found in soluble peptides or proteins. Neutral and anionic MTPs are rare and often rely on specialized structural motifs or environmental triggers (e.g., pH-dependent protonation) to achieve membrane crossing.^154,159,378^

#### 5.1.3 Hydrophobicity and Membrane Partitioning

Unlike classical membrane-active peptides such as membrane-permeabilizing antimicrobial peptides or pore-forming toxins,^116,117,379,380^ MTPs are not predominantly hydrophobic. Hydrophobic partitioning into the membrane can be quantified by the Wimley–White interfacial hydrophobicity scale, which measures the free energy change associated with transferring a whole residue amino acid from water to a membrane interface.^68^ For unstructured peptides, a total interfacial free energy of approximately −4 kcal/mol corresponds to barely detectable binding. Values below –7 kcal/mol indicate strong and spontaneous partitioning into zwitterionic membranes. Remarkably, very few of the 7000+ sequences in the CPPsite3 database reach the −4 kcal/mol threshold for minimal measurable binding to uncharged bilayers, Figure 13G.

Electrostatic attraction, not hydrophobic partitioning, is the dominant driving force for MTP–membrane association. Many MTPs contain few or no aliphatic or aromatic residues yet bind to and cross cell membranes efficiently. Several well-characterized examples, such as the translocating peptides TP1, TP2, and TP3,^121,125^ can traverse membranes without strong binding to the bilayer interface, suggesting that transient interactions or membrane deformations facilitate translocation. The fact that even this mechanistic class contains mainly cationic peptides suggests that interactions with anionic groups, especially lipid phosphates, may be a critical part of the translocation mechanism. These behaviors are not explained by classical models of membrane translocation based on the partitioning of amphiphiles, and underscore that MTPs operate in a distinct physicochemical regime.

#### 5.1.4 Comparison to Small Drug Molecule Membrane Permeation

Membrane permeability of small molecules is relatively well described by their octanol–water partition coefficient (log P), which quantifies the balance between polar interactions and the hydrophobic effect^18,19,108,381^ and measures the propensity of a molecule to be soluble in the hydrocarbon core of the bilayer. Small-molecule drugs, which generally must passively diffuse across membranes while also being soluble in water, typically fall within a narrow log P range of ∼1–4, Figure 13H. In contrast, calculated log *P* values for MTPs span a wide and often extreme range of log *P*, most of which fall outside the window compatible with passive diffusion across membranes by virtue of hydrocarbon solubility. This disparity underscores that most MTPs do not cross membranes via equilibrium solubility–diffusion mechanisms.^18,19,381^ Instead, their permeation likely involves complex, multistep, and cooperative processes that we discuss in detail elsewhere in this review.

MTPs occupy a unique physicochemical niche: they combine high net charge, moderate or low hydrophobicity, and conformational flexibility yet display an intrinsic ability to penetrate lipid bilayers. These properties suggest that membrane translocation is governed not by classical thermodynamic partitioning but by dynamic coupling between peptide conformational ensembles and the physical state of the bilayer.^61,99,100^ Understanding these relationships quantitatively remains a major challenge, but the physical data from large peptide data sets such as CPPsite3 ^1^ now provide a foundation for predictive models^382–385^ that connect sequence, structure, and mechanism in the diverse landscape of MTPs.

### 5.2 Prediction of Membrane-Traversing Peptides by Machine Learning

Machine learning (ML) has become a powerful tool in the design and discovery of MTPs, enabling the prediction, optimization, and generation of novel sequences. The rapid increase in the number of available sequences and the rapid improvement of machine learning methodologies have led to an extreme proliferation of ML-based CPP/MTP predictors. Initially, researchers used classical classifiers like support vector machines and random forests that use sequence-derived physicochemical and compositional features.^1^ Now, deep-learning approaches, including those that are interpretable,^386^ are abundant. Some are designed to be used for the generation of novel CPPs.^387^ These modern ML approaches predict quantitative uptake values across cargo types and cell lines. These many ML strategies may accelerate MTP design, reduce the experimental workload, and provide mechanistic insights into the molecular features of cellular uptake. A partial list of CPP predictors described in the past few years includes MLLCPP 2.0,^382,388^ AiCPP,^385^ PractiCPP,^384^ SiameseCPP,^389^ PerseuCPP,^386^ LightCPPgen,^387^ PLM4CPPs,^390^ and CPPCGM.^391^ Although there are important caveats, recent ML predictors perform relatively well, using established databases, as judged by criteria such as the area under curves (AUC) for receiver operator characteristic curves or the Matthews correlation coefficient.^392^ However, caution is needed with the application of ML algorithms as the interaction of MTPs with membranes is studied using widely varying samples, conditions, and criteria for success.

#### 5.2.1 Experimental Validation

Pentelute and colleagues published a validated example of ML-driven CPP design using phosphorodiamidate morpholino oligonucleotides (PMOs), a class of antisense therapeutics, as cargo.^393^ The authors trained a random forest classifier using a library of 64 PMO–CPP conjugates. They then used the model to design seven new CPP sequences. When tested for PMO delivery in HeLa-654 cells, all seven predicted positives showed at least some increase in PMO delivery activity.

In a recent deep-learning-based study, a hybrid approach was applied using the predictor AiCPP^385^ to a region of the amyloid precursor protein (APP). A potential 17-aa wild-type MTP was identified. These authors then generated a library of sequence variants and experimentally validated a small number of active MTPs. Ten peptides (including the wild-type) were synthesized and tested in cells; nine of these variants were predicted to have higher penetration. These optimized peptides had improved membrane traversing ability compared to the original wild type, validating the utility of the model for optimization.

### 5.3 How Many Peptides Must be Delivered to the Cytosol to be Useful?

The cargo delivery literature can be difficult to interpret because “success” is defined in many ways. Some assays report a positive result after delivery of a single molecule of cargo. This is true when the cargo has an amplified effect, as for an enzyme, transcription factor, or splice correction molecule. On the other hand, other assays may require millions of cargo molecules delivered to each cell before returning a positive result. This would be the case for stoichiometric effects such as steric blockage of a protein–protein interaction in a signaling network.

To provide context for interpretation of the literature, in this section we examine the relation between the number of cargo molecules delivered to the cytosol and the absolute concentration of cargo in the cytosol. Assuming an aqueous volume of about 10^–12^ liters for a typical eukaryotic cell,^394^ the number of cargo molecules required to achieve specified cytosolic concentrations can be calculated, see Table 1. In comparison, copy numbers of signaling proteins in cells range from 10^2^ to 10^6^ per cell, and the median protein copy number in one cancer cell line was reported to be about 18,000/cell.^394,395^ This means that internal protein concentrations range from 0.0002 μM (200 pM) to 2.0 μM, with a median of ∼0.04 μM (Table 1). Delivery of 10,000 cargo molecules is thus likely to have significant biological activity if the cargo acts stoichiometrically on a signaling molecule in a cell. For comparison, in a typical cell culture experiment there could be 10^15^ copies of a cargo molecule added to the cell culture medium of each well (10 μM cargo × 200 μL volume). Thus, a fractional delivery efficiency well below 1 × 10^–12^ could lead to “success”. We urge the community to use quantitative measures of delivery, such as absolute cytosolic concentration or fractional endosomal escape, as we discussed earlier.

**Table 1:** Estimate of the Cytosolic Concentrations That Are Achieved by Delivery of Protein Cargos to Cells; We Assume a Typical Aqueous Volume of a Eukaryotic Cell to Be Roughly 10^–12^ Liter

delivery arithmetic	
copy number	concentration
10,000	0.02 μM
100,000	0.2 μM
1,000,000	2.0 μM

## 6 Illustrative Examples of Membrane-Traversing Peptides

MTPs have attracted attention for many potential applications because of their demonstrated ability to deliver macromolecular cargo to cells. Despite this, no MTP or MTP-containing complex has made its way past Phase III human clinical trials. Some major challenges, such as low endosomal escape efficiency, targeting specificity, low bioavailability, and toxicity, will need to be addressed for MTPs to be approved for wider use. Here, we highlight a few of the many examples of MTPs or MTP families with properties that drive optimism for the eventual translation of these peptides into the biotechnology workspace or clinic.^396–405^

### 6.1 Spontaneous Membrane-Traversing Peptides

Spontaneous membrane translocating peptides (SMTPs) have been defined *by us* as peptides that can cross a membrane “silently,” without endocytosis, without significant cooperativity or surface aggregation, and without membrane permeabilization.^10,121,125^ SMTPs can be linear, cyclic, stapled, etc., but are unified by the ability to cross membranes at low concentrations. We previously screened a library of 10,368 peptides containing aliphatic, aromatic, and cationic amino acids to find members that could translocate into synthetic liposomes made from 90% zwitterionic phosphatidylcholine (PC) and 10% anionic phosphatidylglycerol (PG) while simultaneously not causing measurable membrane permeabilization.^10^ This screen led to the identification of spontaneous membrane translocating peptides that we call TP1, TP2, and TP3.^4,10,121,125^ Interestingly, these peptides cross bilayers made from 100% PC, showing that anionic lipids are not needed for translocation,^10,125,126^ even though each peptide contains multiple conserved arginine residues. These arginines are found in a motif with a consensus of LRLLR which we showed later to be both necessary and sufficient for translocation.^126^ SMTPs enter the cytosol of live cells by directly crossing the plasma membrane^10,125,4,212^ at very low concentrations. They do not require endocytosis, and they do not act cooperatively. They have been shown to deliver polar fluorescent dyes as well as the bioactive cyclic peptide, phalloidin, to live cells.^127^

Several groups have used simulations to study the translocation properties of the TP2 families. Lu and Patel used Martini coarse-grained simulations of PC/PG for TP1, TP2, and TP3. They found significantly lower translocation energy barriers (lower than 20 kcal/mol) compared with the fully cationic Arg_9_ peptide, which does not translocate across the same bilayers, suggesting a mechanistically different translocation process. However, the calculated free energy barriers were still much too large to match experimental observations for the SMTPs, pointing to potential limitations in the simulation of membrane translocation.^132^ Cao et al. simulated peptides, in membranes, with combinations of three leucine and two arginine residues, focusing on five sequences composed of leucine and arginine (LLRLR, LRLLR, LLLRR, RLLLR, LRLRL).^406^ They showed that the balance and spatial arrangement of hydrophobic (Leu) and hydrophilic (Arg) residues quantitatively modulate membrane insertion depth, translocation free-energy barriers, and the stability of interfacial versus fully translocated states. In qualitative agreement with experiments, these authors reported that certain sequences, particularly those with clustered hydrophobic residues, achieve more efficient penetration with lower free-energy barriers and higher probabilities of adopting membrane-spanning conformations during multimicrosecond equivalent sampling, although the barriers measured are still higher than experiment.^406^

### 6.2 Stapled and Cyclic MTPs

Stapled peptides are defined by the presence of a nonlabile covalent cross-link that stabilizes secondary structure and thus improves both membrane translocation and target recognition.^407,408^ The best studied stapled peptides are short α-helical sequences chemically constrained by an all-hydrocarbon “staple” formed between non-natural, olefin-bearing amino acids (usually placed at i/i+4 or i/i+7) via ruthenium-catalyzed olefin metathesis.^235,408,409^ This approach is potentially useful because many intracellular signaling interfaces can potentially be mediated by short helices. Furthermore, hydrocarbon stapling can improve membrane translocation. An interesting example of this comes from the stapled BH3 helix studies of Korstmeyer and colleagues.^235,236^ By stapling BH3 sequences, the authors converted disordered BID/BIM peptides into stable, protease-resistant helices that bind canonical BCL-2 family proteins. These peptides bind with higher affinity, enter cells, and trigger mitochondrial apoptosis. Using biophysical approaches, cell biology, and murine xenograft models for *in vivo* efficacy, they showed that stapled helical peptides can enter cells, bind to BCL-XL and BAX proteins, and suppress tumor growth *in vivo*.

There are also many examples of cyclic MTPs described in the literature. For example, cyclic variants of the spider peptide gomesin have been designed and studied by Henriques and colleagues.^124^ These redesigned host–defense peptides do not disrupt eukaryotic cell membranes while efficiently translocating, in a preferential way, into several types of cancer cells. The gomesin analogs translocate into cancer cells at very low concentration (250 nM) suggesting that cooperativity is probably not needed for translocation. This suggests that they can enter cells by direct plasma membrane translocation.^124,410^ Another good example of cyclic MTPs is found in the bicyclic peptides that are stapled by bismuth. These have been developed and studied by Nitsche^411–414^ and others. Bismuth bicycles have been reported to undergo energy-dependent cell uptake via endocytosis.^414^ Pei and colleagues^21,61^ have also designed and studied cyclic MTPs, finding that the cyclic polyarginine peptides enter cells directly through the plasma membrane using a cooperative mechanism of “vesicle budding and collapse”, a mechanism that we discuss in more detail below.

### 6.3 Classical Cell Penetrating Peptides

Classical CPPs are usually short, cationic, arginine-rich sequences. The best studied MTP is the HIV-1 TAT peptide and peptides derived from TAT, such as Arg_9_. Classical CPPs have been developed and widely used to enhance intracellular delivery of macromolecules such as antisense oligonucleotides.^415–417^ One of the prominent examples of this application was the conjugation of CPPs to phosphorodiamidate morpholino oligomers (PMOs) for splice-switching therapy, particularly in the context of Duchenne muscular dystrophy (DMD). Dystrophin is a large cytoskeletal protein that links the actin cytoskeleton of muscle fibers to the extracellular matrix, and its absence or severe reduction due to mutations in the DMD gene leads to progressive muscle degeneration characteristic of Duchenne muscular dystrophy. The challenge has been to efficiently deliver the PMOs to skeletal and cardiac muscle. Kole and colleagues^418,419^ demonstrated that peptide-conjugated PMOs (PPMOs) could induce sustained dystrophin expression in mdx mice by dramatically improving antisense uptake and exon-skipping efficiency. They covalently linked the PMOs to arginine-rich CPPs. In this case, the peptide was composed of repeating arginine (R) and 6-aminohexanoic acid (X) residues referred to by the authors as “RXR”. The authors conducted a systematic *in vivo* functional screen of related CPP variants using an EGFP splice-correction in mice by intravenous injection at 3–12 mg/kg/day for 4 days—and evaluated using Cy5-labeled oligonucleotides, by *in vivo* fluorescence imaging, nested RT-PCR for dystrophin exon 23 skipping, and immunoblotting and immunofluorescence for dystrophin protein detection. The results were durable dystrophin restoration across multiple skeletal muscle groups, detectable cardiac expression, reduced serum creatine kinase levels, and improved muscle histology compared with unconjugated PMOs. However, cardiac delivery remained comparatively limited, and higher doses revealed some dose-dependent toxicity, reinforcing the importance of optimization. This study showed that classical CPPs could bypass many previous challenges in antisense therapy.

Another example of CPP applications comes from the ATTEMPTS (antibody-targeted-triggered-electrically modified-prodrug-type-strategy) system designed by the Yang Lab.^416,417,420–422^ In this system, they take advantage of the classical CPP mechanism to create a targeted and switchable prodrug for tumors. Instead of relying on natural uptake, they use a heparin-conjugated antibody to target the tumors and attach a protein drug to the CPP. This assembles into a stable complex through electrostatic interactions while also “masking” the CPP, limiting uptake and degradation. To unmask the CPP, once the complex accumulates on tumor cells, protamine sulfate is used to replace the CPP in the complex and allow it to translocate into the target cells. This strategy was applied to a colorectal cancer model where TAT-gelonin was attached to heparin-conjugated anti-CEA mAB (T84.66-Hep).^422^ This complex showed an enhanced tumor accumulation and reduced off-target toxicity. The strategy was also evaluated in an acute lymphoblastic leukemia model that used TAT-asparaginase and supported the use of ATTEMPTS for a diffuse disease treatment.^416,417,420^

Azurin-p28 represents a distinct class of MTPs, where it is both the vehicle and the cargo.^423–429^ It was derived from a bacterial redox protein, azurin, by Das Gupta and colleagues. p28 is an amphipathic peptide that preferentially enters malignant cells over normal cells by caveolin-dependent endocytosis. They showed that p28 can inhibit proteasomal degradation of p53, stabilizing both wild-type and some mutant forms, and consequently induce cell cycle arrest. Azurin-p28 is currently one of the few MTPs that has made it to clinical trials. In a phase I trial with advanced p53-positive solid tumors, p28 was shown to be well-tolerated and had no negative responses from the patients.

### 6.4 The PepFect Family

The PepFect family represents an important story in MTP engineering. These MTPs were developed by Langel and colleagues over many years to address the low oligonucleotide transfection efficiencies and endosomal entrapment of early generation vectors. Derived from the transportan 10 peptide template,^173,430,431^ PepFect peptides feature strategic amino acid substitutions, such as replacing lysines with ornithines, to reduce protease sensitivity, improving stability and performance.^432^ The defining breakthrough in the development of these peptides was the covalent attachment of a hydrophobic fatty acid, typically stearic acid, to the N terminus. This modification yields a highly amphipathic and hydrophobic structure, allowing PepFect molecules to spontaneously self-assemble with negatively charged oligonucleotide cargos through noncovalent interactions, forming relatively stable nanoparticles that (i) enable uptake into cells, (ii) protect the genetic cargo from enzymatic degradation, and (iii) enable endosomal escape of the cargo.^432–436^

Mechanistically, PepFect nanocomplexes exploit specific cellular entry pathways and subsequently increase endosomal escape by a mechanism that is still not fully understood. The positively charged peptide assemblies interact electrostatically with negatively charged cell surface proteoglycans, promoting uptake predominantly through macropinocytosis and scavenger-receptor-mediated endocytosis. Once trapped within the intracellular vesicle, the unique chemical architecture of PepFect likely facilitates endosomal escape. As the endosome acidifies, the amphipathic peptide likely undergoes a structural transition that temporarily destabilizes the endosomal membrane, allowing the cargo to slip into the cytosol before the vesicle fuses with degrading lysosomes. However, the mechanistic details of endosomal escape have not been well understood. This capacity for efficient, nontoxic cytosolic release has been systematically documented across several distinct chemical variations in the family,^432–442^ including pH-responsive variants.^439^

PepFects represent potential alternative delivery strategies to lipid nanoparticles for certain nucleic acid applications, and they are examples in RNA interference and splice-correction technologies. For example, PepFects have demonstrated high efficacy in transporting small interfering RNA and antisense oligonucleotides, which have been used to correct Duchenne muscular dystrophy, a genetic disorder, in preclinical cell culture models.^432^ Research has expanded the utility of PepFects to include gene-silencing applications in highly sensitive human embryonic stem cells^435,436^ as well as managing complex inflammation responses by transporting microRNA mimics.^438^ Furthermore, because PepFect nanocomplexes possess the ability to be lyophilized into stable, solid-state formulations without losing their transfection capability, they offer a more robust alternative to traditional lipid nanoparticles.^432,441^

### 6.5 Peptides that Form Membrane-Coupled Puncta

Some MTPs, particularly highly cationic and/or conformationally constrained arginine-rich peptides, enter cells by a rapid, non-endocytic mechanism.^5,21,61,443–446^ A defining feature of this pathway is the spontaneous formation of transient, membrane-coupled puncta that are visible by confocal microscopy, sometimes within seconds of peptide addition. Because these structures form and delivery occurs, even at 4 °C when active cellular processes are inhibited, their formation must arise primarily from cooperative peptide–lipid interactions rather than active cellular participation.

We propose the term *membrane coupled puncta* (MCP) to describe this broader mechanistic class. This terminology is intended to encompass a range of observable membrane-associated peptide–lipid assemblies without presupposing a specific architecture. Experimentally, visible puncta can be large, μm-scale, three-dimensional structures. Smaller structures are likely to be common but are below the resolution limit of fluorescence microscopy. Within the mechanistic landscape shown in Figure 3, such puncta may belong to either of the lower quadrants: three-dimensional structures dominated by lamellar bilayer organization, such as vesicles or stacked multilamellar assemblies, or structures enriched in nonbilayer lipid phases. Both scenarios are likely to occur in different systems, although distinguishing between them experimentally remains challenging and has been addressed carefully by only a few groups.

Pei and co-workers have characterized this type of uptake for cyclic and polycationic CPPs and have described the process as “vesicle budding and collapse”.^21,61–63^ Their interpretation is supported by evidence that the puncta they observe contain peptides, lipids, and water-soluble cargo, and that some puncta are particles that appear capable of separating from the bulk membrane. Importantly, related behaviors have also been observed at endosomal membranes following peptide uptake, suggesting that similar membrane remodeling processes may occur at multiple stages of intracellular trafficking and may occur for both energy dependent and energy independent cell entry.

We have described an illustrative example of MCP in the hybrid CPP P17, Figure 14, which was identified in a screen for peptide nucleic acid delivery.^5,6^ When TAMRA-labeled P17 is added to HeLa cells at 5 μM, membrane binding begins within seconds. Bright membrane puncta appear within approximately 10 s and rapidly increase in intensity, through recruitment of additional peptide and anionic lipids. By roughly 60 s, puncta intensity begins to decline while diffuse cytosolic fluorescence rises sharply. Within approximately 2 min, cytosolic peptide levels approach steady state and most puncta diminish substantially. These observations imply a membrane crossing process mediated by structures with dimensions approaching or exceeding 1 μm, sufficiently large to contain thousands to millions of peptide molecules together with substantial amounts of lipid and aqueous cargo. For example, a 300 nm particle composed of approximately 50% peptide could contain on the order of five million peptide molecules.

**Figure 14:**
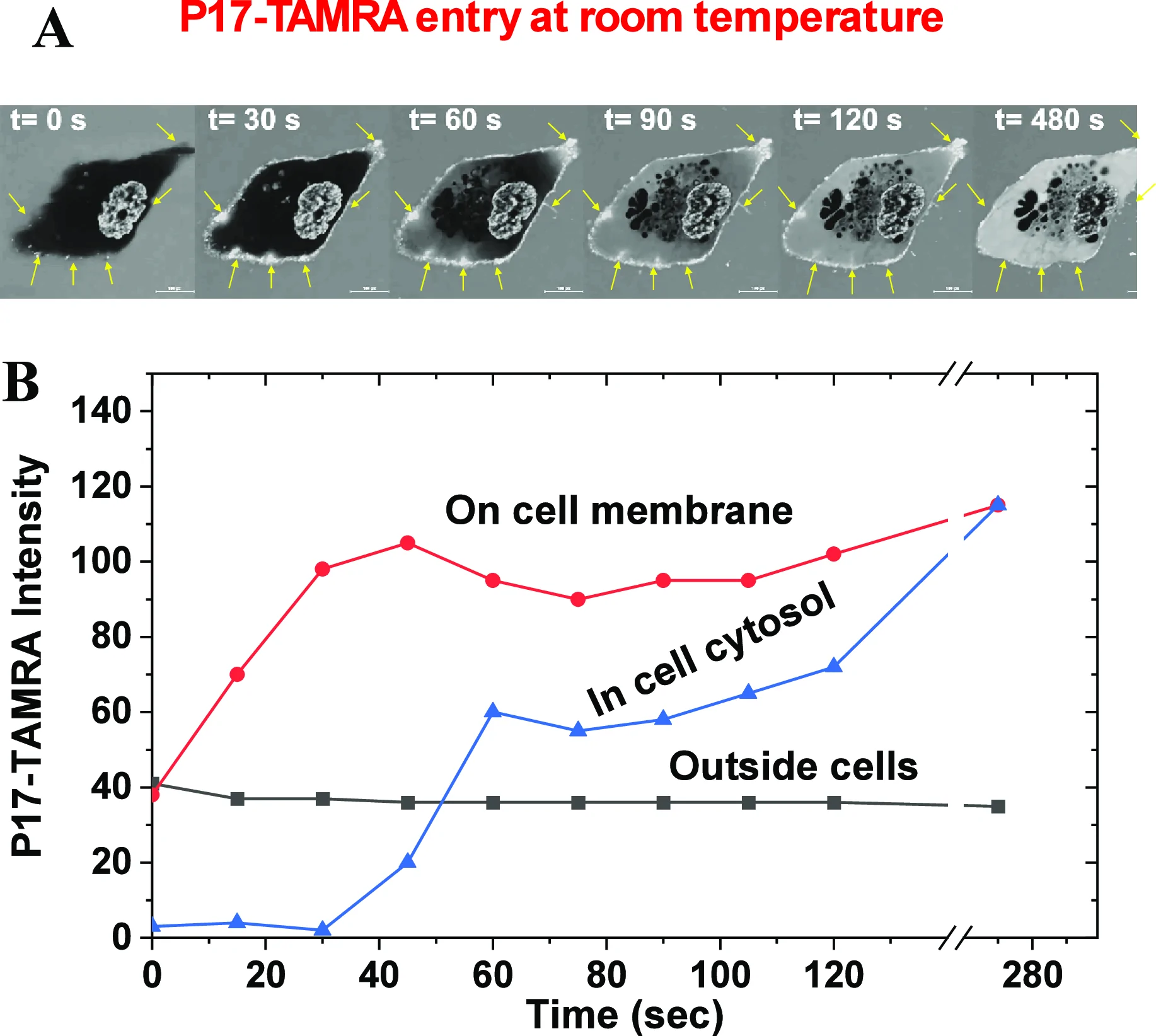
Very rapid entry of an MTP into the cytosol by direct crossing of the plasma membrane by membrane associated puncta-mediated delivery. (A) Addition of dye labeled P17 ^5,6^ is followed within 10 s by plasma membrane binding and formation of large, bright membrane-coupled puncta (arrows). In less than 1 min, dye-labeled peptide has started to enter the cytosol. (B) Time courses of peptide concentration measured in solution, in the cytosol, and on the membrane (excluding obvious puncta). Under these conditions, this membrane traversing peptide crosses into the cell cytosol on a time scale of tens of seconds, occurring after a lag phase of about 40 s.

A notable feature of MCP-mediated delivery is that uptake efficiency is often relatively insensitive to the identity of the attached cargo.^5^ This observation suggests that cargo may remain within an aqueous environment throughout the translocation pathway. At the same time, many mechanistic questions remain unresolved. The peptide and lipid composition of the puncta, their internal lipid organization, the extent of aqueous compartmentalization, and the mechanism by which peptide and cargo are rapidly released into the cytosol are still poorly understood. Nevertheless, studies of MCP-dependent delivery have reframed direct membrane translocation as a cooperative, membrane-driven phenomenon involving large-scale peptide–lipid self-assembly, providing a mechanistic foundation for next-generation intracellular delivery systems.

### 6.6 Peptides with Covalent Surface Tethering

A study by Schneider et al.^131^ provides important insights into the direct plasma membrane crossing of MTPs and cargoes via cell surface aggregation, membrane deformation, and puncta formation. These authors used cyclic Arg_10_ sequences that can directly cross the plasma membrane, at least above a threshold concentration, and can promote the cytosolic delivery of attached protein cargoes. They found that chemical moieties that promote covalent cell surface attachment (e.g., free sulfhydryl groups or maleimides) greatly enhance the cell surface clustering and delivery of protein cargo by MTPs, including cargoes that are not conjugated to modified peptides. Near the peptide clusters on the cell surface, membrane deformation and changes in membrane tension were observed.^131^ They also showed that the delivery of peptide-conjugated cargo is enhanced by the addition of free peptides. Thus, the direct effects of free peptides on the membrane enable delivery of cargo, consistent with puncta-dependent delivery, or a vesicle budding and collapse-like mechanism.^61–63^ Overall, this work supports a hybrid mechanism in which covalent surface reactivity enhances surface clustering and recruitment of peptide, which, in turn, enhances localized changes to the membrane architecture to enable more efficient clustering of MTPs into puncta and subsequent delivery of large biomolecules. This framework expands the current understanding of MTP-mediated uptake by highlighting that membrane curvature and vesicle dynamics are important considerations for next-generation intracellular delivery strategies.

### 6.7 Liquid–Liquid Condensates

Futaki and colleagues^447,448^ demonstrated a novel process for intracellular delivery of immunoglobulin G (IgG) using a trimeric variant of the cationic amphiphilic lytic peptide L17E conjugated to an Fc binding motif. Mixing FcB-(L17E)_3_ with fluorescently labeled IgG induced the formation of liquid-droplet-like coacervates, which spontaneously translocated IgG into the cytosol in serum-containing medium. Efficient translocation required negative charges on IgG, and pharmacological studies demonstrated the involvement of actin-dependent membrane dynamics rather than simple pore formation or endosomal uptake and escape. Importantly, the approach was generalizable to other negatively charged proteins, including antinuclear pore antibodies and super negatively charged nanobody-GFP fusions, which retained their ability to engage intracellular targets after delivery. These findings suggest that liquid-droplet-mediated delivery overcomes traditional barriers associated with the cytosolic entry of large proteins, offering a simple, scalable, and potentially modular platform for the intracellular transport of therapeutic antibodies, engineered protein constructs, and other bioactive macromolecules. Beyond enabling functional studies of intracellular targets, this method may advance the development of protein-based therapeutics, facilitate high-content cellular imaging, and provide a versatile tool for chemical biology by harnessing the unique physicochemical properties of peptide-induced coacervates.^447–449^

## 7 Conclusions

Membrane-traversing peptides have emerged as tools for the intracellular delivery of a wide range of cargoes from small molecules and nucleic acids to proteins, nanoparticles, and genome-editing complexes. The diversity of peptide classes, uptake mechanisms, design strategies, and methods of study reviewed here highlights the complexity of their interactions with cells and membranes. At the same time, significant unanswered questions remain, including the precise molecular determinants that govern direct translocation versus endocytic uptake, the extent to which mechanisms vary across cell types and physiological contexts, and how cargo affects intracellular trafficking and fate. Additional challenges persist in achieving predictable targeting, endosomal escape, minimizing off-target effects and toxicity, and translating *in vitro* efficacy into *in vivo* and medically relevant applications. Addressing these gaps will require continued advances in peptide engineering, quantitative biophysics, assays, and imaging. As these efforts converge, MTPs are poised to play an increasingly transformative role in basic research, diagnostics, and therapeutics, provided that a deeper mechanistic understanding guides their rational design and application.

## References

[1] Bajiya N., Najrin S., Kumar P., Choudhury S., Tomer R., Raghava G. P. S. (2025). CPPsite3: An updated large repository
of experimentally validated cell-penetrating peptides. Drug Discov Today..

[2] Batistatou N., Kritzer J. A. (2024). Recent advances
in methods for quantifying the cell
penetration of macromolecules. Curr. Opin Chem.
Biol..

[3] He J., Hristova K., Wimley W. C. (2012). A highly
charged voltage sensor helix
translocates spontaneously across membranes. Angew. Chem. Int. Ed..

[4] Barba-Bon A., Pan Y. C., Biedermann F., Guo D. S., Nau W. M., Hennig A. (2019). Fluorescence Monitoring of Peptide Transport Pathways
into Large and Giant Vesicles by Supramolecular Host-Dye Reporter
Pairs. J. Am. Chem. Soc..

[5] Kauffman W. B., Guha S., Wimley W. C. (2018). Synthetic
molecular evolution of
hybrid cell penetrating peptides. Nat. Commun..

[6] Ferrie, R. P. Rapid Delivery of Bioactive Membrane-Impermeant Peptides into Cancer Cells by Synthetically Evolved Membrane-Actuve Peptides; Tulane University School of Medicine: New Orleans, LA; 2023.

[7] Wiener M. C., White S. H. (1992). Structure of a fluid dioleoylphosphatidylcholine bilayer
determined by joint refinement of x-ray and neutron diffraction data.
III. Complete structure. Biophys. J..

[8] Schow E. V., Freites J. A., Myint P. C., Bernsel A., von Heijne G., White S. H., Tobias D. J. (2011). Arginine
in Membranes: The Connection
Between Molecular Dynamics Simulations and Translocon-Mediated Insertion
Experiments. J. Membr. Biol..

[9] Hessa T., White S. H., von Heijne G. (2005). Membrane insertion
of a potassium-channel
voltage sensor. Science..

[10] Marks J. R., Placone J., Hristova K., Wimley W. C. (2011). Spontaneous membrane-translocating
peptides by orthogonal high-throughput screening. J. Am. Chem. Soc..

[11] Sun L., Hristova K., Bondar A.-N., Wimley W. C. (2024). Structural Determinants
of Peptide Nanopore Formation. ACS Nano.

[12] Hristova K., Wimley W. C. (2011). A Look at Arginine
in Membranes. J. Membr. Biol..

[13] Wheaten S., Ablan F. D. O., Spaller B. L., Trieu J. M., Almeida P. F. (2013). Translocation
of cationic amphipathic peptides across the membranes of pure phospholipid
giant vesicles. J. Am. Chem. Soc..

[14] Bayliss, W. M. Principles of General Physiology. Longmans, London, 1915.

[15] Meyer H. (1901). Zur theorie
der alkolnarkose: Der einfluss wechselnder temperatur sur wirkungsstarke
und theilungscoefficient dar narcotica. Naunyn
Schiedebergs Arch Pharmacol..

[16] Overton, E. Studien uber die Narkose; Gustav Fischer: Jena, 1901.

[17] White, S. H. , Engelman, D. M. , Von Heijne, G. Cell Boundaries: How Membranes and Their Proteins Work; Garland Science: Boca Raton, FL, 2021.

[18] Lipinski C.
A., Lombardo F., Dominy B. W., Feeney P. J. (2001). Experimental and
computational approaches to estimate solubility and permeability in
drug discovery and development settings. Adv.
Drug Deliv Rev..

[19] Veber D. F., Johnson S. R., Cheng H. Y., Smith B. R., Ward K. W., Kopple K. D. (2002). Molecular properties
that influence the oral bioavailability
of drug candidates. J. Med. Chem..

[20] Langel, U. CPP, Cell Penetrating Peptides. Langel, U , Ed.; Springer Nature: Singapore, 2023.

[21] Dougherty P. G., Sahni A., Pei D. (2019). Understanding Cell Penetration of
Cyclic Peptides. Chem. Rev..

[22] Kauffman W. B., Fuselier T., He J., Wimley W. C. (2015). Mechanism matters:
A taxonomy of cell penetrating peptides. Trends
Biochem. Sci..

[23] Zorko M., Jones S., Langel U. (2022). Cell-penetrating
peptides in protein
mimicry and cancer therapeutics. Adv. Drug Deliv
Rev..

[24] Juretic D. (2022). Designed Multifunctional
Peptides for Intracellular Targets. Antibiotics
(Basel).

[25] Futaki S., Arafiles J. V. V., Hirose H. (2020). Peptide-assisted Intracellular Delivery
of Biomacromolecules. Chem. Lett..

[26] Bechara Cr, Sagan S. (2013). Cell-penetrating peptides:
20 years later, where do we stand?. FEBS Lett..

[27] Sanchez-Navarro M. (2021). Advances in
peptide-mediated cytosolic delivery of proteins. Adv. Drug Deliv Rev..

[28] Herce H. D., Garcia A. E. (2007). Cell penetrating peptides: How do
they do it?. Journal of Biological Physics..

[29] Hong C. W., Zeng Q. (2014). Tapping the treasure
of intracellular oncotargets with immunotherapy. FEBS Lett..

[30] Cheever M. A., Allison J. P., Ferris A. S., Finn O. J., Hastings B. M., Hecht T. T., Mellman I., Prindiville S. A., Viner J. L., Weiner L. M., Matrisian L. M. (2009). The prioritization
of cancer antigens: a national cancer institute pilot project for
the acceleration of translational research. Clin. Cancer Res..

[31] Disis M. L., Cheever M. A. (1996). Oncogenic proteins
as tumor antigens. Curr. Opin Immunol..

[32] Gonzalez M. W., Kann M. G. (2012). Chapter 4: Protein interactions and disease. PLoS Comput. Biol..

[33] Ideker T., Sharan R. (2008). Protein networks in
disease. Genome Res..

[34] Hendzel M. J., Kruhlak M. J., MacLean N. A., Boisvert F., Lever M. A., Bazett-Jones D. P. (2001). Compartmentalization
of regulatory proteins in the
cell nucleus. J. Steroid Biochem Mol. Biol..

[35] Lazo J. S., Sharlow E. R. (2016). Drugging Undruggable Molecular Cancer
Targets. Annu. Rev. Pharmacol. Toxicol..

[36] Freidinger R. M. (2003). Design
and synthesis of novel bioactive peptides and peptidomimetics. J. Med. Chem..

[37] Goldsmith K. C., Liu X., Dam V., Morgan B. T., Shabbout M., Cnaan A., Letai A., Korsmeyer S. J., Hogarty M. D. (2006). BH3 peptidomimetics
potently activate apoptosis and demonstrate single agent efficacy
in neuroblastoma. Oncogene..

[38] Cunningham A. D., Qvit N., Mochly-Rosen D. (2017). Peptides and
peptidomimetics as regulators
of protein-protein interactions. Curr. Opin
Struct Biol..

[39] Sang J., Kulkarni K., Watson G. M., Ma X., Craik D. J., Henriques S. T., Poth A. G., Benfield A. H., Wilce J. A. (2019). Evaluation
of Cyclic Peptide Inhibitors of the Grb7 Breast Cancer Target: Small
Change in Cargo Results in Large Change in Cellular Activity. Molecules.

[40] Philippe G., Huang Y. H., Cheneval O., Lawrence N., Zhang Z., Fairlie D. P., Craik D. J., de Araujo A. D., Henriques S. T. (2016). Development of cell-penetrating peptide-based drug
leads to inhibit MDMX:p53 and MDM2:p53 interactions. Biopolymers..

[41] Segre D., Ben Eli D., Deamer D. W., Lancet D. (2001). The lipid world. Orig Life Evol
Biosph..

[42] Deamer D. (2016). Membranes
and the Origin of Life: A Century of Conjecture. J. Mol. Evol..

[43] Singer S. J., Nicolson G. L. (1972). The fluid mosaic
model of the structure of cell membranes. Science..

[44] White, S. H. , Wiener, M. C. The liquid-crystallographic structure of fluid lipid bilayer membranes. In Merz, K. M. , Roux, B , Eds.; Membrane Structure and Dynamics; Birkhäuser: Boston; 1996. pp 127–144.

[45] Sansom M. S., Smith G. R., Adcock C., Biggin P. C. (1997). The dielectric properties
of water within model transbilayer pores. Biophys.
J..

[46] Shelby S. A., Castello-Serrano I., Wisser K. C., Levental I., Veatch S. L. (2023). Membrane
phase separation drives responsive assembly of receptor signaling
domains. Nat. Chem. Biol..

[47] Levental I., Wang H. Y. (2020). Membrane domains
beyond the reach of microscopy. J. Lipid Res..

[48] van
Meer G., Voelker D. R., Feigenson G. W. (2008). Membrane lipids: where they are and
how they behave. Nat. Rev. Mol. Cell Biol..

[49] Koynova R., Caffrey M. (1998). Phases and phase transitions
of the phosphatidylcholines. Biochim. Biophys.
Acta.

[50] Koller D., Lohner K. (2014). The role of spontaneous lipid curvature
in the interaction
of interfacially active peptides with membranes. Biochim. Biophys. Acta.

[51] Levental K. R., Lorent J. H., Lin X., Skinkle A. D., Surma M. A., Stockenbojer E. A., Gorfe A. A., Levental I. (2016). Polyunsaturated Lipids
Regulate Membrane Domain Stability by Tuning Membrane Order. Biophys. J..

[52] Maniti O., Piao H. R., Ayala-Sanmartin J. (2014). Basic cell
penetrating peptides induce
plasma membrane positive curvature, lipid domain separation and protein
redistribution. Int. J. Biochem. Cell Biol..

[53] Prenner E. J., Lewis R. N. A. H., Neuman K. C., Gruner S. M., Kondejewski L. H., Hodges R. S., McElhaney R. N. (1997). Nonlamellar phases induced by the
interaction of gramicidin S with lipid bilayers. A possible relationship
to membrane-disrupting activity. Biochemistry..

[54] Bangham A. D. (1978). Properties
and uses of lipid vesicles: An overview. Ann.
N.Y. Acad. Sci..

[55] Allolio C., Magarkar A., Jurkiewicz P., Baxova K., Javanainen M., Mason P. E., Sachl R., Cebecauer M., Hof M., Horinek D., Heinz V., Rachel R., Ziegler C. M., Schrofel A., Jungwirth P. (2018). Arginine-rich
cell-penetrating peptides
induce membrane multilamellarity and subsequently enter via formation
of a fusion pore. Proc. Natl. Acad. Sci. U.
S. A..

[56] Marsh, D. CRC Handbook of Lipid Bilayers; CRC Press: Boca Raton, 1990.

[57] Cullis P. R., de Kruijff B. (1978). Polymorphic phase behavior of lipid mixtures as detected
by ^31^P NMR. Evidence that cholesterol may destabilize bilayer
structure in membrane systems containing phosphatidylethanolamine. Biochim. Biophys. Acta.

[58] Jackson M. B., Sturtevant J. M. (1978). Phase behavior of lipids from *Halobacterium
halobium*. Biochemistry..

[59] Goldfine H., Johnston N. C., Phillips M. C. (1981). Phase behavior of ether lipids from *Clostridium butyricum*. Biochemistry..

[60] Brown P. M., Steers J., Hui S. W., Yeagle P. L., Silvius J. R. (1986). Role of
head group structure in the phase behavior of amino phospholipids.
2. Lamellar and nonlamellar phases of unsaturated phosphatidylethanolamine
analogues. Biochemistry..

[61] Sahni A., Ritchey J. L., Qian Z., Pei D. (2024). Cell-Penetrating Peptides
Translocate across the Plasma Membrane by Inducing Vesicle Budding
and Collapse. J. Am. Chem. Soc..

[62] Sahni A., Pei D. (2021). Bacterial Toxins Escape the Endosome by Inducing Vesicle Budding
and Collapse. ACS Chem. Biol..

[63] Sahni A., Qian Z., Pei D. (2020). Cell-Penetrating Peptides Escape
the Endosome by Inducing Vesicle Budding and Collapse. ACS Chem. Biol..

[64] Wimley W. C., Hristova K., Ladokhin A. S., Silvestro L., Axelsen P. H., White S. H. (1998). Folding of β-sheet
membrane
proteins: A hydrophobic hexapeptide model. J.
Mol. Biol..

[65] Ladokhin A. S., White S. H. (2001). Protein chemistry at membrane interfaces: non-additivity
of electrostatic and hydrophobic interactions. J. Mol. Biol..

[66] White S. H., Wimley W. C., Ladokhin A. S., Hristova K. (1998). Protein folding in
membranes: Determining the energetics of peptide-bilayer interactions. Methods Enzymol..

[67] Macdonald P. M., Seelig J. (1988). Anion binding to neutral and positively
charged lipid
membranes. Biochemistry..

[68] White S. H., Wimley W. C. (1999). Membrane protein
folding and stability: physical principles. Annu. Rev. Biophys. Biomol. Struct..

[69] Beschiaschvili G., Seelig J. (1992). Peptide binding to
lipid bilayers: Nonclassical hydrophobic
effect and membrane-induced pk shifts. Biochemistry..

[70] Seelig A. (1992). Interaction
of a substance P agonist and of substance P antagonists with lipid
membranes: A thermodynamic analysis. Biochemistry..

[71] Bauerle H. D., Seelig J. (1991). Interaction of charged and uncharged calcium channel
antagonists with phospholipid membranes: Binding equilibrium, binding
enthalpy, and membrane location. Biochemistry..

[72] Seelig J., Ganz P. (1991). Nonclassical hydrophobic
effect in membrane binding equilibria. Biochemistry..

[73] Kuchinka E., Seelig J. (1989). Interaction of melittin with phosphatidylcholine membranes.
Binding isotherm and lipid head-group conformation. Biochemistry..

[74] Seelig A., Macdonald P. M. (1989). Binding of a neuropeptide, substance P, to neutral
and negatively charged lipids. Biochemistry..

[75] Beschiaschvili G., Seelig J. (1990). Melittin binding to
mixed phosphatidylglycerol phosphatidylcholine
membranes. Biochemistry..

[76] Kyrychenko A., Freites J. A., He J., Tobias D. J., Wimley W. C., Ladokhin A. S. (2014). Structural plasticity
in the topology of the membrane-interacting
domain of HIV-1 gp41. Biophys. J..

[77] Franks F. (1965). Hydrophobic
hydration and the effect of hydrogen bonding solutes on the structure
of water. Ann. N.Y. Acad. Sci..

[78] Ojemalm K., Higuchi T., Jiang Y., Langel U., Nilsson I., White S. H., Suga H., von Heijne G. (2011). Apolar surface
area determines the efficiency of translocon-mediated membrane-protein
integration into the endoplasmic reticulum. Proc. Natl. Acad. Sci. U. S. A..

[79] Moon C. P., Fleming K. G. (2011). Side-chain hydrophobicity
scale derived from transmembrane
protein folding into lipid bilayers. Proc. Natl.
Acad. Sci. U. S. A..

[80] Fauchère J.-L., Pliska V. (1983). Hydrophobic parameters
π of amino-acid side chains
from the partitioning of N-acetyl-amino-acid amides. Eur. J. Med. Chem. -Chim Ther..

[81] Radzicka A., Wolfenden R. (1988). Comparing the polarities of the amino acids: Side-chain
distribution coefficients between the vapor phase, cyclohexane, 1-octanol,
and neutral aqueous solution. Biochemistry..

[82] Engelman D. M., Steitz T. A., Goldman A. (1986). Identifying nonpolar transbilayer
helices in amino acid sequences of membrane proteins. Annu. Rev. Biophys. Biophys. Chem..

[83] Wimley W. C., Creamer T. P., White S. H. (1996). Solvation
energies of amino acid
sidechains and backbone in a family of host-guest pentapeptides. Biochemistry..

[84] Wimley W. C., White S. H. (1996). Experimentally determined hydrophobicity scale for
proteins at membrane interfaces. Nat. Struct.
Biol..

[85] Hessa T., Meindl-Beinker N. M., Bernsel A., Kim H., Sato Y., Lerch-Bader M., Nilsson I., White S. H., von Heijne G. (2007). Molecular
code for transmembrane-helix recognition by the Sec61 translocon. Nature..

[86] Lorent J. H., Levental K. R., Ganesan L., Rivera-Longsworth G., Sezgin E., Doktorova M. D., Lyman E., Levental I. (2020). Plasma membranes
are asymmetric in lipid unsaturation, packing and protein shape. Nature Chemical Biology..

[87] Lammers T. (2024). Nanomedicine
Tumor Targeting. Adv. Mater..

[88] Fan D., Cao Y., Cao M., Wang Y., Cao Y., Gong T. (2023). Nanomedicine
in cancer therapy. Signal Transduct Target Ther..

[89] Ingolfsson H. I., Melo M. N., van Eerden F. J., Arnarez C., Lopez C. A., Wassenaar T. A., Periole X., de Vries A. H., Tieleman D. P., Marrink S. J. (2014). Lipid organization
of the plasma membrane. J. Am. Chem. Soc..

[90] Sonnino S., Mauri L., Chigorno V., Prinetti A. (2007). Gangliosides as components
of lipid membrane domains. Glycobiology..

[91] Sonnino S., Chiricozzi E., Grassi S., Mauri L., Prioni S., Prinetti A. (2018). Gangliosides in Membrane Organization. Prog. Mol. Biol. Transl Sci..

[92] Ingolfsson H. I., Carpenter T. S., Bhatia H., Bremer P. T., Marrink S. J., Lightstone F. C. (2017). Computational
Lipidomics of the Neuronal Plasma Membrane. Biophys. J..

[93] Diaz J., Pellois J. P. (2023). Deciphering variations in the endocytic
uptake of a
cell-penetrating peptide: the crucial role of cell culture protocols. Cytotechnology..

[94] Brock D. J., Kondow-McConaghy H., Allen J., Brkljaca Z., Kustigian L., Jiang M., Zhang J., Rye H., Vazdar M., Pellois J. P. (2020). Mechanism of Cell Penetration by Permeabilization of
Late Endosomes: Interplay between a Multivalent TAT Peptide and Bis­(monoacylglycero)­phosphate.
Cell. Chem. Biol..

[95] Muthukrishnan N., Johnson G. A., Erazo-Oliveras A., Pellois J. P. (2013). Synergy between
cell-penetrating peptides and singlet oxygen generators leads to efficient
photolysis of membranes. Photochem. Photobiol..

[96] Erazo-Oliveras A., Muthukrishnan N., Baker R., Wang T. Y., Pellois J. P. (2012). Improving
the Endosomal Escape of Cell-Penetrating Peptides and Their Cargos:
Strategies and Challenges. Pharmaceuticals..

[97] Fretz M. M., Penning N. A., Al-Taei S., Futaki S., Takeuchi T., Nakase I., Storm G., Jones A. T. (2007). Temperature-, concentration-
and cholesterol-dependent translocation of L- and D-octa-arginine
across the plasma and nuclear membrane of CD34+ leukaemia cells. Biochem. J..

[98] Melikov K., Hara A., Yamoah K., Zaitseva E., Zaitsev E., Chernomordik L. (2015). Efficient entry of cell-penetrating peptide nona-arginine
into adherent cells involves a transient increase in intracellular
calcium. Biochem. J..

[99] Mishra A., Lai G. H., Schmidt N. W., Sun V. Z., Rodriguez aR, Tong R., Tang L., Cheng J., Deming T. J., Kamei D. T., Wong G. C. L. (2011). Translocation of HIV TAT peptide
and analogues induced by multiplexed membrane and cytoskeletal interactions. Proceedings of the National Academy of Sciences..

[100] Schmidt N. W., Wong G. C. (2013). Antimicrobial peptides and induced
membrane curvature: geometry, coordination chemistry, and molecular
engineering. Curr. Opin Solid State Mater. Sci..

[101] Schmidt N. W., Mishra A., Wang J., DeGrado W. F., Wong G. C. (2013). Influenza
virus A M2 protein generates negative Gaussian
membrane curvature necessary for budding and scission. J. Am. Chem. Soc..

[102] Schmidt N. W., Lis M., Zhao K., Lai G. H., Alexandrova A. N., Tew G. N., Wong G. C. (2012). Molecular
basis
for nanoscopic membrane curvature generation from quantum mechanical
models and synthetic transporter sequences. J. Am. Chem. Soc..

[103] Kaiser E. T., Kezdy F. J. (1983). Secondary structures
of proteins
and peptides in amphiphilic environments (A review). Proc. Natl. Acad. Sci. U. S. A..

[104] Almeida P. F., Ladokhin A. S., White S. H. (2012). Hydrogen-bond
energetics
drive helix formation in membrane interfaces. Biochim. Biophys. Acta.

[105] Ladokhin A. S., White S. H. (1999). Folding of Amphipathic
α-Helices
on Membranes: Energetics of Helix Formation by Melittin. J. Mol. Biol..

[106] Hristova K., Dempsey C. E., White S. H. (2001). Structure,
location,
and lipid perturbations of melittin at the membrane interface. Biophys. J..

[107] Dupont E., Prochiantz A., Joliot A. (2011). Penetratin story: an
overview. Methods Mol. Biol..

[108] Leeson P. D., Springthorpe B. (2007). The influence
of drug-like concepts
on decision-making in medicinal chemistry. Nat.
Rev. Drug Discovery.

[109] LaRochelle J. R., Cobb G. B., Steinauer A., Rhoades E., Schepartz A. (2015). Fluorescence
Correlation Spectroscopy
Reveals Highly Efficient Cytosolic Delivery of Certain Penta-Arg Proteins
and Stapled Peptides. J. Am. Chem. Soc..

[110] Schmidt N., Mishra A., Lai G. H., Wong G. C. (2010). Arginine-rich
cell-penetrating peptides. FEBS Lett..

[111] Mitchell D. J., Kim D. T., Steinman L., Fathman C. G., Rothbard J. B. (2000). Polyarginine
enters cells more efficiently than other
polycationic homopolymers. Journal of Peptide
Research..

[112] Holowka E. P., Sun V. Z., Kamei D. T., Deming T. J. (2007). Polyarginine
segments in block copolypeptides drive both vesicular assembly and
intracellular delivery. Nat. Mater..

[113] Takeuchi T., Futaki S. (2016). Current Understanding
of Direct Translocation
of Arginine-Rich Cell-Penetrating Peptides and Its Internalization
Mechanisms. Chem. Pharm. Bull. (Tokyo)..

[114] Parsegian A. (1969). Energy of
an ion crossing a low dielectric membrane:
Solutions to four relevant electrostatic problems. Nature (London)..

[115] Dorairaj S., Allen T. W. (2007). On the thermodynamic
stability of
a charged arginine side chain in a transmembrane helix. Proc. Natl. Acad. Sci. U. S. A..

[116] Guha S., Ghimire J., Wu E., Wimley W. C. (2019). Mechanistic
Landscape of Membrane-Permeabilizing Peptides. Chem. Rev..

[117] Bechinger B. (2008). A dynamic
view of peptides and proteins in membranes. Cell. Mol. Life Sci..

[118] Wimley W. C. (2010). Describing the mechanism of antimicrobial
peptide action
with the interfacial activity model. ACS Chem.
Biol..

[119] Sani M. A., Separovic F. (2016). How Membrane-Active Peptides Get
into Lipid Membranes. Acc. Chem. Res..

[120] Bechinger B., Lohner K. (2006). Detergent-like actions of linear
amphipathic cationic antimicrobial peptides. Biochim. Biophys. Acta.

[121] Cruz J., Mihailescu M., Wiedman G., Herman K., Searson P. C., Wimley W. C., Hristova K. (2013). A membrane translocating
peptide penetrates into bilayers without significant bilayer perturbations. Biophys. J..

[122] Henriques S. T., Castanho M. A. (2008). Translocation or membrane disintegration?
Implication of peptide-membrane interactions in pep-1 activity. J. Pept Sci..

[123] Henriques S. T., Castanho M. A., Pattenden L. K., Aguilar M. I. (2010). Fast membrane association
is a crucial factor in the
peptide pep-1 translocation mechanism: a kinetic study followed by
surface plasmon resonance. Biopolymers..

[124] Benfield A. H., Defaus S., Lawrence N., Chaousis S., Condon N., Cheneval O., Huang Y. H., Chan L. Y., Andreu D., Craik D. J., Henriques S. T. (2021). Cyclic
gomesin, a stable redesigned spider peptide able to enter cancer cells. Biochim Biophys Acta Biomembr..

[125] He J., Kauffman W. B., Fuselier T., Naveen S. K., Voss T. G., Hristova K., Wimley W. C. (2013). Direct
Cytosolic Delivery of Polar
Cargo to Cells by Spontaneous Membrane-translocating Peptides. J. Biol. Chem..

[126] Fuselier T., Wimley W. C. (2017). Spontaneous Membrane
Translocating
Peptides: The Role of Leucine-Arginine Consensus Motifs. Biophys. J..

[127] Ferrie R. P., Fuselier T., Wimley W. C. (2024). Cytosolic Delivery
of Bioactive Cyclic Peptide Cargo by Spontaneous Membrane Translocating
Peptides. ACS. Omega..

[128] Di Pisa M., Chassaing G., Swiecicki Jm. (2015). Translocation
Mechanism(s) of Cell-Penetrating Peptides: Biophysical Studies Using
Artificial Membrane Bilayers. Biochemistry..

[129] Swiecicki J.-M., Di Pisa M., Burlina F., Lecorche P., Mansuy C., Chassaing G., Lavielle S. (2015). Accumulation of Cell-Penetrating
Peptides in Large Unilamellar Vesicles - A straightforward Screening
Assay for Investigating the Internalization Mechanism. Biopolymers..

[130] Di Pisa M.
D., Chassaing G., Swiecicki Jm. (2015). When cationic
cell-penetrating peptides meet hydrocarbons to enhance in-cell cargo
delivery. Journal of Peptide Science..

[131] Schneider A. F. L., Kithil M., Cardoso M. C., Lehmann M., Hackenberger C. P. R. (2021). Cellular uptake of large biomolecules
enabled by cell-surface-reactive
cell-penetrating peptide additives. Nat. Chem..

[132] Hu Y., Patel S. (2015). Structural
and Thermodynamic Insight into Spontaneous
Membrane-Translocating Peptides Across Model PC/PG Lipid Bilayers. Journal of membrane biology..

[133] Tiriveedhi V., Miller M., Butko P., Li M. (2012). Autonomous
transmembrane segment S4 of the voltage sensor domain partitions into
the lipid membrane. Biochim. Biophys. Acta.

[134] Wee C. L., Chetwynd A., Sansom M. S. (2011). Membrane insertion
of a voltage sensor helix. Biophys. J..

[135] Freites J. A., Tobias D. J., White S. H. (2006). A voltage-sensor
water pore. Biophys. J..

[136] Hong M., Su Y. (2011). Structure and dynamics
of cationic
membrane peptides and proteins: insights from solid-state NMR. Protein Sci..

[137] Rothbard J. B., Jessop T. C., Wender P. (2005). Adaptive translocation:
The role of hydrogen bonding and membrane potential in the uptake
of guanidinium-rich transporters into cells. Adv. Drug Delivery Rev..

[138] Herce H. D., Garcia A. E., Cardoso M. C. (2014). Fundamental Molecular
Mechanism for the Cellular Uptake of Guanidinium-Rich Molecules. J. Am. Chem. Soc..

[139] Katayama S., Nakase I., Yano Y., Murayama T., Nakata Y., Matsuzaki K., Futaki S. (2013). Effects of pyrenebutyrate
on the translocation of arginine-rich cell-penetrating peptides through
artificial membranes: Recruiting peptides to the membranes, dissipating
liquid-ordered phases, and inducing curvature. Biochim. Biophys. Acta.

[140] Yoneyama F., Imura Y., Ohno K., Zendo T., Nakayama J., Matsuzaki K., Sonomoto K. (2009). Peptide-lipid huge
toroidal pore, a new antimicrobial mechanism mediated by a lactococcal
bacteriocin, lacticin Q. Antimicrob. Agents
Chemother..

[141] Bertelsen M., Lacey M. M., Nichol T., Miller K. (2023). Mechanistic
Insight into the Early Stages of Toroidal Pore Formation by the Antimicrobial
Peptide Smp24. Pharmaceutics..

[142] Yang L., Harroun T. A., Weiss T. M., Ding L., Huang H. W. (2001). Barrel-stave model or toroidal model?
A case study
on melittin pores. Biophys. J..

[143] Swiecicki Jm, Bartsch A., Tailhades J., Di Pisa M., Heller B., Chassaing G., Mansuy C., Burlina F., Lavielle S. (2014). The efficacies
of cell-penetrating
peptides in accumulating in large unilamellar vesicles depend on their
ability to form inverted micelles. ChemBioChem..

[144] Choi D., Moon J. H., Kim H., Sung B. J., Kim M. W., Tae G. Y., Satija S. K., Akgun B., Yu C. J., Lee H. W., Lee D. R., Henderson J. M., Kwong J. W., Lam K. L., Lee K. Y. C., Shin K. (2012). Insertion
mechanism of cell-penetrating peptides into supported phospholipid
membranes revealed by X-ray and neutron reflection. Soft Matter..

[145] Lamaziere A., Maniti O., Wolf C., Lambert O., Chassaing G., Trugnan G., Ayala-Sanmartin J. (2010). Lipid domain
separation, bilayer thickening and pearling induced by the cell penetrating
peptide penetratin. Biochimica et Biophysica
Acta - Biomembranes..

[146] Joanne P., Galanth C., Goasdoue N., Nicolas P., Sagan S., Lavielle S., Chassaing G., El Amri C., Alves I. D. (2009). Lipid reorganization induced by membrane-active
peptides probed using differential scanning calorimetry. Biochimica et Biophysica Acta - Biomembranes..

[147] Cardoso A. M., Trabulo S., Cardoso A. L., Lorents A., Morais C. M., Gomes P., Nunes C., Lucio M., Reis S., Padari K., Pooga M., Pedroso
de Lima M. C., Jurado A. S. (2012). S4­(13)-PV cell-penetrating peptide
induces physical and morphological changes in membrane-mimetic lipid
systems and cell membranes: implications for cell internalization. Biochim. Biophys. Acta.

[148] Herce H. D., Garcia A. E. (2007). Molecular dynamics
simulations suggest
a mechanism for translocation of the HIV-1 TAT peptide across lipid
membranes. Proceedings of the National Academy
of Sciences of the United States of America..

[149] Lamaziere A., Burlina F., Wolf C., Chassaing G., Trugnan G., Ayala-Sanmartin J. (2007). Non-metabolic
membrane tubulation
and permeability induced by bioactive peptides. PLoS One..

[150] Kwon B., Waring A. J., Hong M. (2013). A 2H Solid-state nmr
study of lipid clustering by cationic antimicrobial and Cell-penetrating
peptides in model bacterial membranes. Biophys.
J..

[151] Wadhwani P., Reichert J., Burck J., Ulrich A. S. (2012). Antimicrobial
and cell-penetrating peptides induce lipid vesicle fusion by folding
and aggregation. European Biophysics Journal..

[152] Kozlov M. M., Campelo F., Liska N., Chernomordik L. V., Marrink S. J., McMahon H. T. (2014). Mechanisms shaping
cell membranes. Curr. Opin Cell Biol..

[153] Redpath G. M. I., Betzler V. M., Rossatti P., Rossy J. (2020). Membrane Heterogeneity
Controls Cellular Endocytic Trafficking. Front
Cell Dev Biol..

[154] Akishiba M., Takeuchi T., Kawaguchi Y., Sakamoto K., Yu H. H., Nakase I., Takatani-Nakase T., Madani F., Graslund A., Futaki S. (2017). Cytosolic antibody
delivery by lipid-sensitive endosomolytic peptide. Nat. Chem..

[155] Hirose H., Takeuchi T., Osakada H., Pujals S., Katayama S., Nakase I., Kobayashi S., Haraguchi T., Futaki S. (2012). Transient focal membrane deformation
induced by arginine-rich peptides leads to their direct penetration
into cells. Mol. Ther..

[156] Teo S. L. Y., Rennick J. J., Yuen D., Al-Wassiti H., Johnston A. P. R., Pouton C. W. (2021). Unravelling cytosolic
delivery of
cell penetrating peptides with a quantitative endosomal escape assay. Nat. Commun..

[157] Lucchino M., Billet A., Bai S. K., Dransart E., Hadjerci J., Schmidt F., Wunder C., Johannes L. (2021). Absolute Quantification
of Drug Vector Delivery to the Cytosol. Angew.
Chem., Int. Ed. Engl..

[158] Bus T., Traeger A., Schubert U. S. (2018). The great
escape: how cationic polyplexes
overcome the endosomal barrier. J. Mater. Chem.
B.

[159] Moulay G., Leborgne C., Mason A. J., Aisenbrey C., Kichler A., Bechinger B. (2017). Histidine-rich designer peptides
of the LAH4 family promote cell delivery of a multitude of cargo. J. Pept Sci..

[160] Selby L. I., Cortez-Jugo C. M., Such G. K., Johnston A. P. R. (2017). Nanoescapology:
progress toward understanding the endosomal escape of polymeric nanoparticles. Wiley Interdiscip Rev. Nanomed Nanobiotechnol..

[161] Lonn P., Kacsinta A. D., Cui X. S., Hamil A. S., Kaulich M., Gogoi K., Dowdy S. F. (2016). Enhancing
Endosomal
Escape for Intracellular Delivery of Macromolecular Biologic Therapeutics. Sci. Rep..

[162] Mayer L. D., Hope M. J., Cullis P. R. (1986). Vesicles
of variable
sizes produced by a rapid extrusion procedure. Biochim. Biophys. Acta.

[163] Hope M. J., Bally M. B., Mayer L. D., Janoff A. S., Cullis P. R. (1986). Generation of multilamellar and unilamellar phospholipid
vesicles. Chem. Phys. Lipids..

[164] Troeira Henriques S., Nuno Melo M., Castanho M. A. R. B. (2007). How to address
CPP and AMP translocation? Methods to detect and quantify peptide
internalization in vitro and in vivo (Review). Mol. Membr. Biol..

[165] Matsuzaki K., Yoneyama S., Miyajima K. (1997). Pore formation and
translocation of melittin. Biophys. J..

[166] Spinella S., Nelson R. B., Elmore D. E. (2012). Measuring peptide
translocation into large unilamellar vesicles. Journal of visualized experiments: JoVE.

[167] Walrant A., Matheron L., Cribier S., Chaignepain S., Jobin M. L., Sagan S., Alves I. D. (2013). Direct translocation
of cell-penetrating peptides in liposomes: A combined mass spectrometry
quantification and fluorescence detection study. Anal. Biochem..

[168] Terrone D., Sang S. L. W., Roudaia L., Silvius J. R. (2003). Penetratin
and Related Cell-Penetrating Cationic Peptides Can Translocate Across
Lipid Bilayers in the Presence of a Transbilayer Potential. Biochemistry..

[169] Reeves J. P., Dowben R. M. (1969). Formation and properties
of thin-walled
phospholipid vesicles. J. Cell Physiol..

[170] Angelova M. I., Dimitrov D. S. (1986). Liposome electroformation. Faraday Discuss. Chem. Soc..

[171] Ambroggio E. E., Separovic F., Bowie J. H., Fidelio G. D., Bagatolli L. A. (2005). Direct visualization of membrane leakage induced by
the antibiotic peptides: maculatin, citropin, and aurein. Biophys. J..

[172] Islam M. Z., Alam J. M., Tamba Y., Karal M. A. S., Yamazaki M. (2014). The single GUV method for revealing the functions of
antimicrobial, pore-forming toxin, and cell-penetrating peptides or
proteins. Physical chemistry chemical physics:
PCCP..

[173] Islam M. Z., Ariyama H., Alam J. M., Yamazaki M. (2014). Entry of cell-penetrating
peptide transportan 10 into a single vesicle by translocating across
lipid membrane and its induced pores. Biochemistry..

[174] Bangham A.
D., Standish M. M., Watkins J. C. (1965). Diffusion of univalent
ions across the lamellae of swollen phospholipids. J. Mol. Biol..

[175] Kansy M., Senner F., Gubernator K. (1998). Physicochemical
high throughput screening: parallel artificial membrane permeation
assay in the description of passive absorption processes. J. Med. Chem..

[176] Campbell S. D., Regina K. J., Kharasch E. D. (2014). Significance
of
lipid composition in a blood-brain barrier-mimetic PAMPA assay. J. Biomol Screen..

[177] Ano R., Kimura Y., Shima M., Matsuno R., Ueno T., Akamatsu M. (2004). Relationships between
structure and high-throughput
screening permeability of peptide derivatives and related compounds
with artificial membranes: application to prediction of Caco-2 cell
permeability. Bioorg. Med. Chem..

[178] Malakoutikhah M., Prades R., Teixido M., Giralt E. (2010). N-methyl phenylalanine-rich
peptides as highly versatile blood-brain barrier shuttles. J. Med. Chem..

[179] Marelli U. K., Ovadia O., Frank A. O., Chatterjee J., Gilon C., Hoffman A., Kessler H. (2015). cis-Peptide
Bonds:
A Key for Intestinal Permeability of Peptides?. Chemistry..

[180] Marelli U. K., Bezencon J., Puig E., Ernst B., Kessler H. (2015). Enantiomeric cyclic peptides with different Caco-2
permeability suggest carrier-mediated transport. Chemistry..

[181] Morimoto J., Amano R., Ono T., Sando S. (2019). A parallel
permeability assay of peptides across artificial membranes and cell
monolayers using a fluorogenic reaction. Org.
Biomol Chem..

[182] Truszkowska M., Ebert M. L., Afzal K. B., Gyorgyi V., Bernkop-Schnurch A. (2025). Peptide drug
delivery: Permeation behaviour and intracellular
fate of hydrophobic ion pairs in self-emulsifying drug delivery systems. Eur. J. Pharm. Sci..

[183] Sarabipour S., Chan R. B., Zhou B., Di Paolo G., Hristova K. (2015). Analytical characterization of plasma membrane-derived
vesicles produced via osmotic and chemical vesiculation. Biochim. Biophys. Acta.

[184] Gentry S. B., Nowak S. J., Ni X., Hill S. A., Wade L. R., Clark W. R., Keelaghan A. P., Morris D. P., McMurry J. L. (2021). A real-time assay for cell-penetrating
peptide-mediated delivery of molecular cargos. PLoS One..

[185] Rotem R., Bertolini J. A., Salvioni L., Barbieri L., Rizzuto M. A., Tinelli V., Gori A., Adams S., Colombo M., Prosperi D. (2023). Direct quantification of cytosolic
delivery of drug nanocarriers using FlAsH-EDT2. Nanomedicine..

[186] Elliott A. D. (2020). Confocal Microscopy: Principles and Modern Practices. Curr. Protoc Cytom..

[187] Jarver P., Langel U. (2006). Cell-penetrating peptides--a
brief
introduction. Biochim. Biophys. Acta.

[188] Madani F., Lindberg S., Langel U., Futaki S., Graslund A. (2011). Mechanisms
of cellular uptake of cell-penetrating peptides. Journal of Biophysics..

[189] Richard J. P., Melikov K., Vives E., Ramos C., Verbeure B., Gait M. J., Chernomordik L. V., Lebleu B. (2003). Cell-penetrating peptides:
A reevaluation of the mechanism
of cellular uptake. J. Biol. Chem..

[190] Chen J., Li H., Chen J. (2017). Human epidermal growth
factor coupled to different structural classes of cell penetrating
peptides: A comparative study. Int. J. Biol.
Macromol..

[191] Wu E., Ellis A., Bell K., Moss D. L., Landry S. J., Hristova K., Wimley W. C. (2024). pH-Responsive Peptide Nanoparticles
Deliver Macromolecules to Cells via Endosomal Membrane Nanoporation. ACS Nano.

[192] Macchi S., Signore G., Boccardi C., Di Rienzo C., Beltram F., Cardarelli F. (2015). Spontaneous
membrane-translocating
peptides: influence of peptide self-aggregation and cargo polarity. Sci. Rep..

[193] Lattig-Tunnemann G., Prinz M., Hoffmann D., Behlke J., Palm-Apergi C., Morano I., Herce H. D., Cardoso M. C. (2011). Backbone
rigidity and static presentation of guanidinium groups increases cellular
uptake of arginine-rich cell-penetrating peptides. Nat. Commun..

[194] Thoren P. E., Persson D., Isakson P., Goksor M., Onfelt A., Norden B. (2003). Uptake of analogs of
penetratin,
Tat(48–60) and oligoarginine in live cells. Biochem. Biophys. Res. Commun..

[195] Lavis L. D., Raines R. T. (2008). Bright ideas for
chemical biology. ACS Chem. Biol..

[196] Specht E. A., Braselmann E., Palmer A. E. (2017). A Critical and Comparative
Review of Fluorescent Tools for Live-Cell Imaging. Annu. Rev. Physiol..

[197] Lavis L. D., Raines R. T. (2014). Bright building
blocks for chemical
biology. ACS Chem. Biol..

[198] Wysocki L. M., Lavis L. D. (2011). Advances in the
chemistry of small
molecule fluorescent probes. Curr. Opin Chem.
Biol..

[199] Komin A., Bogorad M. I., Lin R., Cui H., Searson P. C., Hristova K. (2020). A peptide for transcellular cargo
delivery: Structure-function relationship and mechanism of action. J. Controlled Release.

[200] Salomone F., Cardarelli F., Di Luca M., Boccardi C., Nifosi R., Bardi G., Di Bari L., Serresi M., Beltram F. (2012). A novel chimeric cell-penetrating peptide with membrane-disruptive
properties for efficient endosomal escape. J.
Controlled Release.

[201] Costantini L. M., Baloban M., Markwardt M. L., Rizzo M. A., Guo F., Verkhusha V. V., Snapp E. L. (2015). A palette of fluorescent proteins optimized for diverse
cellular environments. Nat. Commun..

[202] Shaner N. C., Patterson G. H., Davidson M. W. (2007). Advances in fluorescent
protein technology. J. Cell Sci..

[203] Giepmans B. N., Adams S. R., Ellisman M. H., Tsien R. Y. (2006). The fluorescent
toolbox for assessing protein location and function. Science..

[204] Hedegaard S. F., Derbas M. S., Lind T. K., Kasimova M. R., Christensen M. V., Michaelsen M. H., Campbell R. A., Jorgensen L., Franzyk H., Cardenas M., Nielsen H. M. (2018). Fluorophore labeling
of a cell-penetrating peptide significantly alters the mode and degree
of biomembrane interaction. Sci. Rep..

[205] Kuzichkina E. O., Shilova O. N., Deyev S. M. (2018). The Mechanism
of
Fluorescence Quenching of Protein Photosensitizers Based on miniSOG
During Internalization of the HER2 Receptor. Acta Naturae..

[206] Cole R. (2014). Live-cell imaging. Cell Adh Migr..

[207] Jonkman J., Brown C. M., Wright G. D., Anderson K. I., North A. J. (2020). Tutorial: guidance for quantitative
confocal microscopy. Nat. Protoc..

[208] Frigault M. M., Lacoste J., Swift J. L., Brown C. M. (2009). Live-cell
microscopy - tips and tools. J. Cell Sci..

[209] Hoffmann A. R., Guha S., Wu E., Ghimire J., Wang Y., He J., Garry R. F., Wimley W. C. (2020). Broad-Spectrum
Antiviral Entry Inhibition by Interfacially-Active Peptides. J. Virol..

[210] Aditya V., Tambe V., Yue W. (2025). Development of a Novel
Automated Workflow in Fiji ImageJ for Batch Analysis of Confocal Imaging
Data to Quantify Protein Colocalization Using Manders Coefficient. Bio Protoc..

[211] Nakase I., Noguchi K., Aoki A., Takatani-Nakase T., Fujii I., Futaki S. (2017). Arginine-rich cell-penetrating peptide-modified
extracellular vesicles for active macropinocytosis induction and efficient
intracellular delivery. Sci. Rep..

[212] Peier A., Ge L., Boyer N., Frost J., Duggal R., Biswas K., Edmondson S., Hermes J. D., Yan L., Zimprich C., Sadruddin A., Kristal Kaan H. Y., Chandramohan A., Brown C. J., Thean D., Lee X. E., Yuen T. Y., Ferrer-Gago F. J., Johannes C. W., Lane D. P., Sherborne B., Corona C., Robers M. B., Sawyer T. K., Partridge A. W. (2021). NanoClick:
A High Throughput, Target-Agnostic Peptide Cell Permeability Assay. ACS Chem. Biol..

[213] Stanzl E. G., Trantow B. M., Vargas J. R., Wender P. (2013). Fifteen years
of cell-penetrating, guanidinium-rich molecular transporters: Basic
science, research tools, and clinical applications. Acc. Chem. Res..

[214] Wender P., Galliher W. C., Goun E., Jones L. R., Pillow T. H. (2008). The design of guanidinium-rich transporters
and their
internalization mechanisms. Adv. Drug Delivery
Rev..

[215] Rothbard J. B., Kreider E., VanDeusen C. L., Wright L., Wylie B. L., Wender P. (2002). Arginine-rich molecular
transporters for drug delivery: Role of backbone spacing in cellular
uptake. J. Med. Chem..

[216] Koren E., Torchilin V. P. (2012). Cell-penetrating
peptides: breaking
through to the other side. Trends in molecular
medicine..

[217] Crowley L. C., Scott A. P., Marfell B. J., Boughaba J. A., Chojnowski G., Waterhouse N. J. (2016). Measuring Cell Death by Propidium
Iodide Uptake and Flow Cytometry. Cold Spring
Harb Protoc..

[218] Fadok V. A., Voelker D. R., Campbell P. A., Cohen J. J., Bratton D. L., Henson P. M. (1992). Exposure of phosphatidylserine on
the surface of apoptotic lymphocytes triggers specific recognition
and removal by macrophages. J. Immunol..

[219] Klymchenko A. S., Kreder R. (2014). Fluorescent probes for lipid rafts:
from model membranes to living cells. Chem.
Biol..

[220] Dodes Traian M. M., Flecha F. L. G., Levi V. (2012). Imaging lipid
lateral
organization in membranes with C-laurdan in a confocal microscope. J. Lipid Res..

[221] Henriques S. T., Costa J., Castanho M. A. (2005). Translocation
of
beta-galactosidase mediated by the cell-penetrating peptide pep-1
into lipid vesicles and human HeLa cells is driven by membrane electrostatic
potential. Biochemistry..

[222] Hallbrink M., Floren A., Elmquist A., Pooga M., Bartfai T., Langel Ü (2001). Cargo delivery
kinetics of cell-penetrating
peptides. Biochimica et Biophysica Acta - Biomembranes..

[223] Nischan N., Herce H. D., Natale F., Bohlke N., Budisa N., Cardoso M. C., Hackenberger C. P. (2015). Covalent
attachment of cyclic TAT peptides to GFP results in protein delivery
into live cells with immediate bioavailability. Angew. Chem., Int. Ed. Engl..

[224] Milech N., Longville B. A., Cunningham P. T., Scobie M. N., Bogdawa H. M., Winslow S., Anastasas M., Connor T., Ong F., Stone S. R., Kerfoot M., Heinrich T., Kroeger K. M., Tan Y. F., Hoffmann K., Thomas W. R., Watt P. M., Hopkins R. M. (2015). GFP-complementation
assay to detect functional CPP and protein delivery into living cells. Sci. Rep..

[225] Beddoes C. M., Case C. P., Briscoe W. H. (2015). Understanding
nanoparticle
cellular entry: A physicochemical perspective. Adv. Colloid Interface Sci..

[226] Varkouhi A. K., Scholte M., Storm G., Haisma H. J. (2011). Endosomal
escape pathways for delivery of biologicals. J. Controlled Release.

[227] Hillaireau H., Couvreur P. (2009). Nanocarriers’
entry into the
cell: relevance to drug delivery. Cell. Mol.
Life Sci..

[228] Deprey K., Becker L., Kritzer J., Pluckthun A. (2019). Trapped! A
Critical Evaluation of Methods for Measuring Total Cellular Uptake
versus Cytosolic Localization. Bioconjug Chem..

[229] Foillard S., Jin Z. H., Garanger E., Boturyn D., Favrot M. C., Coll J. L., Dumy P. (2008). Synthesis and biological
characterisation of targeted pro-apoptotic peptide. Chembiochem..

[230] Ellerby H. M., Arap W., Ellerby L. M., Kain R., Andrusiak R., Rio G. D., Krajewski S., Lombardo C. R., Rao R., Ruoslahti E., Bredesen D. E., Pasqualini R. (1999). Anti-cancer activity of targeted
pro-apoptotic peptides. Nat. Med..

[231] Marks A. J., Cooper M. S., Anderson R. J., Orchard K. H., Hale G., North J. M., Ganeshaguru K., Steele A. J., Mehta A. B., Lowdell M. W., Wickremasinghe R. G. (2005). Selective
apoptotic killing of malignant hemopoietic cells by antibody-targeted
delivery of an amphipathic peptide. Cancer Res..

[232] Oehlke J., Lorenz D., Wiesner B., Bienert M. (2005). Studies on
the cellular uptake of substance P and lysine-rich, KLA-derived model
peptides. J. Mol. Recognit..

[233] Montrose K., Yang Y., Sun X., Wiles S., Krissansen G. W. (2013). Xentry, a new class of cell-penetrating
peptide uniquely
equipped for delivery of drugs. Sci. Rep..

[234] Han I. H., Jeong C., Yang J., Park S. H., Hwang D. S., Bae H. (2022). Therapeutic Effect
of Melittin-dKLA
Targeting Tumor-Associated Macrophages in Melanoma. Int. J. Mol. Sci..

[235] Walensky L. D., Kung A. L., Escher I., Malia T. J., Barbuto S., Wright R. D., Wagner G., Verdine G. L., Korsmeyer S. J. (2004). Activation of apoptosis in vivo by a hydrocarbon-stapled
BH3 helix. Science..

[236] Walensky L. D., Pitter K., Morash J., Oh K. J., Barbuto S., Fisher J., Smith E., Verdine G. L., Korsmeyer S. J. (2006). A stapled BID BH3 helix directly
binds and activates
BAX. Mol. Cell.

[237] An M., Wijesinghe D., Andreev O. A., Reshetnyak Y. K., Engelman D. M. (2010). pH-(low)-insertion-peptide
(pHLIP) translocation of
membrane impermeable phalloidin toxin inhibits cancer cell proliferation. Proc. Natl. Acad. Sci. U. S. A..

[238] Moshnikova A., Moshnikova V., Andreev O. A., Reshetnyak Y. K. (2013). Antiproliferative
effect of pHLIP-amanitin. Biochemistry..

[239] Reshetnyak Y. K., Andreev O. A., Engelman D. M. (2024). Aiming the magic
bullet: targeted delivery of imaging and therapeutic agents to solid
tumors by pHLIP peptides. Front Pharmacol..

[240] Thevenin D., An M., Engelman D. M. (2009). pHLIP-mediated
translocation
of membrane-impermeable molecules into cells. Chem. Biol..

[241] Michalska M., Wolf P. (2015). Pseudomonas Exotoxin
A: optimized
by evolution for effective killing. Front Microbiol..

[242] Deng Q., Barbieri J. T. (2008). Molecular mechanisms
of the cytotoxicity
of ADP-ribosylating toxins. Annu. Rev. Microbiol..

[243] Dixon A. S., Schwinn M. K., Hall M. P., Zimmerman K., Otto P., Lubben T. H., Butler B. L., Binkowski B. F., Machleidt T., Kirkland T. A., Wood M. G., Eggers C. T., Encell L. P., Wood K. V. (2016). NanoLuc Complementation
Reporter
Optimized for Accurate Measurement of Protein Interactions in Cells. ACS Chem. Biol..

[244] Hoare B. L., Kocan M., Bruell S., Scott D. J., Bathgate R. A. D. (2019). Using the novel HiBiT tag to label
cell surface relaxin
receptors for BRET proximity analysis. Pharmacol
Res. Perspect..

[245] Schwinn M. K., Steffen L. S., Zimmerman K., Wood K. V., Machleidt T. (2020). A Simple and
Scalable Strategy for
Analysis of Endogenous Protein Dynamics. Sci.
Rep..

[246] Amblard I., Dupont E., Alves I., Miralves J., Queguiner I., Joliot A. (2020). Bidirectional transfer
of homeoprotein
EN2 across the plasma membrane requires PIP(2). J. Cell Sci..

[247] Cabantous S., Terwilliger T. C., Waldo G. S. (2005). Protein tagging
and detection with engineered self-assembling fragments of green fluorescent
protein. Nat. Biotechnol..

[248] Cabantous S., Waldo G. S. (2006). In vivo and in vitro
protein solubility
assays using split GFP. Nat. Methods..

[249] Kim J. S., Choi D. K., Park S. W., Shin S. M., Bae J., Kim D. M., Yoo T. H., Kim Y. S. (2015). Quantitative assessment
of cellular uptake and cytosolic access of antibody in living cells
by an enhanced split GFP complementation assay. Biochem. Biophys. Res. Commun..

[250] Schmidt S., Adjobo-Hermans M. J., Wallbrecher R., Verdurmen W. P., Bovee-Geurts P. H., van Oostrum J., Milletti F., Enderle T., Brock R. (2015). Detecting
Cytosolic
Peptide Delivery with the GFP Complementation Assay in the Low Micromolar
Range. Angew. Chem., Int. Ed. Engl..

[251] Kang S. H., Cho M. J., Kole R. (1998). Up-regulation of luciferase
gene expression with antisense oligonucleotides: implications and
applications in functional assay development. Biochemistry..

[252] Asseline U., Goncalves C., Pichon C., Midoux P. (2014). Improved nuclear
delivery of antisense 2’-Ome RNA by conjugation with the histidine-rich
peptide H5WYG. journal of gene medicine..

[253] Reshetnyak Y. K., Andreev O. A., Lehnert U., Engelman D. M. (2006). Translocation
of molecules into cells by pH-dependent insertion of a transmembrane
helix. Proc. Natl. Acad. Sci. U. S. A..

[254] Nielsen P. E., Shiraishi T. (2011). Peptide nucleic acid (PNA) cell penetrating
peptide (CPP) conjugates as carriers for cellular delivery of antisense
oligomers. Artificial DNA, PNA & XNA.

[255] Abes S., Moulton H. M., Clair P., Prevot P., Youngblood D. S., Wu R. P., Iversen P. L., Lebleu B. (2006). Vectorization
of morpholino oligomers by the (R-Ahx-R)­4 peptide allows efficient
splicing correction in the absence of endosomolytic agents. J. Controlled Release.

[256] Holub J. M., Larochelle J. R., Appelbaum J. S., Schepartz A. (2013). Improved assays for determining the
cytosolic access
of peptides, proteins, and their mimetics. Biochemistry..

[257] Yu P., Liu B., Kodadek T. (2005). A high-throughput
assay for assessing
the cell permeability of combinatorial libraries. Nat. Biotechnol..

[258] Appelbaum J. S., LaRochelle J. R., Smith B. A., Balkin D. M., Holub J. M., Schepartz A. (2012). Arginine topology controls escape
of minimally cationic proteins from early endosomes to the cytoplasm. Chem. Biol..

[259] Zhang J. H., Chung T. D., Oldenburg K. R. (1999). A Simple
Statistical Parameter for Use in Evaluation and Validation of High
Throughput Screening Assays. J. Biomol Screen..

[260] Wadia J. S., Stan R. V., Dowdy S. F. (2004). Transducible TAT-HA
fusogenic peptide enhances escape of TAT-fusion proteins after lipid
raft macropinocytosis. Nat. Med..

[261] Arafiles J. V. V., Franke J., Franz L., Gomez-Gonzalez J., Kemnitz-Hassanin K., Hackenberger C. P. R. (2023). Cell-Surface-Retained
Peptide Additives
for the Cytosolic Delivery of Functional Proteins. J. Am. Chem. Soc..

[262] Yang Y. S., Hughes T. E. (2001). Cre stoplight: a
red/green fluorescent
reporter of Cre recombinase expression in living cells. Biotechniques..

[263] Shinga K., Iwata T., Murata K., Daitoku Y., Michibata J., Arafiles J. V. V., Sakamoto K., Akishiba M., Takatani-Nakase T., Mizuno S., Sugiyama F., Imanishi M., Futaki S. (2022). L17ER4: A
cell-permeable attenuated cationic amphiphilic
lytic peptide. Bioorg. Med. Chem..

[264] Peraro L., Zou Z., Makwana K. M., Cummings A. E., Ball H. L., Yu H., Lin Y. S., Levine B., Kritzer J. A. (2017). Diversity-Oriented Stapling Yields
Intrinsically Cell-Penetrant
Inducers of Autophagy. J. Am. Chem. Soc..

[265] Deprey K., Kritzer J. A. (2020). Quantitative measurement of cytosolic
penetration using the chloroalkane penetration assay. Methods Enzymol..

[266] England C. G., Luo H., Cai W. (2015). HaloTag technology:
a versatile platform for biomedical applications. Bioconjug Chem..

[267] Los G. V., Encell L. P., McDougall M. G., Hartzell D. D., Karassina N., Zimprich C., Wood M. G., Learish R., Ohana R. F., Urh M., Simpson D., Mendez J., Zimmerman K., Otto P., Vidugiris G., Zhu J., Darzins A., Klaubert D. H., Bulleit R. F., Wood K. V. (2008). HaloTag:
a novel protein labeling technology for cell imaging and protein analysis. ACS Chem. Biol..

[268] Giancola J. B., Grimm J. B., Jun J. V., Petri Y. D., Lavis L. D., Raines R. T. (2024). Evaluation of the
Cytosolic Uptake
of HaloTag Using a pH-Sensitive Dye. ACS Chem.
Biol..

[269] Machleidt T., Woodroofe C. C., Schwinn M. K., Mendez J., Robers M. B., Zimmerman K., Otto P., Daniels D. L., Kirkland T. A., Wood K. V. (2015). NanoBRET--A Novel BRET Platform for
the Analysis of Protein-Protein Interactions. ACS Chem. Biol..

[270] Devaraj N. K. (2018). The Future of Bioorthogonal Chemistry. ACS Cent Sci..

[271] Hall M. P., Unch J., Binkowski B. F., Valley M. P., Butler B. L., Wood M. G., Otto P., Zimmerman K., Vidugiris G., Machleidt T., Robers M. B., Benink H. A., Eggers C. T., Slater M. R., Meisenheimer P. L., Klaubert D. H., Fan F., Encell L. P., Wood K. V. (2012). Engineered luciferase reporter from a deep sea shrimp
utilizing a novel imidazopyrazinone substrate. ACS Chem. Biol..

[272] Juillerat A., Gronemeyer T., Keppler A., Gendreizig S., Pick H., Vogel H., Johnsson K. (2003). Directed evolution
of O6-alkylguanine-DNA alkyltransferase for efficient labeling of
fusion proteins with small molecules in vivo. Chem. Biol..

[273] Keppler A., Gendreizig S., Gronemeyer T., Pick H., Vogel H., Johnsson K. (2003). A general method for
the covalent labeling of fusion proteins with small molecules in vivo. Nat. Biotechnol..

[274] Mollwitz B., Brunk E., Schmitt S., Pojer F., Bannwarth M., Schiltz M., Rothlisberger U., Johnsson K. (2012). Directed evolution of the suicide protein O(6)-alkylguanine-DNA
alkyltransferase for increased reactivity results in an alkylated
protein with exceptional stability. Biochemistry..

[275] FitzGerald L. I., Aurelio L., Chen M., Yuen D., Rennick J. J., Graham B., Johnston A. P. R. (2020). A molecular sensor
to quantify the localization of proteins, DNA and nanoparticles in
cells. Nat. Commun..

[276] Shaner N. C., Lambert G. G., Chammas A., Ni Y., Cranfill P. J., Baird M. A., Sell B. R., Allen J. R., Day R. N., Israelsson M., Davidson M. W., Wang J. (2013). A bright monomeric
green fluorescent protein derived from Branchiostoma lanceolatum. Nat. Methods..

[277] Conradi R. A., Hilgers A. R., Ho N. F., Burton P. S. (1992). The influence
of peptide structure on transport across Caco-2 cells. II. Peptide
bond modification which results in improved permeability. Pharm. Res..

[278] Conradi R. A., Hilgers A. R., Ho N. F., Burton P. S. (1991). The influence
of peptide structure on transport across Caco-2 cells. Pharm. Res..

[279] Komin A., Russell L. M., Hristova K. A., Searson P. C. (2017). Peptide-based
strategies for enhanced cell uptake, transcellular transport, and
circulation: Mechanisms and challenges. Adv.
Drug Deliv Rev..

[280] Bednarek R. (2022). In Vitro Methods for Measuring the Permeability of
Cell Monolayers. Methods Protoc..

[281] Pardridge W. M. (2016). CSF, blood-brain barrier, and brain
drug delivery. Expert Opin Drug Delivery.

[282] Abbott N. J., Patabendige A. A., Dolman D. E., Yusof S. R., Begley D. J. (2010). Structure
and function of the blood-brain barrier. Neurobiol
Dis..

[283] Hundahl A. C., Weller A., Larsen J. B., Hjorringgaard C. U., Hansen M. B., Mundler A. K., Knuhtsen A., Kristensen K., Arnspang E. C., Andresen T. L., Mortensen K. I., Marie R. (2023). Quantitative live-cell imaging of lipidated peptide transport through
an epithelial cell layer. J. Controlled Release.

[284] Bogorad M. I., DeStefano J., Karlsson J., Wong A. D., Gerecht S., Searson P. C. (2015). Review:
in vitro microvessel models. Lab Chip..

[285] Hidalgo I. J., Raub T. J., Borchardt R. T. (1989). Characterization
of the human colon carcinoma cell line (Caco-2) as a model system
for intestinal epithelial permeability. Gastroenterology..

[286] Srinivasan B., Kolli A. R., Esch M. B., Abaci H. E., Shuler M. L., Hickman J. J. (2015). TEER measurement
techniques for in
vitro barrier model systems. J. Lab Autom..

[287] Trehin R., Nielsen H. M., Jahnke H. G., Krauss U., Beck-Sickinger A. G., Merkle H. P. (2004). Metabolic cleavage of cell-penetrating
peptides in contact with epithelial models: human calcitonin (hCT)-derived
peptides, Tat(47–57) and penetratin(43–58). Biochem. J..

[288] Palm C., Jayamanne M., Kjellander M., Hallbrink M. (2007). Peptide degradation is a critical
determinant for cell-penetrating
peptide uptake. Biochimica et Biophysica Acta-Biomembranes..

[289] Blanc E., Bonnafous C., Merida P., Cisternino S., Clair P., Scherrmann J. M., Temsamani J. (2004). Peptide-vector
strategy bypasses P-glycoprotein efflux, and enhances brain transport
and solubility of paclitaxel. Anticancer Drugs..

[290] Rousselle C., Smirnova M., Clair P., Lefauconnier J. M., Chavanieu A., Calas B., Scherrmann J. M., Temsamani J. (2001). Enhanced delivery of doxorubicin into the brain via
a peptide-vector-mediated strategy: saturation kinetics and specificity. J. Pharmacol Exp Ther..

[291] Rousselle C., Clair P., Lefauconnier J. M., Kaczorek M., Scherrmann J. M., Temsamani J. (2000). New advances
in the transport of doxorubicin through the blood-brain barrier by
a peptide vector-mediated strategy. Mol. Pharmacol..

[292] Rousselle C., Clair P., Smirnova M., Kolesnikov Y., Pasternak G. W., Gac-Breton S., Rees A. R., Scherrmann J. M., Temsamani J. (2003). Improved brain uptake and pharmacological activity
of dalargin using a peptide-vector-mediated strategy. J. Pharmacol Exp Ther..

[293] Jackman J. A., Costa V. V., Park S., Real A. L. C. V., Park J. H., Cardozo P. L., Ferhan A. R., Olmo I. G., Moreira T. P., Bambirra J. L., Queiroz V. F., Queiroz-Junior C. M., Foureaux G., Souza D. G., Ribeiro F. M., Yoon B. K., Wynendaele E., De Spiegeleer B., Teixeira M. M., Cho N.-J. (2018). Therapeutic
treatment of Zika virus infection using a brain-penetrating antiviral
peptide. Nat. Mater..

[294] Linville R. M., DeStefano J. G., Sklar M. B., Xu Z., Farrell A. M., Bogorad M. I., Chu C., Walczak P., Cheng L., Mahairaki V., Whartenby K. A., Calabresi P. A., Searson P. C. (2019). Human iPSC-derived
blood-brain barrier
microvessels: validation of barrier function and endothelial cell
behavior. Biomaterials..

[295] de la Fuente-Nunez C., Reffuveille F., Haney E. F., Straus S. K., Hancock R. E. (2014). Broad-spectrum anti-biofilm
peptide that targets a
cellular stress response. PLoS Pathog..

[296] Hamill P., Brown K., Jenssen H., Hancock R. E. (2008). Novel anti-infectives:
is host defence the answer?. Curr. Opin Biotechnol..

[297] Wiegand I., Hilpert K., Hancock R. E. (2008). Agar and broth dilution
methods to determine the minimal inhibitory concentration (MIC) of
antimicrobial substances. Nat. Protoc..

[298] Chongsiriwatana N. P., Lin J. S., Kapoor R., Wetzler M., Rea J. A. C., Didwania M. K., Contag C. H., Barron A. E. (2017). Intracellular
biomass flocculation as a key mechanism of rapid bacterial killing
by cationic, amphipathic antimicrobial peptides and peptoids. Sci. Rep..

[299] Yoneyama F., Imura Y., Ichimasa S., Fujita K., Zendo T., Nakayama J., Matsuzaki K., Sonomoto K. (2009). Lacticin Q, a lactococcal bacteriocin, causes high-level
membrane permeability in the absence of specific receptors. Appl. Environ. Microbiol..

[300] Yoneyama F., Ohno K., Imura Y., Li M., Zendo T., Nakayama J., Matsuzaki K., Sonomoto K. (2011). Lacticin Q-mediated selective toxicity depending on
physicochemical features of membrane components. Antimicrob. Agents Chemother..

[301] Benarroch J. M., Asally M. (2020). The Microbiologist’s
Guide
to Membrane Potential Dynamics. Trends Microbiol..

[302] Breukink E., Wiedemann I., Kraaij C. v., Kuipers O. P., Sahl H.-G., de Kruijff B. (1999). Use of the cell wall precursor lipid
II by a pore-forming peptide antibiotic. Science..

[303] Buttress J. A., Halte M., Te Winkel J. D., Erhardt M., Popp P. F., Strahl H. (2022). A guide for membrane
potential measurements in Gram-negative bacteria using voltage-sensitive
dyes. Microbiology (Reading)..

[304] Wenzel M., Chiriac A. I., Otto A., Zweytick D., May C., Schumacher C., Gust R., Albada H. B., Penkova M., Kramer U., Erdmann R., Metzler-Nolte N., Straus S. K., Bremer E., Becher D., Brotz-Oesterhelt H., Sahl H. G., Bandow J. E. (2014). Small cationic
antimicrobial peptides
delocalize peripheral membrane proteins. Proc.
Natl. Acad. Sci. U. S. A..

[305] Wenzel M., Kohl B., Munch D., Raatschen N., Albada H. B., Hamoen L., Metzler-Nolte N., Sahl H. G., Bandow J. E. (2012). Proteomic response of Bacillus subtilis
to lantibiotics reflects differences in interaction with the cytoplasmic
membrane. Antimicrob. Agents Chemother..

[306] Roth B. L., Poot M., Yue S. T., Millard P. J. (1997). Bacterial
viability and antibiotic susceptibility testing with SYTOX green nucleic
acid stain. Appl. Environ. Microbiol..

[307] Barns K. J., Weisshaar J. C. (2013). Real-time attack of LL-37 on single
Bacillus subtilis cells. Biochim. Biophys. Acta.

[308] Starr C. G., Maderdrut J. L., He J., Coy D. H., Wimley W. C. (2018). Pituitary
adenylate cyclase-activating polypeptide
is a potent broad-spectrum antimicrobial peptide: Structure-activity
relationships. Peptides..

[309] Imura Y., Nishida M., Matsuzaki K. (2007). Action mechanism
of PEGylated magainin 2 analogue peptide. Biochim.
Biophys. Acta.

[310] Lehrer R. I., Barton A., Daher K. A., Harwig S. S., Ganz T., Selsted M. E. (1989). Interaction of human
defensins with
Escherichia coli. Mechanism of bactericidal activity. J. Clin Invest..

[311] Azuma E., Choda N., Odaki M., Yano Y., Matsuzaki K. (2020). Improvement of Therapeutic Index
by the Combination
of Enhanced Peptide Cationicity and Proline Introduction. ACS Infect Dis..

[312] Nishida M., Imura Y., Yamamoto M., Kobayashi S., Yano Y., Matsuzaki K. (2007). Interaction
of a magainin-PGLa hybrid
peptide with membranes: insight into the mechanism of synergism. Biochemistry..

[313] Starr C. G., Ghimire J., Guha S., Hoffmann J. P., Wang Y., Sun L., Landreneau B. N., Kolansky Z. D., Kilanowski-Doroh I. M., Sammarco M. C., Morici L. A., Wimley W. C. (2020). Synthetic molecular evolution of host cell-compatible,
antimicrobial peptides effective against drug-resistant, biofilm-forming
bacteria. Proc. Natl. Acad. Sci. U. S. A..

[314] Ghimire J., Hart R. J., Soldano A., Chen C. H., Guha S., Hoffmann J. P., Hall K. M., Sun L., Nelson B. J., Lu T. K., Kolls J. K., Rivera M., Morici L. A., Wimley W. C. (2023). Optimization of Host Cell-Compatible,
Antimicrobial Peptides Effective against Biofilms and Clinical Isolates
of Drug-Resistant Bacteria. ACS. Infect Dis..

[315] Imura Y., Choda N., Matsuzaki K. (2008). Magainin 2
in action: distinct modes of membrane permeabilization in living bacterial
and mammalian cells. Biophys. J..

[316] Kaji T., Yano Y., Matsuzaki K. (2021). In-Cell FRET
Indicates Magainin Peptide Induced Permeabilization of Bacterial Cell
Membranes at Lower Peptide-to-Lipid Ratios Relevant to Liposomal Studies. ACS Infect Dis..

[317] Leptihn S., Har J. Y., Chen J., Ho B., Wohland T., Ding J. L. (2009). Single molecule resolution of the
antimicrobial action of quantum dot-labeled sushi peptide on live
bacteria. BMC Biol..

[318] Cardoso M. H., Meneguetti B. T., Costa B. O., Buccini D. F., Oshiro K. G. N., Preza S. L. E., Carvalho C. M. E., Migliolo L., Franco O. L. (2019). Non-Lytic Antibacterial Peptides That Translocate Through
Bacterial Membranes to Act on Intracellular Targets. Int. J. Mol. Sci..

[319] Brogden K. A. (2005). Antimicrobial peptides: pore formers or metabolic inhibitors
in bacteria?. Nat. Rev. Microbiol..

[320] Scocchi M., Mardirossian M., Runti G., Benincasa M. (2015). Non-Membrane
Permeabilizing Modes of Action of Antimicrobial Peptides on Bacteria. Curr. Top Med. Chem..

[321] Welch N. G., Li W., Hossain M. A., Separovic F., O’Brien-Simpson N. M., Wade J. D. (2020). (Re)­Defining
the
Proline-Rich Antimicrobial Peptide Family and the Identification of
Putative New Members. Front Chem..

[322] Knappe D., Goldbach T., Hatfield M. P., Palermo N. Y., Weinert S., Strater N., Hoffmann R., Lovas S. (2016). Proline-rich
Antimicrobial Peptides Optimized for Binding to Escherichia coli Chaperone
DnaK. Protein Pept Lett..

[323] Zahn M., Kieslich B., Berthold N., Knappe D., Hoffmann R., Strater N. (2014). Structural identification
of DnaK
binding sites within bovine and sheep bactenecin Bac7. Protein Pept Lett..

[324] Zahn M., Berthold N., Kieslich B., Knappe D., Hoffmann R., Strater N. (2013). Structural studies
on the forward
and reverse binding modes of peptides to the chaperone DnaK. J. Mol. Biol..

[325] Berthold N., Hoffmann R. (2014). Cellular uptake of
apidaecin 1b and
related analogs in Gram-negative bacteria reveals novel antibacterial
mechanism for proline-rich antimicrobial peptides. Protein Pept Lett..

[326] Li W., Tailhades J., O’Brien-Simpson N. M., Separovic F., Otvos L., Hossain M. A., Wade J. D. (2014). Proline-rich
antimicrobial peptides: potential therapeutics against antibiotic-resistant
bacteria. Amino Acids..

[327] Cudic M., Otvos L. (2002). Intracellular targets
of antibacterial peptides. Curr. Drug Targets..

[328] Otvos L., de Olivier Inacio V., Wade J. D., Cudic P. (2006). Prior antibacterial
peptide-mediated inhibition of protein folding in bacteria mutes resistance
enzymes. Antimicrob. Agents Chemother..

[329] Chesnokova L. S., Slepenkov S. V., Witt S. N. (2004). The insect antimicrobial
peptide, L-pyrrhocoricin, binds to and stimulates the ATPase activity
of both wild-type and lidless DnaK. FEBS Lett..

[330] Florin T., Maracci C., Graf M., Karki P., Klepacki D., Berninghausen O., Beckmann R., Vazquez-Laslop N., Wilson D. N., Rodnina M. V., Mankin A. S. (2017). An antimicrobial
peptide that inhibits translation by trapping release factors on the
ribosome. Nat. Struct Mol. Biol..

[331] Mardirossian M., Grzela R., Giglione C., Meinnel T., Gennaro R., Mergaert P., Scocchi M. (2014). The host antimicrobial
peptide Bac71–35 binds to bacterial ribosomal proteins and
inhibits protein synthesis. Chem. Biol..

[332] Krizsan A., Volke D., Weinert S., Strater N., Knappe D., Hoffmann R. (2014). Insect-derived proline-rich antimicrobial
peptides kill bacteria by inhibiting bacterial protein translation
at the 70S ribosome. Angew. Chem., Int. Ed.
Engl..

[333] Graf M., Mardirossian M., Nguyen F., Seefeldt A. C., Guichard G., Scocchi M., Innis C. A., Wilson D. N. (2017). Proline-rich
antimicrobial peptides targeting protein synthesis. Nat. Prod Rep..

[334] Ghosh A., Kar R. K., Jana J., Saha A., Jana B., Krishnamoorthy J., Kumar D., Ghosh S., Chatterjee S., Bhunia A. (2014). Indolicidin targets duplex DNA: structural
and mechanistic insight through a combination of spectroscopy and
microscopy. ChemMedChem..

[335] Park C. B., Kim H. S., Kim S. C. (1998). Mechanism
of action
of the antimicrobial peptide buforin II: buforin II kills microorganisms
by penetrating the cell membrane and inhibiting cellular functions. Biochem. Biophys. Res. Commun..

[336] Abushahba M. F., Mohammad H., Thangamani S., Hussein A. A., Seleem M. N. (2016). Impact of different cell penetrating
peptides on the efficacy of antisense therapeutics for targeting intracellular
pathogens. Sci. Rep..

[337] Alajlouni R. A., Seleem M. N. (2013). Targeting listeria monocytogenes
rpoA and rpoD genes using peptide nucleic acids. Nucleic Acid Ther..

[338] Runti G., Lopez Ruiz M. d. C., Stoilova T., Hussain R., Jennions M., Choudhury H. G., Benincasa M., Gennaro R., Beis K., Scocchi M. (2013). Functional characterization
of SbmA, a bacterial inner membrane transporter required for importing
the antimicrobial peptide Bac7(1–35). J. Bacteriol..

[339] Mattiuzzo M., Bandiera A., Gennaro R., Benincasa M., Pacor S., Antcheva N., Scocchi M. (2007). Role of the Escherichia
coli SbmA in the antimicrobial activity of proline-rich peptides. Mol. Microbiol..

[340] Marlow V. L., Haag A. F., Kobayashi H., Fletcher V., Scocchi M., Walker G. C., Ferguson G. P. (2009). Essential
role for the BacA protein in the uptake of a truncated eukaryotic
peptide in Sinorhizobium meliloti. J. Bacteriol..

[341] Buck A. K., Elmore D. E., Darling L. E. (2019). Using fluorescence
microscopy to shed light on the mechanisms of antimicrobial peptides.
Future. Med. Chem..

[342] Park C. B., Yi K. S., Matsuzaki K., Kim M. S., Kim S. C. (2000). Structure-activity analysis of buforin
II, a histone H2A-derived antimicrobial peptide: the proline hinge
is responsible for the cell-penetrating ability of buforin II. Proc. Natl. Acad. Sci. U. S. A..

[343] Fitz-James P. C. (1958). Cytological and chemical studies
of the growth of protoplasts
of Bacillus megaterium. J. Biophys Biochem Cytol..

[344] Wei L., LaBouyer M. A., Darling L. E., Elmore D. E. (2016). Bacterial Spheroplasts
as a Model for Visualizing Membrane Translocation of Antimicrobial
Peptides. Antimicrob. Agents Chemother..

[345] Figueroa D. M., Wade H. M., Montales K. P., Elmore D. E., Darling L. E. O. (2018). Production
and Visualization of Bacterial Spheroplasts
and Protoplasts to Characterize Antimicrobial Peptide Localization. J. Vis Exp..

[346] Sun Y., Sun T. L., Huang H. W. (2014). Physical
properties of Escherichia
coli spheroplast membranes. Biophys. J..

[347] Wade H. M., Darling L. E. O., Elmore D. E. (2019). Hybrids made from
antimicrobial peptides with different mechanisms of action show enhanced
membrane permeabilization. Biochim Biophys Acta
Biomembr..

[348] Nonejuie P., Burkart M., Pogliano K., Pogliano J. (2013). Bacterial
cytological profiling rapidly identifies the cellular pathways targeted
by antibacterial molecules. Proc. Natl. Acad.
Sci. U. S. A..

[349] Scheinpflug K., Wenzel M., Krylova O., Bandow J. E., Dathe M., Strahl H. (2017). Antimicrobial peptide
cWFW kills
by combining lipid phase separation with autolysis. Sci. Rep..

[350] Hayden R. M., Goldberg G. K., Ferguson B. M., Schoeneck M. W., Libardo M. D., Mayeux S. E., Shrestha A., Bogardus K. A., Hammer J., Pryshchep S., Lehman H. K., McCormick M. L., Blazyk J., Angeles-Boza A. M., Fu R., Cotten M. L. (2015). Complementary
Effects of Host Defense Peptides Piscidin 1 and Piscidin 3 on DNA
and Lipid Membranes: Biophysical Insights into Contrasting Biological
Activities. J. Phys. Chem. B.

[351] Moniruzzaman M., Islam M. Z., Sharmin S., Dohra H., Yamazaki M. (2017). Entry of a Six-Residue Antimicrobial
Peptide Derived
from Lactoferricin B into Single Vesicles and Escherichia coli Cells
without Damaging their Membranes. Biochemistry..

[352] Omardien S., Drijfhout J. W., van Veen H., Schachtschabel S., Riool M., Hamoen L. W., Brul S., Zaat S. A. J. (2018). Synthetic
antimicrobial peptides delocalize membrane bound proteins thereby
inducing a cell envelope stress response. Biochim
Biophys Acta Biomembr..

[353] Yang Z., Choi H., Weisshaar J. C. (2018). Melittin-induced
Permeabilization, Re-sealing, and Re-permeabilization of *E.
coli* Membranes. Biophys. J..

[354] Rangarajan N., Bakshi S., Weisshaar J. C. (2013). Localized
permeabilization of E. coli membranes by the antimicrobial peptide
Cecropin A. Biochemistry..

[355] Sochacki K. A., Barns K. J., Bucki R., Weisshaar J. C. (2011). Real-time
attack on single Escherichia coli cells by the human antimicrobial
peptide LL-37. Proc. Natl. Acad. Sci. U. S.
A..

[356] Bakshi S., Choi H., Rangarajan N., Barns K. J., Bratton B. P., Weisshaar J. C. (2014). Nonperturbative
imaging of nucleoid morphology in live bacterial cells during an antimicrobial
peptide attack. Appl. Environ. Microbiol..

[357] Choi H., Yang Z., Weisshaar J. C. (2015). Single-cell,
real-time detection of oxidative stress induced in Escherichia coli
by the antimicrobial peptide CM15. Proc. Natl.
Acad. Sci. U. S. A..

[358] Barns K. J., Weisshaar J. C. (2016). Single-cell, time-resolved study
of the effects of the antimicrobial peptide alamethicin on Bacillus
subtilis. Biochim. Biophys. Acta.

[359] Choi H., Chakraborty S., Liu R., Gellman S. H., Weisshaar J. C. (2016). Single-Cell,
Time-Resolved Antimicrobial Effects of
a Highly Cationic, Random Nylon-3 Copolymer on Live Escherichia coli. ACS Chem. Biol..

[360] Choi H., Yang Z., Weisshaar J. C. (2017). Oxidative
stress induced in E. coli by the human antimicrobial peptide LL-37. PLoS Pathog..

[361] Agrawal A., Weisshaar J. C. (2018). Effects
of alterations of the E.
coli lipopolysaccharide layer on membrane permeabilization events
induced by Cecropin A. Biochim Biophys Acta
Biomembr..

[362] Agrawal A., Rangarajan N., Weisshaar J. C. (2019). Resistance
of early stationary phase E. coli to membrane permeabilization by
the antimicrobial peptide Cecropin A. Biochim
Biophys Acta Biomembr..

[363] Zhu Y., Mohapatra S., Weisshaar J. C. (2019). Rigidification
of the Escherichia
coli cytoplasm by the human antimicrobial peptide LL-37 revealed by
superresolution fluorescence microscopy. Proc.
Natl. Acad. Sci. U. S. A..

[364] Zhu Y., Weisshaar J. C., Mustafi M. (2020). Long-term effects of the proline-rich
antimicrobial peptide Oncocin112 on the Escherichia coli translation
machinery. J. Biol. Chem..

[365] Rank L. A., Agrawal A., Liu L., Zhu Y., Mustafi M., Weisshaar J. C., Gellman S. H. (2021). Diverse Impacts
on Prokaryotic and Eukaryotic Membrane Activities from Hydrophobic
Subunit Variation Among Nylon-3 Copolymers. ACS Chem. Biol..

[366] Zhu Y., Liu L., Mustafi M., Rank L. A., Gellman S. H., Weisshaar J. C. (2021). Local rigidification and possible
coacervation of the
Escherichia coli DNA by cationic nylon-3 polymers. Biophys. J..

[367] Landon C., Zhu Y., Mustafi M., Madinier J. B., Lelievre D., Aucagne V., Delmas A. F., Weisshaar J. C. (2022). Real-Time
Fluorescence Microscopy on Living E. coli Sheds New Light on the Antibacterial
Effects of the King Penguin beta-Defensin AvBD103b. Int. J. Mol. Sci..

[368] Cavaco M., Perez-Peinado C., Valle J., Silva R. D. M., Correia J. D. G., Andreu D., Castanho M., Neves V. (2020). To What Extent Do Fluorophores Bias the Biological Activity of Peptides?
A Practical Approach Using Membrane-Active Peptides as Models. Front
Bioeng. Biotechnol..

[369] Gelmi M. L., D’Andrea L. D., Romanelli A. (2020). Application
of Biophysical Techniques to Investigate the Interaction of Antimicrobial
Peptides With Bacterial Cells. Front Med. Technol..

[370] Yang Z., Weisshaar J. C. (2018). HaloTag
Assay Suggests Common Mechanism
of E. coli Membrane Permeabilization Induced by Cationic Peptides. ACS Chem. Biol..

[371] Wagstaff J. M., Balmforth M., Lewis N., Dods R., Rowland C., van Rietschoten K., Chen L., Harrison H., Skynner M. J., Dawson M., Ivanova-Berndt G., Beswick P. (2020). An Assay for Periplasm Entry Advances
the Development
of Chimeric Peptide Antibiotics. ACS Infect
Dis..

[372] Good L., Nielsen P. E. (1998). Antisense inhibition of gene expression
in bacteria by PNA targeted to mRNA. Nat. Biotechnol..

[373] Good L., Nielsen P. E. (1998). Inhibition of translation and bacterial
growth by peptide nucleic acid targeted to ribosomal RNA. Proc. Natl. Acad. Sci. U. S. A..

[374] Good L., Awasthi S. K., Dryselius R., Larsson O., Nielsen P. E. (2001). Bactericidal antisense effects of
peptide-PNA conjugates. Nat. Biotechnol..

[375] Bai H., You Y., Yan H., Meng J., Xue X., Hou Z., Zhou Y., Ma X., Sang G., Luo X. (2012). Antisense
inhibition of gene expression and growth in gram-negative bacteria
by cell-penetrating peptide conjugates of peptide nucleic acids targeted
to rpoD gene. Biomaterials..

[376] Ghosal A., Nielsen P. E. (2012). Potent antibacterial
antisense peptide-peptide
nucleic acid conjugates against Pseudomonas aeruginosa. Nucleic Acid Ther..

[377] Yau W. M., Wimley W. C., Gawrisch K., White S. H. (1998). The preference
of tryptophan for membrane interfaces. Biochemistry..

[378] Bechinger B. (1996). Towards membrane protein design:
pH-sensitive topology
of histidine-containing polypeptides. J. Mol.
Biol..

[379] Bechinger B. (2009). Rationalizing the membrane interactions of cationic
amphipathic antimicrobial peptides by their molecular shape. Curr. Opin. Colloid Interface Sci..

[380] Shai Y. (1999). Mechanism of the binding, insertion
and destabilization of phospholipid
bilayer membranes by alpha-helical antimicrobial and cell non-selective
membrane-lytic peptides. Biochim. Biophys. Acta.

[381] Ghose A. K., Viswanadhan V. N., Wendoloski J. J. (1998). Prediction
of hydrophobic (lipophilic) properties of small organic molecules
using fragmental methods: An analysis of ALOGP and CLOGP methods. Journal of Physical Chemistry A-Molecules, Spectroscopy, Kinetics,
Environment & General Theory..

[382] Manavalan B., Subramaniyam S., Shin T. H., Kim M. O., Lee G. (2018). Machine-Learning-Based
Prediction of Cell-Penetrating Peptides and
Their Uptake Efficiency with Improved Accuracy. J. Proteome Res..

[383] Pandey P., Patel V., George N. V., Mallajosyula S. S. (2018). KELM-CPPpred:
Kernel Extreme Learning Machine Based Prediction Model for Cell-Penetrating
Peptides. J. Proteome Res..

[384] Shi K., Xiong Y., Wang Y., Deng Y., Wang W., Jing B., Gao X. (2024). PractiCPP:
a deep learning approach
tailored for extremely imbalanced datasets in cell-penetrating peptide
prediction. Bioinformatics.

[385] Park H., Park J. H., Kim M. S., Cho K., Shin J. M. (2023). In Silico Screening and Optimization of Cell-Penetrating
Peptides Using Deep Learning Methods. Biomolecules..

[386] Bernardes-Loch R. M., de Oliveira Almeida G., Brasiliano I. T., Meira W., Pires D. E. V., Baracat-Pereira M. C., de Azevedo Silveira S. (2024). PerseuCPP:
a machine learning strategy to predict cell-penetrating
peptides and their uptake efficiency. Bioinform
Adv..

[387] Maroni G., Stojceski F., Pallante L., Deriu M. A., Piga D., Grasso G. (2025). LightCPPgen: An explainable machine
learning pipeline for rational design of cell penetrating peptides. Int. J. Antimicrob Agents..

[388] Manavalan B., Patra M. C. (2022). MLCPP 2.0: An Updated
Cell-penetrating
Peptides and Their Uptake Efficiency Predictor. J. Mol. Biol..

[389] Zhang X., Wei L., Ye X., Zhang K., Teng S., Li Z., Jin J., Kim M. J., Sakurai T., Cui L., Manavalan B., Wei L. (2023). SiameseCPP: a sequence-based Siamese network to predict cell-penetrating
peptides by contrastive learning. Brief Bioinform..

[390] Kumar N., Du Z., Li Y. (2025). pLM4CPPs:
Protein Language
Model-Based Predictor for Cell Penetrating Peptides. J. Chem. Inf Model..

[391] Chen Q., Zhang Y., Gao J., Zhang J. (2025). CPPCGM: A
Highly Efficient Sequence-Based Tool for Simultaneously Identifying
and Generating Cell-Penetrating Peptides. J.
Chem. Inf Model..

[392] Vihinen M. (2012). How to evaluate performance of prediction methods?
Measures and their interpretation in variation effect analysis. BMC Genomics..

[393] Wolfe J. M., Fadzen C. M., Choo Z. N., Holden R. L., Yao M., Hanson G. J., Pentelute B. L. (2018). Machine Learning To Predict Cell-Penetrating
Peptides for Antisense Delivery. ACS Cent Sci..

[394] Milo R., Jorgensen P., Moran U., Weber G., Springer M. (2010). BioNumbers--the
database of key numbers in molecular
and cell biology. Nucleic Acids Res..

[395] Nagaraj N., Wisniewski J. R., Geiger T., Cox J., Kircher M., Kelso J., Paabo S., Mann M. (2011). Deep proteome
and transcriptome mapping of a human cancer cell line. Mol. Syst. Biol..

[396] Carter E., Lau C. Y., Tosh D., Ward S. G., Mrsny R. J. (2013). Cell penetrating
peptides fail to induce an innate
immune response in epithelial cells in vitro: implications for continued
therapeutic use. Eur. J. Pharm. Biopharm..

[397] Saleh T., Bolhassani A., Shojaosadati S. A., Aghasadeghi M. R. (2015). MPG-based
nanoparticle: An efficient delivery system
for enhancing the potency of DNA vaccine expressing HPV16E7. Vaccine..

[398] Mehrlatifan S., Mirnurollahi S. M., Motevalli F., Rahimi P., Soleymani S., Bolhassani A. (2016). The structural
HCV genes delivered by MPG cell penetrating peptide are directed to
enhance immune responses in mice model. Drug
Delivery.

[399] Gitton Y., Tibaldi L., Dupont E., Levi G., Joliot A. (2009). Efficient CPP-mediated Cre protein delivery to developing
and adult CNS tissues. BMC Biotechnol..

[400] Lee J. H., Yang S. B., Park S. J., Kweon S., Ma G., Seo M., Kim H. R., Kang T. B., Lim J. H., Park J. (2025). Cell-Penetrating Peptide
Like Anti-Programmed Cell Death-Ligand 1
Peptide Conjugate-Based Self-Assembled Nanoparticles for Immunogenic
Photodynamic Therapy. ACS Nano.

[401] Moy P., Daikh Y., Pepinsky B., Thomas D., Fawell S., Barsoum J. (1996). Tat-mediated protein delivery can facilitate MHC class
I presentation of antigens. Mol. Biotechnol..

[402] Moschos S. A., Jones S. W., Perry M. M., Williams A. E., Erjefalt J. S., Turner J. J., Barnes P. J., Sproat B. S., Gait M. J., Lindsay M. A. (2007). Lung delivery studies using siRNA
conjugated to TAT(48–60) and penetratin reveal peptide induced
reduction in gene expression and induction of innate immunity. Bioconjug Chem..

[403] Szabo I., Orban E., Schlosser G., Hudecz F., Banoczi Z. (2016). Cell-penetrating conjugates of pentaglutamylated
methotrexate as potential anticancer drugs against resistant tumor
cells. Eur. J. Med. Chem..

[404] Movafegh B., Jalal R., Mohammadi Z., Aldaghi S. A. (2019). Poly-L-arginine: Enhancing Cytotoxicity and Cellular
Uptake of Doxorubicin and Necrotic Cell Death. Anticancer Agents Med. Chem..

[405] Duan Z., Chen C., Qin J., Liu Q., Wang Q., Xu X., Wang J. (2017). Cell-penetrating peptide
conjugates to enhance the antitumor effect of paclitaxel on drug-resistant
lung cancer. Drug Delivery.

[406] Cao Z., Liu L., Hu G., Bian Y., Li H., Wang J., Zhou Y. (2020). Interplay
of hydrophobic and hydrophilic
interactions in sequence-dependent cell penetration of spontaneous
membrane-translocating peptides revealed by bias-exchange metadynamics
simulations. Biochim Biophys Acta Biomembr..

[407] Verdine G. L., Hilinski G. J. (2012). Stapled peptides
for intracellular
drug targets. Methods Enzymol..

[408] Walensky L. D., Bird G. H. (2014). Hydrocarbon-stapled
peptides: principles,
practice, and progress. J. Med. Chem..

[409] Leshchiner E. S., Parkhitko A., Bird G. H., Luccarelli J., Bellairs J. A., Escudero S., Opoku-Nsiah K., Godes M., Perrimon N., Walensky L. D. (2015). Direct
inhibition
of oncogenic KRAS by hydrocarbon-stapled SOS1 helices. Proc. Natl. Acad. Sci. U. S. A..

[410] Liu X., Henriques S. T., Craik D. J., Chan L. Y. (2023). Unlocking the Potential
of the Antimicrobial Peptide Gomesin: From Discovery and Structure-Activity
Relationships to Therapeutic Applications. Int.
J. Mol. Sci..

[411] Somathilake U., Shang M., Nitsche C. (2025). De Novo Discovery of
Bicyclic Competitive Inhibitors of Zika Virus Protease from Peptide-Bismuth
Phage Display Screening. Chembiochem..

[412] Davies L. J., Ghosh P., Siryer S., Ullrich S., Nitsche C. (2025). Peptide-Bismuth Tricycles: Maximizing Stability by
Constraint. Chemistry..

[413] Ullrich S., Somathilake U., Shang M., Nitsche C. (2024). Phage-encoded
bismuth bicycles enable instant access to targeted bioactive peptides. Commun. Chem..

[414] Voss S., Adair L. D., Achazi K., Kim H., Bergemann S., Bartenschlager R., New E. J., Rademann J., Nitsche C. (2024). Cell-Penetrating Peptide-Bismuth Bicycles. Angew. Chem., Int. Ed. Engl..

[415] Habault J., Poyet J. L. (2019). Recent Advances
in Cell Penetrating
Peptide-Based Anticancer Therapies. Molecules..

[416] Ye J., Shin M. C., Liang Q., He H., Yang V. C. (2015). 15 years
of ATTEMPTS: a macromolecular drug delivery system based on the CPP-mediated
intracellular drug delivery and antibody targeting. J. Controlled Release.

[417] Park Y. J., Liang J. F., Song H., Li Y. T., Naik S., Yang V. C. (2003). ATTEMPTS: a heparin/protamine-based
triggered release system for the delivery of enzyme drugs without
associated side-effects. Adv. Drug Deliv Rev..

[418] Jearawiriyapaisarn N., Moulton H. M., Buckley B., Roberts J., Sazani P., Fucharoen S., Iversen P. L., Kole R. (2008). Sustained
dystrophin expression induced by peptide-conjugated morpholino oligomers
in the muscles of mdx mice. Mol. Ther..

[419] Echigoya Y., Nakamura A., Nagata T., Urasawa N., Lim K. R. Q., Trieu N., Panesar D., Kuraoka M., Moulton H. M., Saito T., Aoki Y., Iversen P., Sazani P., Kole R., Maruyama R., Partridge T., Takeda S., Yokota T. (2017). Effects of systemic multiexon skipping
with peptide-conjugated morpholinos in the heart of a dog model of
Duchenne muscular dystrophy. Proc. Natl. Acad.
Sci. U. S. A..

[420] Huang Y., Park Y. S., Wang J., Moon C., Kwon Y. M., Chung H. S., Park Y. J., Yang V. C. (2010). ATTEMPTS
system: a macromolecular prodrug strategy for cancer drug delivery. Curr. Pharm. Des..

[421] Liang J. F., Li Y. T., Yang V. C. (2000). A novel approach
for delivery of enzyme drugs: preliminary demonstration of feasibility
and utility in vitro. Int. J. Pharm..

[422] Shin M. C., Zhang J., Min K. A., Lee K., Moon C., Balthasar J. P., Yang V. C. (2014). Combination of antibody
targeting and PTD-mediated intracellular toxin delivery for colorectal
cancer therapy. J. Controlled Release.

[423] Jia L., Gorman G. S., Coward L. U., Noker P. E., McCormick D., Horn T. L., Harder J. B., Muzzio M., Prabhakar B., Ganesh B., Das Gupta T. K., Beattie C. W. (2011). Preclinical pharmacokinetics,
metabolism, and toxicity of azurin-p28 (NSC745104) a peptide inhibitor
of p53 ubiquitination. Cancer Chemother Pharmacol..

[424] Warso M. A., Richards J. M., Mehta D., Christov K., Schaeffer C., Rae Bressler L., Yamada T., Majumdar D., Kennedy S. A., Beattie C. W., Das Gupta T. K. (2013). A first-in-class,
first-in-human, phase I trial of p28, a non-HDM2-mediated peptide
inhibitor of p53 ubiquitination in patients with advanced solid tumours. Br J. Cancer..

[425] Punj V., Bhattacharyya S., Saint-Dic D., Vasu C., Cunningham E. A., Graves J., Yamada T., Constantinou A. I., Christov K., White B., Li G., Majumdar D., Chakrabarty A. M., Das Gupta T. K. (2004). Bacterial
cupredoxin azurin as an inducer of apoptosis and regression in human
breast cancer. Oncogene..

[426] Yamada T., Fialho A. M., Punj V., Bratescu L., Gupta T. K., Chakrabarty A. M. (2005). Internalization
of bacterial redox
protein azurin in mammalian cells: entry domain and specificity. Cell Microbiol..

[427] Lulla R. R., Goldman S., Yamada T., Beattie C. W., Bressler L., Pacini M., Pollack I. F., Fisher P. G., Packer R. J., Dunkel I. J., Dhall G., Wu S., Onar A., Boyett J. M., Fouladi M. (2016). Phase I trial of p28
(NSC745104), a non-HDM2-mediated peptide inhibitor of p53 ubiquitination
in pediatric patients with recurrent or progressive central nervous
system tumors: A Pediatric Brain Tumor Consortium Study. Neuro Oncol..

[428] Taylor B. N., Mehta R. R., Yamada T., Lekmine F., Christov K., Chakrabarty A. M., Green A., Bratescu L., Shilkaitis A., Beattie C. W., Das Gupta T. K. (2009). Noncationic
peptides obtained from azurin preferentially enter cancer cells. Cancer Res..

[429] Yamada T., Mehta R. R., Lekmine F., Christov K., King M. L., Majumdar D., Shilkaitis A., Green A., Bratescu L., Beattie C. W., Das Gupta T. K. (2009). A peptide
fragment of azurin induces a p53-mediated cell cycle arrest in human
breast cancer cells. Mol. Cancer Ther..

[430] Pae J., Saalik P., Liivamagi L., Lubenets D., Arukuusk P., Langel Ü, Pooga M. (2014). Translocation of cell-penetrating
peptides across the plasma membrane is controlled by cholesterol and
microenvironment created by membranous proteins. J. Controlled Release.

[431] Fanghanel S., Wadhwani P., Strandberg E., Verdurmen W. P. R., Burck J., Ehni S., Mykhailiuk P. K., Afonin S., Gerthsen D., Komarov I. V., Brock R., Ulrich A. S. (2014). Structure analysis and conformational transitions of
the cell penetrating peptide transportan 10 in the membrane-bound
state. PLoS One..

[432] Ezzat K., EL Andaloussi S., Zaghloul E. M., Lehto T., Lindberg S., Moreno P. M. D., Viola J. R., Magdy T., Abdo R., Guterstam P., Sillard R., Hammond S. M., Wood M. J. A., Arzumanov A. A., Gait M. J., Smith C. I. E., Hallbrink M., Langel U. (2011). PepFect 14, a novel cell-penetrating
peptide for oligonucleotide delivery in solution and as solid formulation. Nucleic Acids Res..

[433] Anko M., Majhenc J., Kogej K., Sillard R., Langel Ü, Anderluh G., Zorko M. (2012). Influence of stearyl
and trifluoromethylquinoline modifications of the cell penetrating
peptide TP10 on its interaction with a lipid membrane. Biochimica et Biophysica Acta - Biomembranes..

[434] El Andaloussi S., Lehto T., Lundin P., Langel U. (2011). Application
of PepFect peptides for the delivery of splice-correcting oligonucleotides. Cell-Penetrating Peptides.

[435] Ervin E. H., Pook M., Teino I., Kasuk V., Trei A., Pooga M., Maimets T. (2019). Targeted gene silencing
in human embryonic stem cells using cell-penetrating peptide PepFect
14. Stem Cell Res. Ther..

[436] Gestin M., Helmfors H., Falato L., Lorenzon N., Michalakis F. I., Langel U. (2020). Effect of small molecule
signaling
in PepFect14 transfection. PLoS One..

[437] van Asbeck A. H., Beyerle A., McNeill H., Bovee-Geurts P. H. M., Lindberg S., Verdurmen W. P. R., Hallbrink M., Langel Ü, Heidenreich O., Brock R. (2013). Molecular parameters
of siRNA-cell penetrating peptide nanocomplexes for efficient cellular
delivery. ACS Nano.

[438] Carreras-Badosa G., Maslovskaja J., Periyasamy K., Urgard E., Padari K., Vaher H., Tserel L., Gestin M., Kisand K., Arukuusk P., Lou C., Langel U., Wengel J., Pooga M., Rebane A. (2020). NickFect type
of cell-penetrating peptides present enhanced efficiency for microRNA-146a
delivery into dendritic cells and during skin inflammation. Biomaterials..

[439] Regberg J., Vasconcelos L., Madani F., Langel U., Hallbrink M. (2016). pH-responsive
PepFect cell-penetrating peptides. Int. J. Pharm..

[440] Vasconcelos L., Madani F., Arukuusk P., Parnaste L., Graslund A., Langel U. (2014). Effects of cargo molecules on membrane
perturbation caused by transportan10 based cell-penetrating peptides. Biochimica et Biophysica Acta - Biomembranes..

[441] Ezzat K., Zaghloul E. M., El Andaloussi S., Lehto T., El-Sayed R., Magdy T., Smith C. I., Langel U. (2012). Solid formulation of
cell-penetrating peptide nanocomplexes
with siRNA and their stability in simulated gastric conditions. J. Controlled Release.

[442] Suhorutsenko J., Oskolkov N., Arukuusk P., Kurrikoff K., Eriste E., Copolovici D. M., Langel U. (2011). Cell-penetrating peptides,
PepFects, show no evidence of toxicity and immunogenicity in vitro
and in vivo. Bioconjug Chem..

[443] Brock R. (2014). The uptake of arginine-rich cell-penetrating
peptides: putting the
puzzle together. Bioconjug Chem..

[444] Herce H. D., Schumacher D., Schneider A. F. L., Ludwig A. K., Mann F. A., Fillies M., Kasper M. A., Reinke S., Krause E., Leonhardt H., Cardoso M. C., Hackenberger C. P. R. (2017). Cell-permeable nanobodies for targeted
immunolabelling and antigen manipulation in living cells. Nat. Chem..

[445] Duchardt F., Fotin-Mleczek M., Schwarz H., Fischer R., Brock R. (2007). A comprehensive
model for the cellular uptake of cationic cell-penetrating
peptides. Traffic..

[446] Ter-Avetisyan G., Tunnemann G., Nowak D., Nitschke M., Herrmann A., Drab M., Cardoso M. C. (2009). Cell entry of arginine-rich
peptides is independent of endocytosis. J. Biol.
Chem..

[447] Iwata T., Hirose H., Sakamoto K., Hirai Y., Arafiles J. V. V., Akishiba M., Imanishi M., Futaki S. (2021). Liquid Droplet
Formation and Facile Cytosolic Translocation of IgG in the Presence
of Attenuated Cationic Amphiphilic Lytic Peptides. Angew. Chem., Int. Ed. Engl..

[448] Hirai Y., Kawaguchi Y., Kasahara C., Hirose H., Futaki S. (2024). Liquid Droplet-Mediated
Formulation of Lipid Nanoparticles
Encapsulating Immunoglobulin G for Cytosolic Delivery. Mol. Pharmaceutics.

[449] Sun Y., Lau S. Y., Lim Z. W., Chang S. C., Ghadessy F., Partridge A., Miserez A. (2022). Phase-separating peptides for direct
cytosolic delivery and redox-activated release of macromolecular therapeutics. Nat. Chem..

[450] Trevellin G. G., Kwag J. Y., Shui M. L., Klim H., Alvarez V., Elmore D. E. (2024). ACS Med. Chem. Lett..

